# Latent HIV Infection of BBB Pericytes Enhances Gap Junction-mediated Crosstalk with Endothelial Cells

**DOI:** 10.1007/s12035-026-06266-0

**Published:** 2026-10-16

**Authors:** Sarah Schmidlin, Oandy Naranjo, Silvia Torices, Nikolai Fattakhov, Monika Gilicinska, Masha Minevich, Kyra A. Schindler, Destiny Tiburcio, Olivia M. Osborne, Arpan Acharya, Minseon Park, Mario Stevenson, Siddappa N. Byrareddy, Michal Toborek

**Affiliations:** 1 Department of Biochemistry and Molecular Biology, University of Miami Leonard M. Miller School of Medicine, Suite 528, 1011 NW 15Th Street, Miami, FL 33136 USA; 2 Medical Scientist Training Program, University of Miami Leonard M. Miller School of Medicine, Miami, FL USA; 3 Sylvester Comprehensive Cancer Center, University of Miami Leonard M. Miller School of Medicine, Miami, FL USA; 4 Department of Pharmacology & Experimental Neuroscience, University of Nebraska Medical Center, Omaha, NE USA; 5 Department of Medicine, University of Miami Leonard M. Miller School of Medicine, Miami, FL USA

**Keywords:** HIV infection, Blood-brain barrier, Antiretroviral therapy, Neurocognitive impairment, Central nervous system, Gap junction-mediated communication

## Abstract

HIV-Associated Neurocognitive Impairments persist, with over 50% of patients experiencing cognitive decline. Blood-brain barrier (BBB) impairment is a consistent feature of this comorbidity, which is remarkable, because BBB endothelial cells are not productively infected by HIV-1. Conversely, pericytes can be productively infected with HIV-1 and are essential for cerebrovascular homeostasis. Given this, we hypothesized that HIV-1 infection of pericytes disrupts BBB endothelial cell integrity through dysfunctional gap junction and hemichannel signaling, impairing pericyte-endothelial intercellular communication. Using a co-culture model with primary human BBB pericytes and brain microvascular endothelial cells, pericytes were infected with HIV-1 for 3 or 7 days, exhibiting active and latent viral phenotypes. Findings revealed enhanced gap junction communication between HIV-infected pericytes and endothelial cells under both conditions, and blocking gap junctions modulated endothelial inflammatory responses. Overall, this study advances our understanding of the molecular mechanisms underlying HIV-associated BBB dysfunction and may inform the development of targeted therapeutic strategies.

**Supplementary Information:**

The online version contains supplementary material available at https://doi.org/10.1007/s12035-026-06266-0.

## Introduction

Despite significant advancements in antiretroviral therapy (ART), Human Immunodeficiency Virus type-1 (HIV-1) infection remains a global disease, with around 50% of people living with HIV (PLWH) exhibiting various signs of neurocognitive impairment (NCI) [1–3]. The mechanisms behind HIV-associated NCI are suspected to include chronic inflammation, oxidative stress, and viral particle neurotoxicity; however, the precise mechanisms are unknown [2]. HIV-1 passes through the blood-brain barrier (BBB) 1–2 weeks after infection, establishing the central nervous system (CNS) infection and allowing the HIV-1 provirus to integrate within the host-cell genome of brain cells [4]. Persistent CNS infection through latent viral reservoirs and low-level chronic neuroinflammatory responses likely play a role in the development of NCI [4]. These HIV reservoirs can lead to cyclical rebounds of viremia and provide the basis for the development of neurocognitive impairment [5].

The BBB is organized into repeating function units called neurovascular units (NVUs), each formed by endothelial cells, pericytes, astrocytes, microglia, and neurons. These cells form a dynamic interactome, continuously signaling to one another to regulate barrier permeability, control cerebral blood flow, cell metabolism, neuronal functions, and preserve central nervous system homeostasis. Thus, BBB integrity depends not just on the endothelial cells themselves, but on coordinated communication across the entire NVU. While microglia are considered the primary HIV reservoir in the brain, studies have also demonstrated that astrocytes and pericytes can support productive HIV-1 replication [6–11]. In addition, studies have suggested the capacity of pericytes to harbor latent HIV-1 infection, implying their potential to remain a latent reservoir in the CNS [6, 12, 13]. Pericytes are highly specialized, multifunctional cells essential for maintaining the BBB microenvironment. They play a critical role in angiogenesis, regulation of vascular diameter and capillary blood flow, as well as supporting the basement membrane and endothelial cell integrity [14]. Pericytes are embedded in the basement membrane of BBB microvessels and communicate with endothelial cells through both direct interactions and paracrine signaling. Notably, pericytes are the only cells in the NVU that establish direct physical contacts with endothelial cells due to their shared location within the basement membrane [15]. These contacts occur via peg-and-socket-type interactions in which cytoplasmic protrusions (the “peg”) from one cell fit tightly into corresponding invaginations (the “socket”) of the other. This contact is bi-directional, with both cell types forming pegs and sockets, creating a reciprocal, interlocking connection. Additionally, adhesion proteins anchor pericytes to endothelial cells, and gap junction (GJ) channels enable the exchange of ions and small molecules between these cells [16–18].

Gap junctions are aggregates of intracellular channels that provide direct continuity between the cytoplasm of two or more cells, allowing for the transfer of ions and intracellular messengers [19, 20]. A gap junction is formed when two hemichannels (HC), each formed by an assembly of six connexin proteins, on the cell membrane dock head-to-head. In addition, cells express unopposed HCs (uHC), formed by connexin or pannexin proteins, that serve as a means of metabolite exchange between the cytoplasm and the extracellular environment [21]. These channels allow for autocrine and paracrine signaling via diffusion of ions and small molecules such as ATP, calcium, and neurotransmitters [21]. In general, the response to pathological conditions such as stress or viral infections is the closing of GJ channels to contain cell injury [19, 22]. Moreover, hemichannels remain closed under normal physiological conditions but open in response to cellular stress or inflammation, such as during viral or pathogenic infections [23, 24]. Several studies have shown that HIV-infected astrocytes had a significant effect on the opening of HCs, BBB integrity, and endothelial cell apoptosis via GJ-dependent mechanisms [20, 21, 25–27]. In addition, GJ-mediated intercellular communication has been shown to play a crucial role in the spread of HIV-1 infection from infected BBB pericytes to neighboring pericytes [12]. Enhanced GJ- and HC-mediated communication plays a pivotal role in the pathogenesis of infectious diseases by facilitating the spread of infection and toxic signals between cells [19].

Despite progress in understanding pericyte infection by HIV-1, its functional impact on BBB integrity remains largely unknown, which is critical given that BBB impairment is consistently observed in HIV-associated NCI and correlates with its presence and progression [28–30]. Importantly, BBB endothelial cells are not productively infected by HIV-1; however, we observe marked BBB dysregulation in HIV infection. BBB pericytes play a crucial role in maintaining barrier integrity and mediating HIV CNS infection by signaling endothelial cells via GJ and HC coupling. Therefore, we sought in the present study to characterize the impact of active and latent pericyte infection on GJ- and HC-mediated communication with endothelial cells. We demonstrate that altered intercellular communication with endothelial cells has been executed not only by actively but also by latently HIV-1 infected pericytes, providing novel insight about the importance of CNS HIV reservoirs.

## Materials and Methods

### HIV-1 Production and Infection

The pNL4-3 viral plasmid was obtained from the NIH AIDS Research and Reference Reagent Program (Division of AIDS, NIAID, National Institutes of Health). The plasmid was amplified in One Shot TOP10 chemically competent E. coli cells (Thermo Fisher Scientific, Cat. No. C404003) and purified using the EndoFree Plasmid Maxi Kit (QIAGEN, Cat. No. 12362). Viral stocks were generated by transfecting 6.5 × 10^6^ HEK293T cells with 20 to 30 μg of proviral plasmid using the ProFection Mammalian Transfection System (Promega, Cat. No. E1200). The following day, the medium was changed to Opti-Mem (Thermo Fisher Scientific, Cat. No. 11058-021). 48 h later, the cell culture supernatants were collected, filtered using a 0.45-μm pore size filter (Millipore Sigma, Cat. No. 430314), and concentrated using weight exclusion columns (Millipore Sigma, Cat. No. UFC905024). Viral stocks were stored at −80 °C, and p24 antigen concentration was measured by using the HIV-1 p24 Antigen ELISA kit (Abcam, Cat. No. ab218268) according to the manufacturer’s instructions. Primary human pericytes were infected with HIV-1 NL4-3 by incubation with 60 ng p24 HIV-1 NL4-3/ml for 24 h, followed by washing with DPBS and replacement with fresh pericyte medium.

### Cell Cultures

Primary human brain microvascular endothelial cells (HBMECs) (Cell Systems, Cat. No. ACBRI 376) were cultured in complete classic medium with serum and CultureBoost (Cell Systems, Cat. No. 4Z0-500). Primary human brain vascular pericytes (ScienCell, Cat. No. 1200) were cultured in pericyte medium (ScienCell, Cat. No. 1201) supplemented with 2% fetal bovine serum (FBS), 0.2% pericyte growth supplement, 100 units/ml penicillin, and 100 μg/ml streptomycin. Primary HBMECs were used from passage 3 to 11, and primary human brain vascular pericytes were used from passage 3 to 7. Human embryonic kidney 293 T (HEK293T) cells were purchased from ATCC (ATCC, Cat. No. CRL-3216) and cultured in Dulbecco’s modified Eagle medium (DMEM) (Thermo Fisher Scientific, Cat. No. 11995073) supplemented with 10% FBS (ATTC, Cat. No. 30–2020) and 1% penicillin-streptomycin (10,000 U/mL) (Thermo Fisher Scientific, Cat. No. 15140122). Cell cultures were maintained in 5% of CO_2_ at 37 °C.

### Endothelial/Pericyte Co-culture Model

Endothelial/pericyte co-culture model mimicking the cell-cell interactions in brain capillaries was established using human primary HBMECs and brain vascular pericytes as previously described by our laboratory [31]. Pericytes were seeded on the underside of permeable clear polyester Transwell inserts with a pore size of 0.4 μm (Corning, Cat. No. 3450) and allowed to attach overnight. The next day, the inserts were flipped over into TPP 6- well plates (MidSci, Cat. No. TP92406) containing 2 mL of pericyte medium. HBMECs were seeded on the top of the insert and allowed to attach overnight. The following day, pericytes in the co-culture system were selectively infected with HIV-1 NL4-3 (60 ng p24/mL). Pericyte medium was removed and replaced with 2 mL of fresh medium containing virus, whereas control cultures received fresh virus-free pericyte medium. To maintain identical culture conditions, the endothelial cell compartment was replenished with fresh virus-free medium in both infected and control co-cultures. After 24 h, the cells were washed with DPBS and replaced with fresh pericyte and endothelial medium. Co-cultures were maintained for the designated experiment time (3 to 7 days). Cell seeding density depended upon the number of days the cells were cultured. Cell culture medium was changed 24 h after infection and every 2 days to replenish nutrients.

### Human Brain Tissue

Post-mortem human brain tissue was obtained through the National NeuroAIDS Tissue Consortium (NNTC) Data Coordinating Center. Snap-frozen temporal lobe tissue blocks (1 cm^3^) were used for analyses. The cohort included two cases: one HIV-seronegative control and one HIV-seropositive individual with HIV-associated neurocognitive disorder (HAND) and confirmed antiretroviral-mediated viral suppression. Exclusion criteria included history of illicit substance abuse at last visit, hemorrhage of the dura or leptomeninges, and bacterial infection. Associated clinical and laboratory data, including CD4 count, plasma HIV RNA, and cerebrospinal fluid (CSF) HIV RNA, were obtained for each case. All tissue use was conducted under an approved human subjects protocol in accordance with institutional IRB guidelines.

### Microvessel Extraction and RNAscope™ Multiplex Fluorescent In Situ Hybridization

Human brain microvessels were extracted from post-mortem human brain tissue according to our previously established protocol [32]. Human brain microvessels were then processed for multiplex fluorescent in situ hybridization using the RNAscope™ Multiplex Fluorescent v2 Assay (Advanced Cell Diagnostics, Bio-Techne) according to the manufacturer’s manual protocol. A 4-plex probe panel was used to simultaneously detect HIV nucleic acids and vascular cell identity markers within the same tissue section. HIV Clade B proviral DNA was detected using the sense-strand probe targeting the gag-pol region (V-HIV1-CladeB-gag-pol-sense, Cat. No. 444058; GenBank AF324493.2, nt 854–3940, 40 ZZ pairs) in Channel 1 (Opal 520). Active HIV transcription was assessed using a probe spanning the regulatory and accessory gene region including vif, vpr, tat, rev, vpu, env, nef, and tar (V-HIV1-CladeB-vif-vpr-tat-rev-vpu-env-nef-tar-C2, Cat. No. 444068-C2) in Channel 2 (Opal 570). Pericyte identity was assessed using a probe targeting PDGFRB (Hs-PDGFRB-C3, Cat. No. 548998-C3) in Channel 3 (Opal 620), and endothelial cells were labeled using a probe targeting PECAM1/CD31 (Hs-PECAM1-O1-C4, Cat. No. 487388-C4) in Channel 4 (Opal 690). RNA integrity and assay performance were validated in all samples using a 4-plex housekeeping gene positive control probe set (Hs-PPIB-C1, Cat. No. 313908; Hs-POLR2A-C2, Cat. No. 310458-C2; Hs-UBC-C3, Cat. No. 310048-C3; Hs-HPRT1-C4, Cat. No. 310348-C4) and a Bacillus subtilis dapB negative control probe (Cat. No. 311838). Images were acquired using the Olympus FluoView 1200 Laser Scanning Confocal Microscope (Olympus). All images were processed with equivalent adjustments to brightness and contrast across all channels to uniformly reduce autofluorescence background.

### RNAscope™

RNAscope analysis was performed on fixed cell culture slides using the RNAscope™ Multiplex Fluorescent V2 Assay (Advanced Cell Diagnostics, Cat. No. 323100). We utilized RNAscope Probe- V-HIV1-CladeB-vif-vpr-tat-rev-vpu-env-nef tarC2-HIV-1 NL4-3 complete sequence (ACD, Cat. No. 444051) for detection of viral RNA and RNAscope Probe-V-HIV1-CladeB-gag-pol sense-HIV-1 NL4-3 (ACD, Cat. No. 444061-C2) for viral DNA. RNAscope pretreatment was performed as previously described [33]. Amplification and detection were carried out according to the Advanced Cell Diagnostics protocol for the RNAscope Multiplex Fluorescent V2 Assay. Opal 520 and 570 fluorophore reagent packs were utilized to visualize the bound probes (Akoya, Cat. No. FP1487001KT and FP1488001KT), and DAPI (included in the RNAscope assay kit) was used for nuclear localization.

### HIV-1 DNA Quantitative PCR

HIV-1 DNA was quantified using Azure Biosystems’ Cielo 6 real-time qPCR instrument. All oligonucleotide primers and probes were based on the sequence of LAI/BRU (GenBank accession number K02013). The reagents target and amplify a region of the genome from the Nef gene to the long terminal repeat. The forward primer sequence is LAI^8634^ *AGGCAGCTGTAGATCTTAGCCACT*, the reverse primer sequence is LAI^9347^ *AACAGACGGGCACACACTACTTTG,* and that of the fluorogenic reporter probe is LAI^9081^*56-FAM-AGT GGC GAG CCC TCA GAT GCT GC*-*3IABkFQ* (Integrated DNA Technologies). Magnesium chloride (63,068) and oligonucleotides were from Sigma-Aldrich, and deoxyribonucleotide triphosphates (N0447S) were from New England Biolabs.

Extracted DNA using the QIAamp DNA Mini Kit (Qiagen Cat. No. 51304) was amplified in 30 µL reactions containing 1X HiDi Taq buffer, 200 μM dNTPs, 400 nM primers, 250 nM fluorogenic reporter probe, 0.5 mM additional MgCl_2_, and 1.5 units HiDi Taq (Medix Biochemica). After an initial denaturation at 95 °C for 2 min, the HIV-1 target was amplified by cycling 45 times at 95 °C for 10 s, 62 °C for 15 s, and 72 °C for 45 s. Quantities of unknowns were determined by extrapolation from a standard curve generated by using a ten-fold dilution series of a defined synthetic standard and the Cielo 6 software.

### HIV-1 Gag Gene Quantification

HIV-1 Gag mRNA in infected human pericytes was quantified using reverse transcription real-time quantitative PCR (RT-qPCR). Total RNA was extracted from pericytes in co-culture using the RNeasy Mini Kit (Qiagen, Cat. No. 74104) according to the manufacturer’s protocol. First-strand cDNA synthesis and PCR amplification were carried out in the same tube using qScript XLT One-Step RT-qPCR ToughMix (Quanta Bioscences, Cat. No. 95134-500), with 100 ng of RNA per reaction. Reactions were conducted on the 7500 Fast Real-Time PCR System (Applied Biosystems). Primers and a fluorogenic probe were specifically designed to target HIV-1 Gag region, and they were as follows: gag-Forward primer, 50-GAC ATA AGA CAG GGA CCA AAG G-30; gag Reverse primer, 50-CTG GGT TTG CAT TTT GGA CC-30; probe, 50-AAC TCT AAG AGC CGA GCA AGC TTC AC-30. All reactions were conducted in duplicates, and the relative quantification of Gag gene expression was performed using the 2-ΔΔCT method. Normalization was performed using the housekeeping gene GAPDH as a reference (Thermo Fisher Scientific, Cat. No. 4310884E).

### Alu-gag PCR and Nucleotide Sequencing

**Fig. 1:**
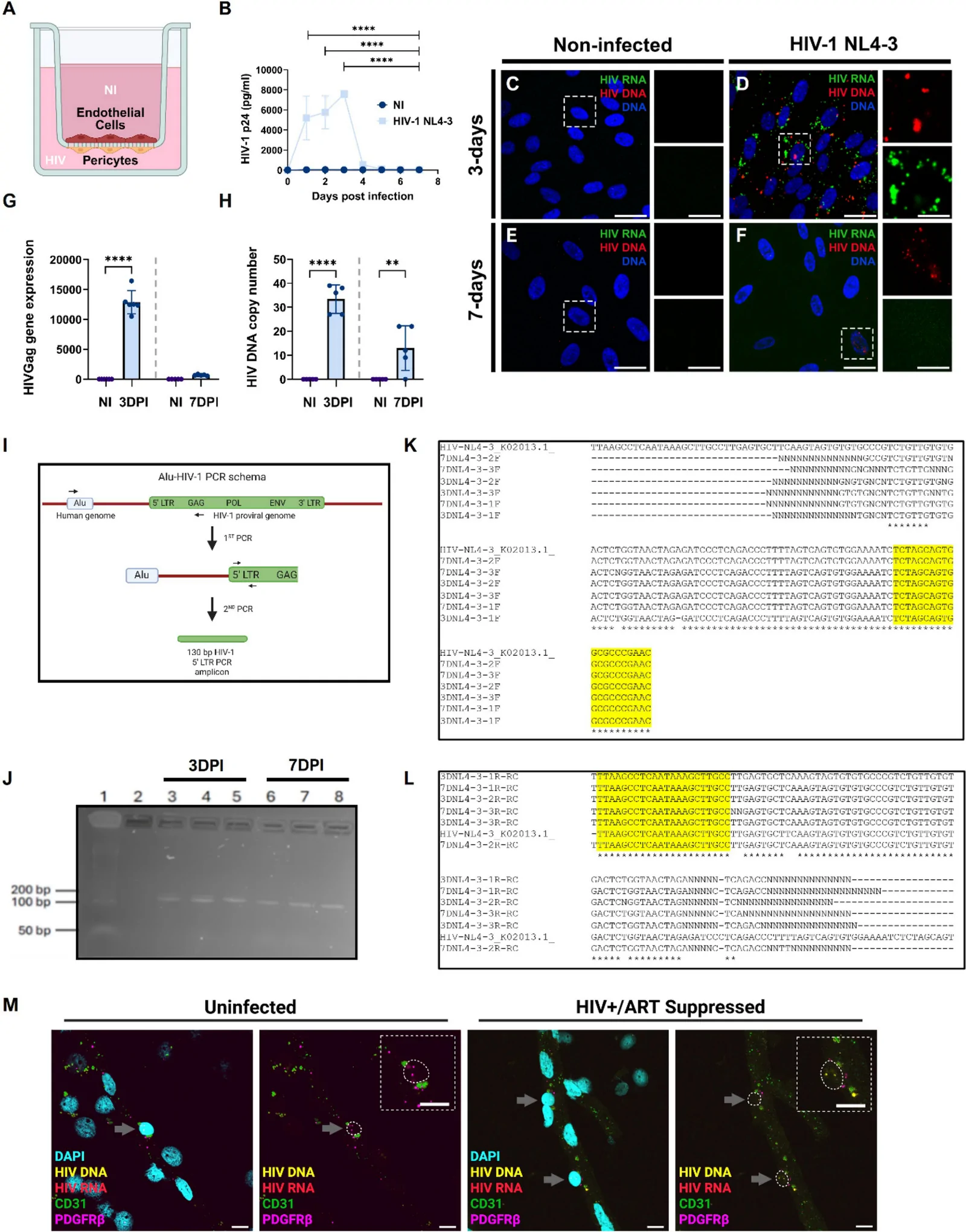
Active and latent viral phenotypes of BBB pericytes in co-culture with brain microvascular endothelial cells. **A** Co-culture model of BBB pericytes and brain microvascular endothelial cells seeded on either side of a PET membrane insert, pore size 0.4 µm. **B** p24 release into pericyte culture media at the indicated days post-infection. Graphs indicate the mean ± SD from 3 to 6 independent experiments. **C**, **D**, **E**, and **F** RNAscope analysis of HIV DNA (red), HIV RNA (green), and cell nuclei (blue) in pericytes at 3 and 7 days post-infection (DPI). Image inlets are magnified 4× and show split colors for HIV DNA (upper inlet) and HIV RNA (lower inlet), scale bars = 30 μm and 12 μm for inlets. **G** HIVGag expression levels in pericytes measured by RT-qPCR and **H** HIV DNA copy number measured by qPCR. Graphs indicate the mean ± SD from 5 to 6 independent experiments. **I** Experimental schematic of 2-step Alu-Gag PCR. **J** Agarose gel image of the nested PCR amplicon (Lane 1: 100 bp DNA ladder; Lane 2: No template control; Lane 3–5: Pericyte genomic DNA isolated at 3 DPI; Lane 6–8: Pericyte genomic DNA isolated at 7 DPI). Sanger sequencing of PCR amplicon with multiple sequence alignment to HIV-NL4-3 (K02013.1) reference sequence using Clustal W: **K** multiple-aligned sequence using forward primer (the reverse complement of the reverse primer sequence is highlighted in yellow), and **L** the multiple-aligned sequences of the reverse complement of the sequences generated using the reverse primers (the forward primer sequence is highlighted in yellow). **M** RNAscope analysis of human temporal cortex microvessels from an HIV-seronegative control (left panel) and an HIV-positive individual on long-term antiretroviral therapy (right panel) with DAPI (cyan), HIV DNA (yellow), HIV RNA (red), CD31 (green), and PDGFRβ (magenta), scale bars = 10 μm. Arrows point to PDGFRβ positive cells and white dotted lines outline nuclei and inlet zoomed images. Note the presence of HIV DNA in the nuclei of HIV-infected pericytes (overlapping yellow and magenta colors). **p < 0.01, ****p < 0.0001

We employed a two-step *Alu-gag* PCR assay for detection and quantitation of integrated HIV-DNA, as previously described [34, 35]. Total DNA was isolated using the QIAamp DNA Mini Kit (Qiagen Cat. No. 51304). The first PCR was performed with one primer that anneals to *Alu* and the other that anneals to *gag*. *Alu* is a repeat element in the human genome that occurs approximately every 5000 base pairs, and *Gag* is an HIV-1 gene that encodes for the structural components of the virion particles. The second PCR detects HIV-specific products using primers to the R and U5 regions within the HIV-1 long terminal repeat (LTR). The nested PCR method is depicted schematically in Fig. 1. The PCR amplicons were purified using a QIAGEN PCR purification kit (QIAGEN, Germany) per the manufacturer’s instructions and subjected to Sanger sequencing using BigDye Terminator v1.1 Cycle Sequencing Kit (Applied Biosystems, California, USA). The raw sequence data were manually edited, and multiple sequence alignment of the edited sequence was performed with HIV-NL4-3 (K02013.1) reference sequence using Clustal W.

### TEER and Permeability Assay

Transendothelial electrical resistance (TEER), an indicator of barrier integrity, was assessed by an EVOM2 epithelial voltohmmeter (World Precision Instruments) as previously described [31]. The electrodes were sterilized in 70% isopropyl alcohol for 30 min and then placed in DPBS for 30 min to calibrate the instrument. The long prong of the electrode was inserted in one of the three openings in the well insert, allowing the short prong to rest in the medium on the top of the insert. Electrodes were held at a 90-degree angle to the well insert, and three readings were taken per well. The TEER values were calculated by subtracting the mean ohm value of the blank and multiplying the resulting resistance value by the area of the membrane insert (cm^2^) to give the reported TEER value in Ω∙cm^2^.

The endothelial/pericyte barrier integrity was also assessed by the permeability of 20-kDa and 40-kDa fluorescein isothiocyanate (FITC)-dextran. 0.5 mg/mL 20-kDa (Millipore Sigma Cat. No. FD20S) or 40-kDa (Cat. No. FD40S) FITC-dextran was dissolved in phenol red-free EBM medium (Lonza, Cat. No. CC 3129). The transwell inserts were washed with HBSS (ThermoFisher, Cat. No. 14175095) twice and moved into new 6-well plates containing 2 mL of phenol red-free EBM medium. One mL of FITC-dextran solution was added to the upper compartment of each insert and incubated for 1 h at 37 °C. Following incubation, 100 μL of medium was collected from the lower compartment and transferred to 96-well CellBIND microplates (Corning, Cat. No. 3340). Fluorescence was determined with Spectramax iD3 microplate reader (Molecular Devices) using excitation and emission wavelengths of 480 nm and 530 nm, respectively.

### Protein Extraction and Immunoblotting

Cells were lysed with a RIPA lysis buffer system containing protease inhibitors (Santa Cruz Biotechnology Cat. No. sc-24948). Total protein concentrations were measured by a BCA Protein Assay Kit (Thermofisher Cat. No. 23223 for Reagent A, Cat. No. 23224 for Reagent B, and Cat. No. 23209 for BSA standard ampules) according to the manufacturer’s instructions. Protein lysates were diluted with RIPA lysis buffer as needed, and 6 × Laemmli-buffer (Boston Bioproducts, Cat. No. BP-111R) was added at a final concentration of 1×. After boiling at 95 °C for 5 min, the proteins were separated using 4–20% Mini-PROTEAN TGX Stain-Free Protein Gels and the PVDF membrane Trans-Blot Turbo Transfer System (Bio-Rad Laboratories, Cat. No. 1704159). Membranes were blocked with 5% bovine serum album (BSA) in TBS-0.05% Tween20 for 1 h at room temperature or overnight at 4 °C. Subsequently, blots were incubated overnight at 4 °C with the following primary antibodies: anti-claudin-5 (Abcam, Cat. No. ab1312590, anti-occludin (Thermofisher, Cat. No. 71–1500), anti-ZO-1 (ZO1-1A12) (Thermofisher, Cat. No. 33–9100), anti-connexin 43 (Sigma-Aldrich Cat. No. C6219), anti-pannexin 1 (D9M1C) (Cell Signaling, Cat. No. 911370, anti-connexin 37 (Invitrogen, Cat. No. 42–4400), anti-connexin 45 (Thermofisher, Cat. No. 41–5800), anti-phospho-connexin 43 (Cell Signaling, Cat. No. 3511), and anti-connexin 40 (Thermofisher, Cat. No. 36–4900). All antibodies were used at 1:500 in 5% BSA-TBS. After washing with TBS-0.05% Tween20 three times for 10 min, samples were incubated for 1 h with the secondary antibodies (LI-COR, Lincoln, NE, USA, Cat. No. 926–32210, Cat. No. 926–68070, and Cat. No. 926–32211, Cat. No. 926–68071) at 1:1000 in 5% BSA-TBS and analyzed using the Licor CLX imaging system and the Image Studio 4.0 software (LI-COR). Anti-GAPDH antibody (1:20000, Novus Biologicals, Cat. No. NB600-502IR) was used for protein normalization. The Precision Plus Protein Kaleidoscope Prestained Standard (Bio-Rad, Cat. No. 161–0375) and Precision Plus Protein Dual Color Standard (Bio-Rad, Cat. No. 1610374) were used as molecular weight markers.

### Quantitative PCR

mRNA from pericytes and endothelial cells was extracted using the RNeasy mini kit (Qiagen, Cat. No. 74104) according to the manufacturer’s instructions. Total RNA was quantified using the Nanodrop 2000 (Thermo Fisher Scientific). RT-qPCR was performed using the Applied Biosystems 7500 system (Applied Biosystems). RT-qPCR was performed with 100 ng of RNA per each reaction using the qScript XLT 1-Step RT-qPCR Tough Mix (Quantabio, Cat. No. 89236-676). The following primers were used for gene amplification by TaqMan Gene Expression Assays: Hs00748445_s1 (GJA1), Hs00704917_s1 (GJA4), Hs00271416_s1 (GJC1), Hs00209791_m1 (PANX1), and Hs00270952_s1 (GJA5). Specificity of RT-qPCR results was established using melting curve assessment, and gene expression fluctuations were determined by the 2-ΔΔCt method, with Ct as the cycle number at threshold. The results were normalized to GAPDH expression (Thermofisher, Cat. No. 4352339E).

### Gap Junction Mediated Intercellular Communication

Gap junction-mediated intercellular communication between pericytes and endothelial cells was assessed with a dye transfer technique quantified by flow cytometry. Pericytes were loaded with calcein-AM (1 µM for 30 min at 37 °C; Thermo Fisher Cat. No. C1430). After dye loading, pericytes were washed with DPBS, trypsinized, and added onto a monolayer of endothelial cells. Carbenoxolone (100 μM; Tocris Bioscience Cat No. 3096), a non-specific gap junction blocker, was added to ensure the dye transfer from calcein-loaded pericytes to endothelial cells was dependent on GJ channels. Probenecid (1 mM; Thermo Fisher Cat No. P36400), a pannexin-1 channel blocker, was used to ascertain the effect of hemichannels in dye transfer. Pericytes were allowed to attach to endothelial cells for 4 h at 37 °C. They were then washed, trypsinized and resuspended in DPBS containing 1% FBS (FACS buffer). The cell suspension was blocked with 5% FBS in DPBS for 20 min on ice, followed by staining with cell surface markers CD13 (PE/Fire™ 810 anti-human CD13 Antibody; Biolegend Cat. No. 301737) and CD31 (Brilliant Violet 421™ anti-human CD31 Antibody; Biolegend Cat. No. 303124). Antibodies were diluted to 5 µL per 100 µL in cold FACS buffer. Cells were suspended in 100 µL of diluted antibody and kept in the dark at 4 °C for 30 min. Cells were washed twice with cold FACS buffer and added to 5 mL polystyrene round-bottom tubes with cell strainer caps (Corning, Cat. No. 352235), then analyzed for dye transfer by the BD FACS Aria-IIu cell sorter (BD Biosciences).

### ATP Release Assay

To assess hemichannel activity, an ATP release assay was performed using an ATP RealTime-Glo™ Extracellular ATP Assay (Promega Cat. No. GA5011). Probenecid (1 mM; Thermo Fisher Cat. No. P36400), a pannexin-1 channel blocker, was used to ascertain the effect of hemichannels in dye transfer. Briefly, 20 mL of media was added to the substrate to make a 20× working reagent. 5% by volume of the dissolved substrate was added to both the pericyte and endothelial cell media with or without probenecid. After incubation for 3 h, luminescence at all wavelengths was measured using a Spectramax iD3microplate reader (Molecular Devices).

### Meso Scale Discovery Cytokine Quantification

Soluble RANTES (CCL5), IFN-γ, IL-17A, VEGF, and IL-8 were quantified using the Meso Scale Discovery (MSD) platform with a custom-synthesized U-PLEX Immuno-Oncology Group 1 assay kit (MSD; Cat. No. K151AEM-1) according to the manufacturer’s instructions. Briefly, U-PLEX linkers specific for each analyte were coupled to biotinylated capture antibodies and assembled into a multiplex panel, which was added to blocked U-PLEX plates to generate the multi-spot array. Standards were prepared by serial dilution to generate individual standard curves for each analyte. Culture supernatants were thawed on ice and diluted in kit diluent at 1:2 for IFN-γ, IL-8, IL-17A, and VEGF, and 1:100 for RANTES, in accordance with manufacturer recommendations to minimize matrix effects. Samples and standards were incubated on the plate with shaking, followed by washing and incubation with the SULFO-TAG-labeled detection antibody cocktail. After final washes, MSD Read Buffer was added, and plates were immediately read on an MSD electrochemiluminescence instrument. Analyte concentrations were calculated using four-parameter logistic (4-PL) regression, corrected for dilution factors, and reported as pg/mL.

### IL-8 ELISA

IL-8 production in cell culture supernatants was assessed using a human IL-8 ELISA kit (Abcam, Cat. No. ab214030) according to the manufacturer’s instructions. Absorbance was determined with a Spectramax iD3 microplate reader (Molecular Devices) at 450 nm.

### Statistical Analysis

Statistical analysis was performed by two-way ANOVA to assess the effects of time and infection using GraphPad Prism software (version 10.3.0). Planned post hoc comparisons using Sidak’s multiple comparisons test were restricted to infection effects within each time point on graphs with 3DPI and 7DPI (days are separated by vertical line); no other pairwise comparisons were performed. Two-way ANOVAs were also performed to assess the effects of infection and drug treatment, followed by Tukey’s multiple comparisons test. Values of *p* < 0.05 were considered statistically significant. All data are presented with points of individual experiments and as the mean ± SD.

## Results

### HIV-1 Infected BBB Pericytes Exhibit Active and Latent Infection

The endothelial/pericyte co-culture model was established by seeding primary human brain vascular pericytes and brain microvascular endothelial cells on either side of a transwell in a co-culture system (Fig. 1A) as previously described by our lab [31]. Within this model, pericytes were infected with the CXCR4-tropic (X4) NL4-3 strain for 24 h, followed by removal of the virus and washing the cells from any residual viral particles. Viral replication was assessed by measuring p24 levels for 7 days following infection. The results indicated a peak of p24 levels at 3 days post-infection (DPI), followed by a significant reduction on days 4–7 post-infection (Fig. 1B), suggesting a conversion of active infection at 3DPI to a latent infection at 7DPI. This notion was further assessed by RNAscope for viral RNA (vRNA) and viral DNA (vDNA) after 3 and 7 days of infection (Fig. 1C–F). Non-infected (NI) pericytes were used as a control and expressed no p24 antigens, vRNA, or vDNA (Fig. 1B, C, and E). Infected pericytes expressed cytoplasmic vRNA and vDNA at 3DPI (Fig. 1D). In contrast, at 7DPI, infected pericytes expressed no vRNA, very little cytoplasmic vDNA, and increased nucleic vDNA (Fig. 1F). At 3DPI, infected pericytes expressed significantly higher levels of HIV-1 Gag RNA as quantified by RT-qPCR compared to NI controls (Fig. 1G). In contrast, at 7DPI, HIV-1 Gag expression was not significantly different from NI controls (Fig. 1G). Indeed, 3-day infected cells expressed about 13,000-fold more vRNA than their non-infected counterparts. However, HIV-1 DNA copy number assessed by qPCR showed detectable HIV-1 DNA copy numbers at both time points compared to NI controls (Fig. 1H). We then confirmed the presence of integrated HIV-1 proviral DNA using a two-step Alu-gag PCR assay (Fig. 1I). The first round of PCR amplified the region between the HIV-1 gag gene and the nearest Alu repeat element of the pericyte genome, while the nested PCR amplified the 5′ LTR region of HIV-1 using the first-round amplicon as the template. Figure 1J shows the successful amplification of a 130-base-pair fragment of the 5′-LTR in the HIV-1 genome. The experiment was run using DNA from 3DPI (lanes 3–5) and 7DPI (lanes 6–8). The multiple-aligned sequences generated using the forward primers are shown in Fig. 1K (the reverse complement of the reverse primer sequence is highlighted in yellow), and the multiple-aligned sequences of the reverse complement of the sequences generated using the reverse primers are shown in Fig. 1L (the forward primer sequence is highlighted in yellow). The sequence alignment of the experimental sequencing data with the reference sequence of the LTR-gag region of the HIV-1 strain used for the infection confirms the chromosomal integration of the HIV-1 provirus in pericytes.

These in vitro experiments were next confirmed on brain samples of PLWH. Specifically, brain microvessels of diameter ≤ 10 µm, which correspond to capillaries forming the BBB, were isolated from the temporal cortex of an HIV-seronegative control and an HIV-positive individual on long-term antiretroviral therapy with undetectable plasma viral loads. These microvessels were subjected to RNA scope analysis for DAPI, HIV DNA, HIV RNA, CD31, and PDGFRβ (Fig. 1M). Endothelial cells and pericytes within the microvessels were distinguished using probes for CD31 and PDGFRβ, well-established markers for BBB endothelial cells and pericytes, respectively [36, 37]. Notably, analysis of the HIV +/ART suppressed individual revealed HIV DNA signals that colocalize with BBB pericyte nuclei (Fig. 1M, right panel), while no expression of HIV RNA and HIV DNA was detected in the uninfected individual (Fig. 1M, left panel).

### Active and Latent HIV-1 Pericyte Infection Leads to Endothelial/Pericyte Barrier Dysregulation

**Fig. 2:**
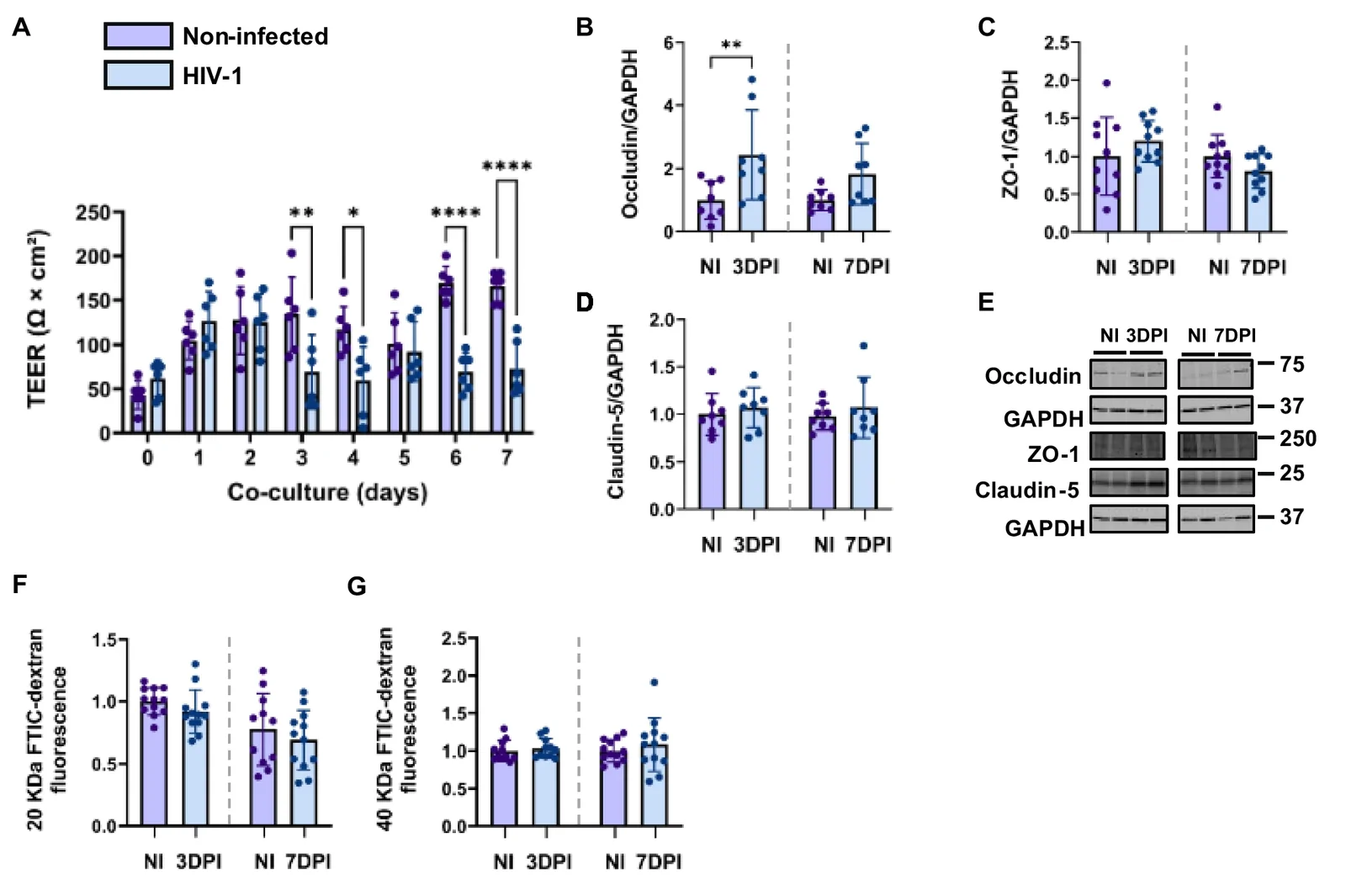
Active and latent infection of BBB pericytes causes endothelial/pericyte barrier impairment. **A** Decreased resistance of the endothelial/pericyte barrier, as measured by TEER, starting 3 days post-infection of BBB pericytes in the co-culture model. Graphs indicate the mean ± SD from 6 independent experiments. **B**, **C**, and (**D**) Expression of tight junction proteins occludin, ZO-1, and claudin-5 in endothelial cells co-cultured with HIV-infected BBB pericytes. Graphs indicate the mean ± SD from 8 to 10 independent experiments. **E** Representative western blot images; molecular weight markers are indicated on the right side. GAPDH was used as a loading control for all proteins. ZO-1 and Claudin-5 were analyzed on the same blot and normalized to the same GAPDH. **F** and (**G**) Integrity of endothelial/pericyte barrier as assessed using 20 kDa or 40 kDa fluorescein isothiocyanate (FITC)-dextran (0.5 mg/mL). Graphs indicate the mean ± SD from 11 to 12 independent experiments. *p < 0.05, **p < 0.01, ****p < 0.0001

Compromised BBB integrity and function has been linked to the progression of NCI in PLWH [28–30, 38]. Since pericytes are in direct contact with endothelial cells, they play a crucial role in the regulation of the BBB. To assess the impact of pericyte infection on barrier function, we measured transendothelial electrical resistance (TEER) daily over a 7-day infection period in the co-culture model with NI endothelial cells. We noted a significant decrease in TEER values at days 3, 4, 6, and 7DPI as compared to non-infected co-cultures (Fig. 2A), suggesting disruption of paracellular permeability by both actively and latently infected pericytes. We then evaluated transcellular permeability of the endothelial/pericyte barrier by testing its solute permeability to two different-sized FITC-dextran molecules (20 kDa and 40 kDa) (Fig. 2F and G). To differentiate between active and latent pericyte infection, we used the same 3- and 7-day time points that were validated in Fig. 1, and the endothelial cells remained non-infected. We observed no significant changes in permeability with either size of the FITC-dextran molecules (Fig. 2F and G), suggesting no transcellular disruption. Furthermore, we examined the expression of tight junction proteins in the endothelial cells and found a significant increase in occludin expression after 3 days of co-culture with infected pericytes, with a continued upward trend after 7 days (Fig. 2B). No significant changes in zonula occludens-1 (ZO-1) and claudin-5 expression were noted (Fig. 2C and D).

### Active and Latent HIV-1 Pericyte Infection Causes Differential Changes in GJ and HC Expression

We next focused on examining whether BBB pericyte infection affects GJ- and HC-mediated communication with non-infected endothelial cells. First, we evaluated the expression of key GJ and HC genes and proteins in both pericytes and endothelial cells. Connexin 43 (Cx43) is the predominant connexin expressed in both pericytes and endothelial cells, and communication through Cx43 GJ channels is essential for vascular homeostasis [38]. Additionally, connexin 37 (Cx37) and connexin 45 (Cx45) are expressed in both cell types, forming GJ channels vital for pericyte-endothelial cell communication [39–44]. Connexin 40 is also widely expressed in BBB endothelial cells, but less so in BBB pericytes [39]. Both pericytes and endothelial cells also express uHCs, which are formed by either connexin 43 or pannexins, primarily pannexin 1 (Panx1).

**Fig. 3:**
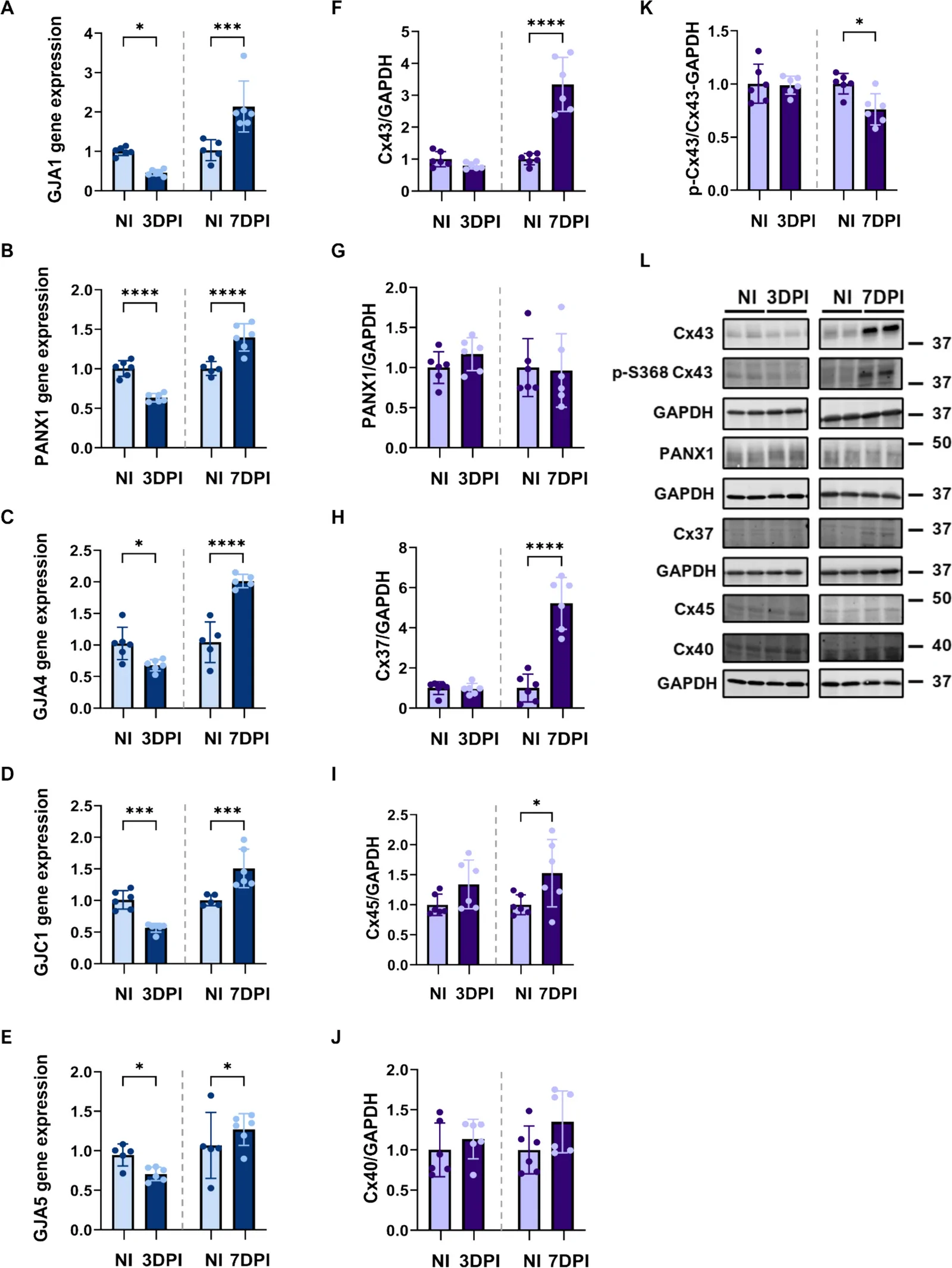
Dysregulation of gap junction and hemichannel expression in HIV-1-infected BBB pericytes. Decreased expression after 3 days of pericyte infection and increased expression after 7 days of gap junction (GJ) genes: **A** GJA1, **B** Panx1 (pannexin 1), **C** GJA4, **D** GJC1, and (**E**) GJA5. Gene expression was measured with RT-qPCR. Expression of connexin and pannexin proteins in pericytes following 3 or 7 days of infection measured by Western blotting: **F** Cx43, **G** Panx1, **H** Cx37, **I** Cx45, **J** Cx40, and (**K**) phospho-Cx43 at serine 368. **L** Representative images of the western blots of GJ proteins; molecular weight markers are indicated on the right side. GAPDH was used as a loading control for all proteins. Cx43 and phospho-Cx43 were analyzed on the same blot and normalized to the same GAPDH. Cx45 and Cx40 were normalized to the same GAPDH. Graphs indicate the mean ± SD from 5 to 6 independent experiments. *p < 0.05, ***p < 0.001, ****p < 0.0001

After infecting pericytes for 3 or 7 days in co-culture with endothelial cells, we assessed the mRNA expression of *GJA1, Panx1, GJA4, GJC1, and GJA5* by RT-qPCR using mRNA isolated exclusively from pericytes. All five genes were significantly downregulated at 3DPI compared to NI controls (Fig. 3A, B, C, D, and E). Conversely, a significant upregulation in these genes was found at 7DPI (Fig. 3A, B, C, D, and E), i.e., in latently infected pericytes. Moreover, we assessed the expression of the corresponding proteins Cx43, Panx1, Cx37, Cx45, and Cx40. At 7 DPI, there was a significant increase in Cx43 protein expression as compared to its NI control (Fig. 3F). Similarly, we observed a significant increase in Cx37 and Cx45 protein expression at 7DPI compared to NI controls (Fig. 3H and I). No significant change in pannexin 1 protein expression (Fig. 3G) or Cx40 protein expression (Fig. 3J) was found in infected pericytes. Phosphorylation of connexins and pannexins plays a crucial role in regulating various stages of the gap junction life cycle, influencing their assembly, disassembly, and degradation [40]. Due to the importance and significant upregulation of Cx43, we evaluated phosphorylation of serine 368 on the carboxyl terminal tail of Cx43 [41]. There was a significant decrease in phosphorylation of Cx43 at serine 368 as compared to total Cx43 levels in latently infected pericytes at 7DPI (Fig. 3K).

**Fig. 4:**
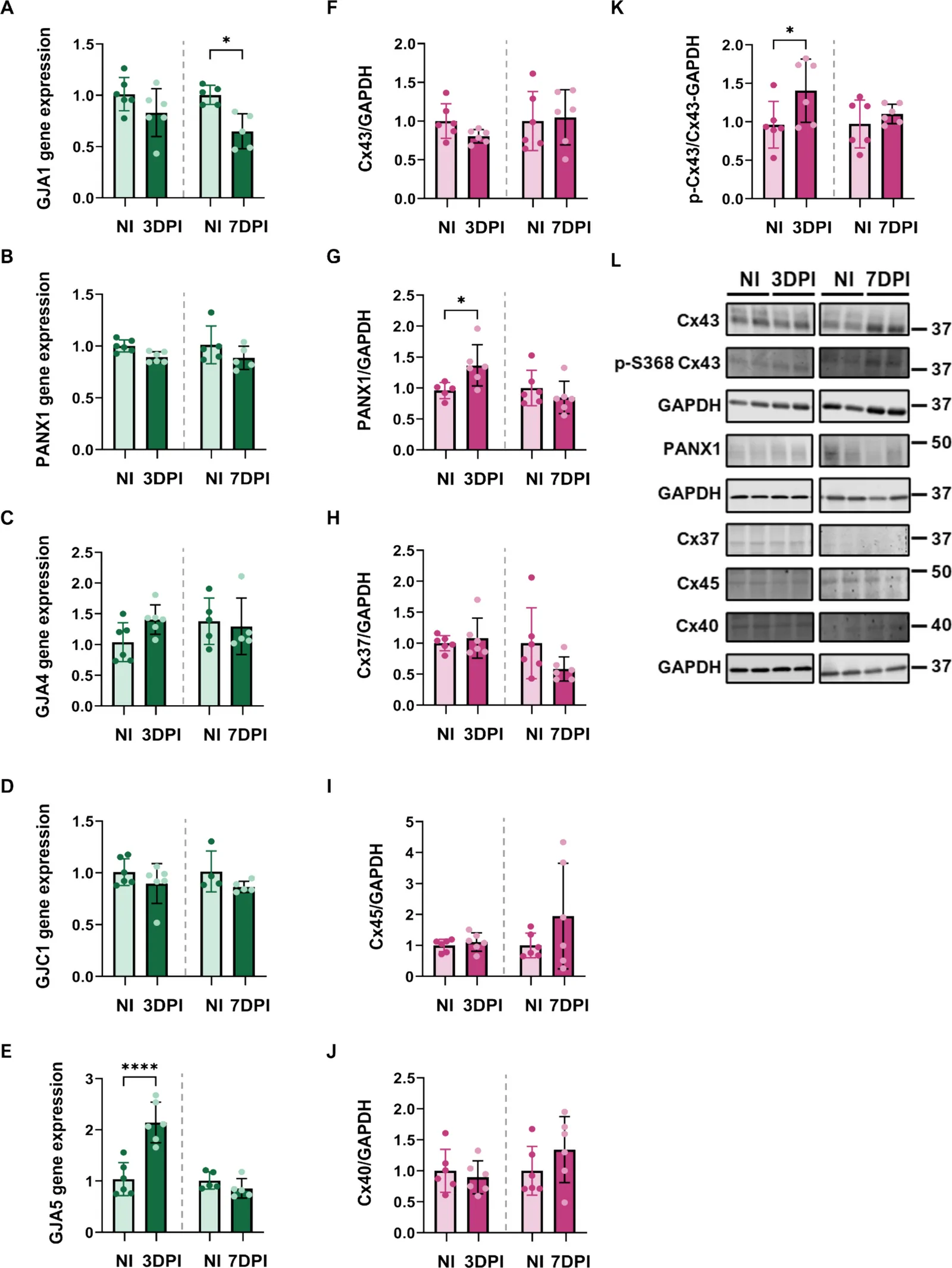
Differential regulation of gap junction and hemichannel expression in brain microvascular endothelial cells co-cultured with infected BBB pericytes. Expression of gap junction genes (**A**) GJA1, **B** Panx1, **C** GJA4, and (**D**) GJC1, and (**E**) GJA5 in brain endothelial cells following 3- or 7-days of BBB pericyte infection. Corresponding connexin and pannexin protein expression showed some differential regulation: **F** Cx43, **G** Panx1, **H** Cx37, **I** Cx45, and (**J**) phospho-Cx43 at serine 368. Gene expression was measured with RT-qPCR and protein expression was measured with western blotting. **L** Representative images of the western blots of GJ proteins; molecular weight markers are indicated on the right side. GAPDH was used as a loading control for all proteins. Cx43 and phospho-Cx43 were analyzed on the same blot; therefore, they were normalized to the same GAPDH. Cx37, Cx45, and Cx40 were analyzed on the same blot and normalized to the same GAPDH. Graphs indicate the mean ± SD from 5 to 6 independent experiments. *p < 0.05, ****p < 0.0001

We then assessed the expression of the same GJ and HC genes in NI endothelial cells co-cultured with infected pericytes, as effective GJ-mediated intercellular communication requires each cell to contribute a connexon, composed of six connexins. *GJA1* expression in endothelial cells decreased following 7 days of pericyte infection (Fig. 4A), while the expression of *GJA5* increased at 3DPI (Fig. 4E). There were no other significant changes in connexin or pannexin gene expression (Fig. B, C, and D). However, Panx1 protein expression in NI endothelial cells was differentially regulated by pericyte infection, showing a significant increase at 3DPI compared to controls (Fig. 4G). No significant changes in Cx43, Cx37, Cx45, or Cx40 protein expression were observed in NI endothelial cells (Fig. 4F, H, I, and J). Interestingly, there was an increase in phosphorylation of Cx43 at serine 368 in endothelial cells relative to total Cx43 levels after 3 days of pericyte infection compared to non-infected controls, with no significant changes observed at 7DPI (Fig. 4K).

### HIV-1 Infection Increases GJ- and HC-Mediated Communication Between Pericytes and Endothelial Cells

A dye transfer assay was employed to evaluate functional gap junction communication between pericytes and endothelial cells. After 3 or 7 days of pericyte infection in monoculture, pericytes were stained with calcein acetoxymethyl ester (Calcein-AM), which fluoresces upon hydrolysis to calcein inside the cells and subsequently transfers out through gap junctions. Calcein-loaded pericytes were trypsinized and plated onto an unstained monolayer of endothelial cells and co-cultured for 4 h to allow for dye transfer from the pericytes to the endothelial cells. During the 4-h incubation, carbenoxolone (CBX), a general connexin inhibitor, was added to confirm that the observed effects were GJ-related. We also assessed the contribution of pannexin-1 hemichannels in the dye transfer by employing probenecid, a pannexin 1 blocker. To assess the dye transfer between cell types, we stained the combined cells with CD31 and CD13 cell surface markers to distinguish between pericytes (CD13 positive) and endothelial cells (CD13 and CD31 double positive) (Supp. Figure  1 A and B). Flow cytometry was used to quantify the amount of dye transfer from infected or non-infected pericytes to endothelial cells.

**Fig. 5:**
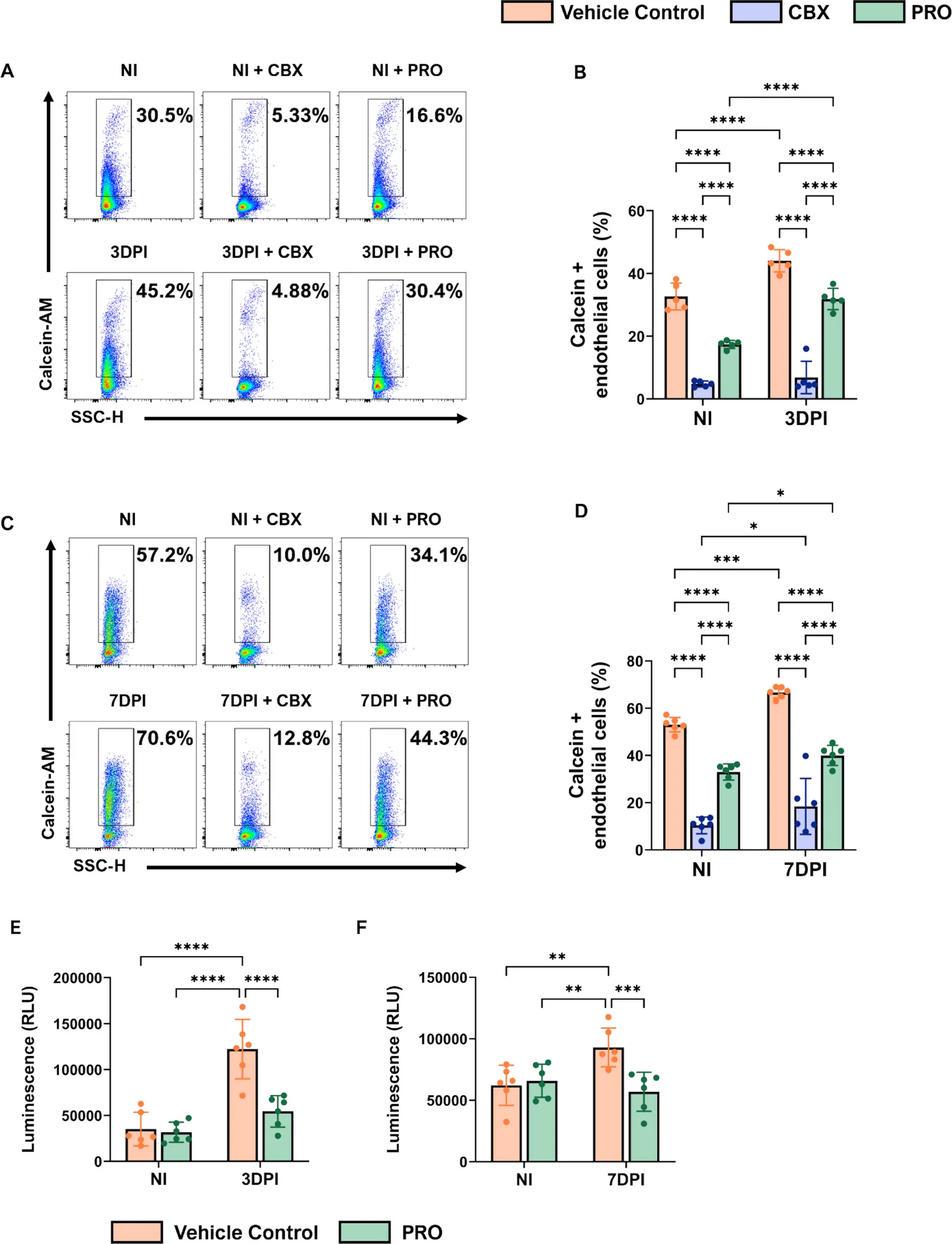
HIV-1 infection of BBB pericyte increases GJ and HC-mediated communication to endothelial cells. **A** Representative flow cytometry images of calcein dye transfer from pericytes into endothelial cells after 3 days of pericyte infection. The graphs shown are the endothelial cell population (CD31 and CD13 double positive) with gating indicating the percentage of cells positive for calcein dye. The percentage of endothelial cells positive for calcein is displayed in the upper right quadrant of each graph. Pericytes were either non-infected (NI) or infected (3DPI) and treated with vehicle control, carbenoxolone (CBX), or probenecid (PRO). One representative image of each group is shown in (**A**), and quantification is shown in (**B**). **C** Representative flow cytometry images of calcein dye transfer from latently infected pericytes (7DPI) to endothelial cells with quantification shown in **D**. Extracellular ATP from pericyte/endothelial cell co-culture media was assessed after pericyte infection at (**E**) 3DPI and (**F**) 7DPI with non-infected (NI) cultures serving as controls. PRO was added to selectively inhibit Panx1 hemichannels. Vehicle controls were treated with cell culture-grade water. Graphs indicate the mean ± SD from 6 independent experiments. *p < 0.05, **p < 0.01, ***p < 0.001, ****p < 0.0001

To quantify GJ-mediated dye transfer from infected pericytes to endothelial cells, we measured the percent of calcein-positive endothelial cells (double-positive for CD13 and CD31). We observed that 30.5% of endothelial cells were calcein-positive when cultured with 3-day NI pericytes. Notably, co-culture with infected pericytes at 3DPI showed a significant increase in dye transfer with 45.2% calcein-positive endothelial cells (Fig. 5A and B). The addition of CBX to both groups significantly decreased calcein transfer into endothelial cells, confirming that the majority of dye transfer occurred through GJs (Fig. 5A and B). Furthermore, PRO treatment decreased dye transfer levels in both the non-infected and HIV-1 groups, indicating that some intercellular communication in our co-culture models can be mediated via Panx-1 HCs (Fig. 5A and B). Interestingly, latent pericyte infection also significantly enhanced GJ-mediated dye transfer to endothelial cells by about 13% compared to NI controls (Fig. 5C and D). This effect was also significantly attenuated by CBX and PRO (Fig. 5C and D).

Panx1 and Cx-containing uHCs play a significant role in paracrine and autocrine cell-to-cell communication. To assess HC activity, we measured the levels of intracellular ATP secreted into the cell culture media via HCs in co-cultures of infected pericytes and NI endothelial cells. HIV-1 infection resulted in a significant increase of ATP release at 3DPI (Fig. 5E) and continued to significantly elevate ATP release at 7DPI (Fig. 5F). A co-treatment of HIV-1-infected pericytes with PRO restored ATP release to the control levels observed in NI pericytes, confirming that HCs are responsible for the elevated ATP release in the infected samples (Fig. 5E and F).

### Blocking GJ Communication from HIV‑1-Infected Pericytes Modulates Endothelial Cytokine Release

HIV-1 infection promotes a heightened inflammatory environment, contributing to the compromise of BBB integrity [42, 43]. Importantly, HIV-1 infection is known to enhance cytokine release [44]. To determine if enhanced GJ signaling from HIV-infected pericytes affects the secretion of inflammatory mediators within the endothelial-pericyte co-culture system and consequently the barrier integrity, GJs were pharmacologically blocked in endothelial cells/pericyte co-culture in both cell types by adding CBX for 4 h, followed by media collection. CBX was then washed off, and 20 h later, more media samples were collected to assess the levels of IFN-γ, IL-8, IL-17, VEGF, and RANTES in the endothelial cell culture media using the Meso Scale Discovery (MSD) platform.

**Fig. 6:**
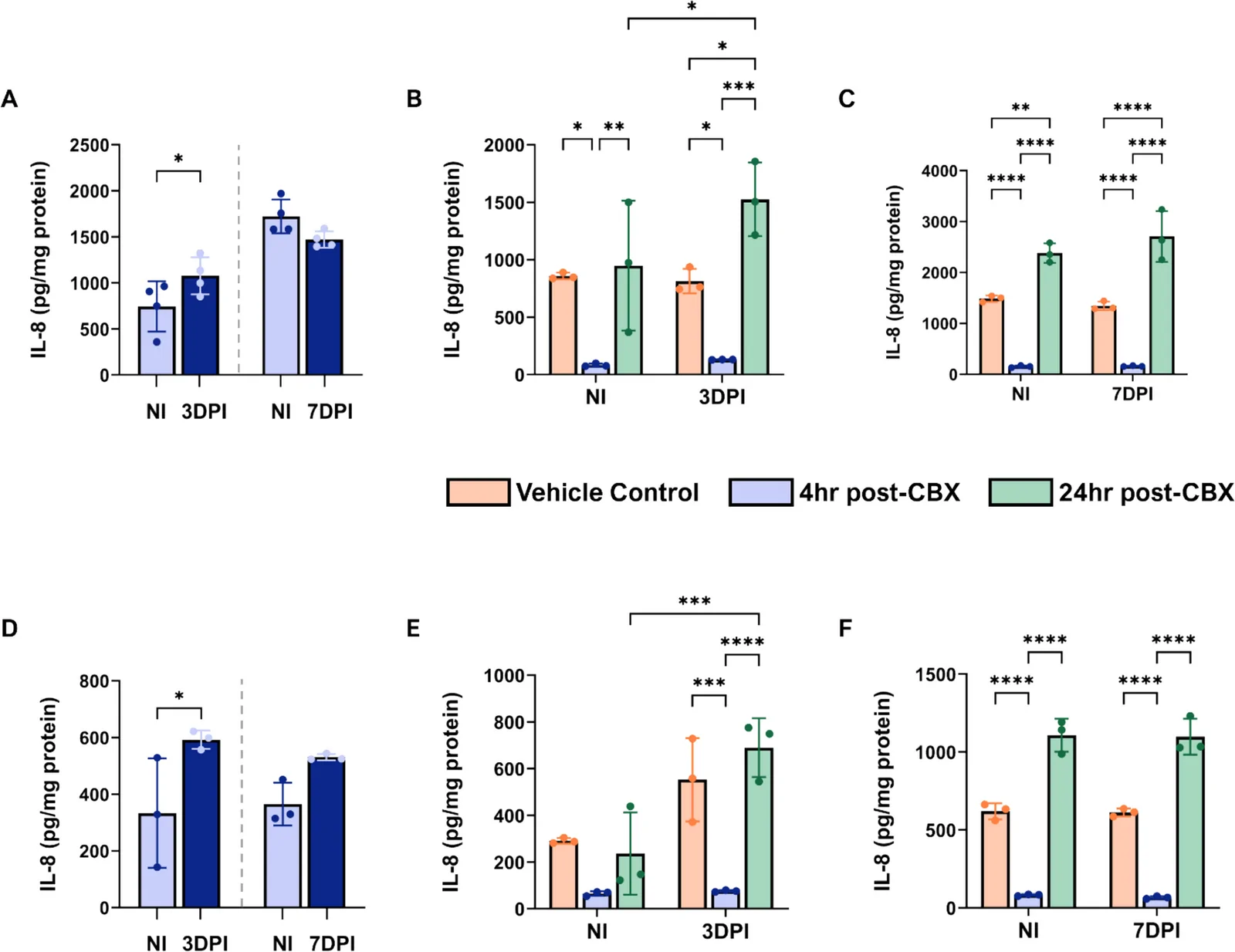
Inhibition of GJs affects secretion of IL-8 from endothelial cells in co-culture with HIV-1 infected pericytes. IL-8 levels measured using the Meso Scale Discovery (MSD) platform (**A**, **B**, and **C**) and via individual IL-8 ELISA using a microplate reader (**D**, **E**, and **F**). IL-8 levels in media collected from the endothelial cell side of co-cultures at 3DPI and 7DPI (**A**, **D**). IL-8 levels in media collected before treatment, 4 h after CBX treatment (100 µM), and 24 h after CBX treatment at 3DPI (**B**, **E**) and 7DPI (**C**, **F**). Pretreatment and 4 h post-CBX samples were collected at 2DPI and 6DPI, respectively. Vehicle controls were treated with an equivalent volume of cell culture-grade water. Graphs indicate the mean ± SD from 3–4 independent experiments. *p < 0.05, **p < 0.01, ***p < 0.001, ****p < 0.0001

While the levels of IFN-γ, IL-17, VEGF, and RANTES were below the range of detection under our culture conditions, significant changes were observed in the levels of pro-inflammatory cytokine IL-8 (Fig. 6A–C). Specifically, the levels of IL-8 markedly increased at 3DPI compared to NI controls with no changes at 7DPI (Fig. 6A). Inhibition of GJs with CBX resulted in a significantly decreased release of IL-8 at 3DPI, 7DPI, and in both NI controls (Fig. 6B and C). Notably, 24 h post-CBX treatment, the levels of IL-8 secreted were significantly higher at 3DPI than in NI controls (Fig. 6B) with no significant changes at 7DPI (Fig. 6C).

To confirm these findings, IL-8 levels in the endothelial media were also measured via ELISA. IL-8 levels were significantly increased at 3DPI compared to NI controls with no changes observed at 7DPI (Fig. 6D). CBX had a greater effect on decreasing IL-8 secretion 4 h post CBX at 3DPI compared to its NI control (Fig. 6E). Additionally, IL-8 levels secreted 24 h post CBX treatment were higher at 3DPI than in NI controls (Fig. 6E) with no changes 24 h post CBX at 7DPI (Fig. 6F). These results are consistent with the initial MSD results, confirming that blocking GJs increased inflammatory signaling of IL-8 from the co-culture system of endothelial cells and HIV-infected pericytes.

## Discussion

HIV-associated neurocognitive impairment (NCI) remains a global health problem due to the establishment of viral reservoirs in the CNS and the resulting inflammation [4]. Although the advent of ART has enabled PLWH to survive longer, premature neurocognitive decline persists due to structural and functional changes in the brain [45]. Within the CNS, microglia, astrocytes, and pericytes are all potential cellular targets for HIV-1, contributing to viral latency in the brain [6–11]. Recent studies highlight the potential of pericytes as a key latent viral reservoir of the CNS [6, 12, 13]. Pericytes have a pivotal role at the BBB, covering about 90% of the brain endothelium, facilitating direct signaling to neighboring cells, BBB formation, and maintenance. GJ- and HC-mediated intercellular communication between pericytes and endothelial cells is essential for BBB integrity [17, 18]. The results of the present study indicate that HIV-1 infection of pericytes disrupts this intercellular signaling, potentially compromising BBB integrity, which may then contribute to neurocognitive impairment. This disruption of pericyte-endothelial intercellular communication represents a previously unrecognized mechanism by which HIV-1 may accelerate neurovascular dysfunction.

To model the pericyte/endothelial cell interactions, an in vitro co-culture model of primary human brain pericytes and brain microvascular endothelial cells was established (Fig. 1A). Analysis of this model confirmed that HIV-1-infected pericytes exhibit both active and latent phenotypes, allowing us to investigate the potential impact of latent pericyte infection on the function of NVUs. Specifically, p24 antigen production peaked at 3DPI, indicating an active phenotype, followed by a significant decrease from days 4–7, indicating a transition to latency (Fig. 1B) and supporting previous studies [6, 12]. These results were confirmed by RNAscope, which showed pericytes express high levels of cytoplasmic vRNA at 3DPI (Fig. 1D) and high levels of nuclear vDNA with no detectable vRNA at 7DPI (Fig. 1F). While qPCR analysis of HIV-1 DNA revealed higher HIV-1 copy numbers at 3DPI than at 7DPI (Fig. 1H), the method did not distinguish between integrated and unintegrated DNA. Therefore, HIV-1 DNA levels at 3DPI likely reflect the presence of unintegrated DNA, as observed in RNAscope imaging. At day 7, HIV-1 DNA levels were lower, suggesting that we are primarily detecting integrated viral DNA in the cell nuclei, similarly observed in the RNAscope imaging. Next, we used an established method of Alu-Gag PCR and Sanger sequencing to confirm chromosomal integration of the HIV-1 provirus in pericytes [35] (Fig. 1I-L). Altogether, these results confirm that BBB pericytes can be latently and actively infected in vitro [6, 12, 13]. Moreover, HIV DNA was detected in human temporal cortex microvessels from an HIV-positive individual (Fig. 1M), indicating viral persistence and latency within the neurovascular unit despite systemic viral suppression.

Studies have shown that BBB disruption, even in individuals on ART, is a hallmark of HIV-1 infection [28, 46], leading to microvascular and neurovascular degeneration and contributing to neurocognitive decline. Indeed, the results from this study confirmed endothelial dysregulation in a co-culture model with HIV-infected pericytes. TEER values significantly decreased during active pericyte infection and persisted into latency, indicating affected paracellular integrity of the endothelial cell monolayer (Fig. 2A). Gap junction-mediated signaling plays an important role in coordinating endothelial barrier homeostasis; therefore, dysregulation of gap junction communication may contribute to impairment of endothelial junction integrity and reductions in TEER [47]. Consistent with this, the reduction in TEER was accompanied by increased gap junction communication between infected pericytes and NI endothelial cells (Fig. 5), suggesting that enhanced intercellular coupling may facilitate the transfer of pathogenic signals from infected pericytes to endothelial cells, thereby contributing to compromised endothelial barrier integrity.

On the other hand, we did not observe any differences in the passage of 20 kDa or 40 kDa FITC-dextran through the co-culture, suggesting that transcellular permeability is not affected (Fig. 2F and G). Indeed, endothelial expression of claudin-5, the tight junction protein primarily responsible for the regulation of barrier function [48], was not affected in our model system (Fig. 2D). Furthermore, the expression of ZO-1, a primarily scaffolding protein that regulates the location of transmembrane proteins [49], was not changed (Fig. 2C). Co-cultures of infected pericytes and non-infected endothelial cells dysregulated expression of occludin (Fig. 2B) within the endothelial cells. Occludin increase in endothelial cells could be due to several factors, including inflammation, increased oxidative stress, and viral-induced damage [38, 50]. Specifically, occludin has been shown to influence cellular metabolism and play a role in regulation of HIV-1 infection, including in pericytes [38, 49, 51]. Studies have shown that occludin modulates the expression of the OAS family of interferon-stimulated genes, which function in innate immunity to activate the endoribonuclease RNaseL, providing antiviral protection by viral RNA degradation [52, 53]. Due to these functions of occludin, our increase in occludin at 3DPI suggests a potential protective response. Furthermore, this increase indicates that occludin may be participating in establishing latent infection of pericytes due to its antiviral protective effects [38, 53].

Important findings of this study demonstrate that HIV-1 infection of BBB pericytes leads to dysfunctional GJ-mediated signaling to endothelial cells. During active infection, key gap junction genes in pericytes were downregulated (Fig. 3A–E), potentially due to HIV-associated proteins mediating cellular suppression [54, 55]. However, increased expression of gap junction genes during latent infection of pericytes suggests a novel mechanism for amplifying HIV-1-mediated toxicity and chronic neuroinflammatory responses by brain reservoirs. Selected gap junction proteins Cx43 and Cx37 were also significantly upregulated (Fig. 3F and H), suggesting an increase in the number of GJ channels. Moreover, phosphorylation of Cx43 by protein kinase C at serine 368 alters Cx43 function by inducing conformational changes that promote internalization and degradation to reduce intercellular communication [41]. We observed a significant decrease in Cx43 phosphorylation during latent infection (Fig. 3K), suggesting that gap junctions may remain at the membrane longer since they are not receiving signals for endocytosis and degradation, leading to increased toxicity and inflammation [41].

Interestingly, although endothelial cells are not susceptible to HIV-1 infection, gene expression of gap junctions and hemichannels was significantly altered in co-cultures with both actively and latently infected pericytes. Analysis of endothelial cells revealed selective dysregulation in the expression of the *GJA1* and *GJA5* genes (Fig. 4A and E) as well as the Panx1 protein (Fig. 4G). The increased expression of Panx1 at 3DPI aligns with the activation of hemichannels in response to stimuli or stress signals released by actively infected pericytes [56, 57]. Furthermore, the increased phosphorylation of Cx43 during active infection (Fig. 4K) may reduce gap junction communication by promoting the endocytosis and degradation of Cx43 proteins [41]. Cx43 phosphorylation may represent a compensatory response to enhanced hemichannel activity and/or signaling from actively infected pericytes [58].

Findings of this study reveal enhanced functional gap junction communication between HIV-infected pericytes and endothelial cells at the time of both active and latent infection. Dye transfer to endothelial cells via GJs increased by approximately 12% during active HIV-1 infection (Fig. 5B). This effect persisted during latent HIV-1 infection, with a 13% increase in dye transfer in the infected group compared to controls (Fig. 5D). These results are supported by the significant attenuation of dye transfer after the addition of CBX, a potent general inhibitor of GJ-mediated communication. Moreover, dye transfer was also reduced by about 15–25% after the addition of PRO, a specific Panx1 inhibitor, indicating the involvement of Panx1 HCs in pericyte-endothelial mediated communication during infection. HCs allow signaling between cells via the release of small molecules, including ions, glutamate, aspartate, ATP, and prostaglandins, into the extracellular space [59]. In fact, we observed increased extracellular ATP release from pericyte-endothelial co-cultures during both active and latent HIV-infection (Fig. 5E and F), with a more pronounced effect at the active time point. This further supports the enhanced involvement of HCs following pericyte infection. ATP release into the extracellular space has been shown to have implications in HIV-1 infection via purinergic receptor activation [60]. Additionally, signaling via purinergic receptors plays a critical role in regulating BBB permeability [61]. Extracellular ATP functions as a damage-associated molecular pattern (DAMP) that activates purinergic receptors and triggers downstream inflammatory pathways [62]. Studies have demonstrated that release of IL-1β and subsequent production of MMP-9 via purinergic signaling contributes to tight junction breakdown and BBB damage [62]. Therefore, increased ATP signaling through hemichannels and purinergic receptors likely contributes to endothelial cell dysfunction and BBB breakdown.

Chronic inflammation is prevalent in PLWH due to latent viral reservoirs that sustain low-level immune activation [63]. This elevated inflammatory state has harmful effects in the CNS and contributes to neurocognitive comorbidities [63]. Therefore, we assessed cytokine expression in the endothelial medium of a co-culture with HIV-infected pericytes. We focused on the endothelial side of the co-culture to assess whether GJ-mediated pericyte-endothelial crosstalk alters the local inflammatory environment at the barrier.

Our results indicate that there is an increase in the level of IL-8 in the endothelial co-culture medium at 3DPI (Fig. 6A and D), indicating a system-level inflammatory response to infection, the cellular source of which was not determined in this study. These observations align with the fact that HIV-infected cells and HIV proteins have been shown to induce secretion of IL-8 in human brain microvascular endothelial cells [64–66]. Furthermore, IL-8 levels have been shown to be significantly elevated in cerebrospinal fluid of PLWH that had NCI compared to those without NCI [67, 68].

To build on the findings that HIV-1 infection of pericytes changes the inflammatory profile of co-cultured endothelial cells, we employed CBX to determine if blocking enhanced GJ signaling can impact inflammatory secretion from endothelial cells. Indeed, blocking GJ signaling for 4 h almost eliminated IL-8 secretion in both NI and HIV groups (Fig. 6B, C, E, and F). This effect was less pronounced when measured via IL-8 ELISA in the NI group compared to 3DPI (Fig. 6E). The reduction of IL-8 secretion after CBX treatment suggests that CBX is blocking the intracellular GJ signaling pathways needed to induce an immune response [69, 70]. After removal of CBX, the levels of IL-8 in NI cells return to baseline secretion, whereas IL-8 was significantly elevated at 3DPI compared to NI cells 24 h post-CBX treatment (Fig. 6B and E). No significant changes in IL-8 secretion were noted 24 h post-CBX at 7DPI (Fig. 6C and F). The changes at 3DPI likely reflect reliance of infection on GJ-mediated propagation of injury signals. Removal of CBX can restore GJ-mediated molecular coupling [71] between infected pericytes and endothelial cells, which is consistent with the increased ATP release at 3DPI compared to NI controls (Fig. 5E). Although ATP levels remained elevated at 7DPI compared to NI controls, it was to a lesser extent compared to 3DPI. These findings may be contributing to the changes in IL-8 secretion at 3DPI. These results indicate that HIV can disrupt the ability of pericyte—endothelial GJ signaling to regulate cytokine secretion in a similar matter to GJ signaling in HIV-infected astrocytes [25].

In conclusion, our findings indicate a novel mechanism of HIV-1-induced NVU dysfunction in which HIV-infected pericytes utilize GJ- and HC-mediated communication with endothelial cells, contributing to barrier impairment. Remarkably, altered intercellular communication has been executed not only by actively but also by latently HIV-1-infected pericytes, providing novel insight about the importance of CNS HIV reservoirs. Notably, blocking GJ channels modulates the inflammatory responses of the co-culture, which may contribute to improved BBB integrity. This research enhances our understanding of the intercellular and molecular mechanisms underlying HIV-1 infection at the NVU level, which can be leveraged for the development of new therapeutic targets.

## Supplementary Information

Below is the link to the electronic supplementary material.

Supplementary file1 (PDF 7084 kb)

Supplementary file2 (PDF 318 kb)

## Data Availability

All source data supporting the findings of this manuscript are available from the corresponding authors upon request.
