# Supplementary material for: Latent HIV Infection of BBB Pericytes Enhances Gap Junction-mediated Crosstalk with Endothelial Cells
Source: Mol Neurobiol. 2026 Oct 16;63(1):973. doi: 10.1007/s12035-026-06266-0 (PMC13675307; doi:10.1007/s12035-026-06266-0)

# Uncropped Gels and Blots

## Fig. 2 Occludin (3DPI and 7DPI)

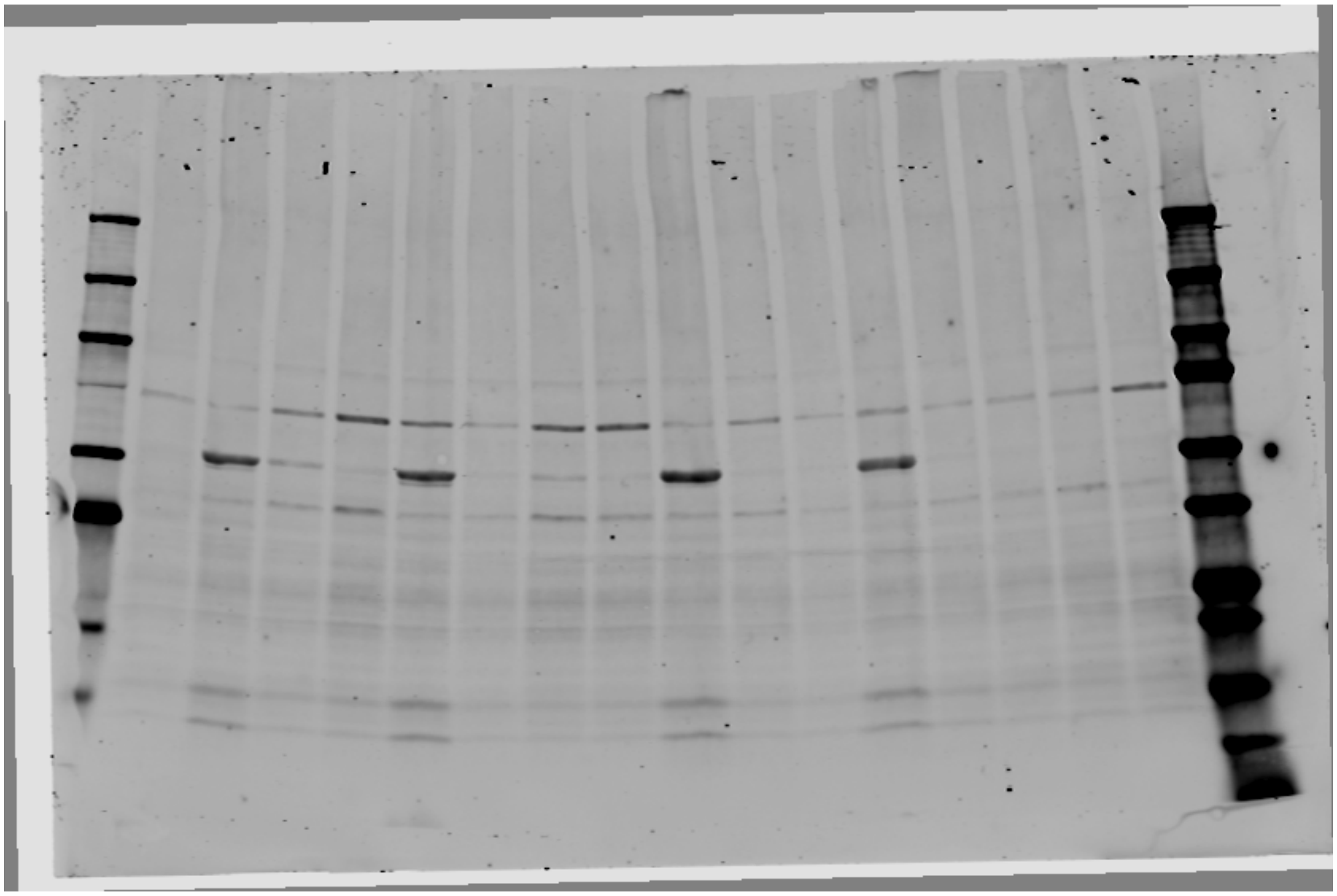

## Fig. 2 GAPDH for Occludin (3DPI and 7DPI)

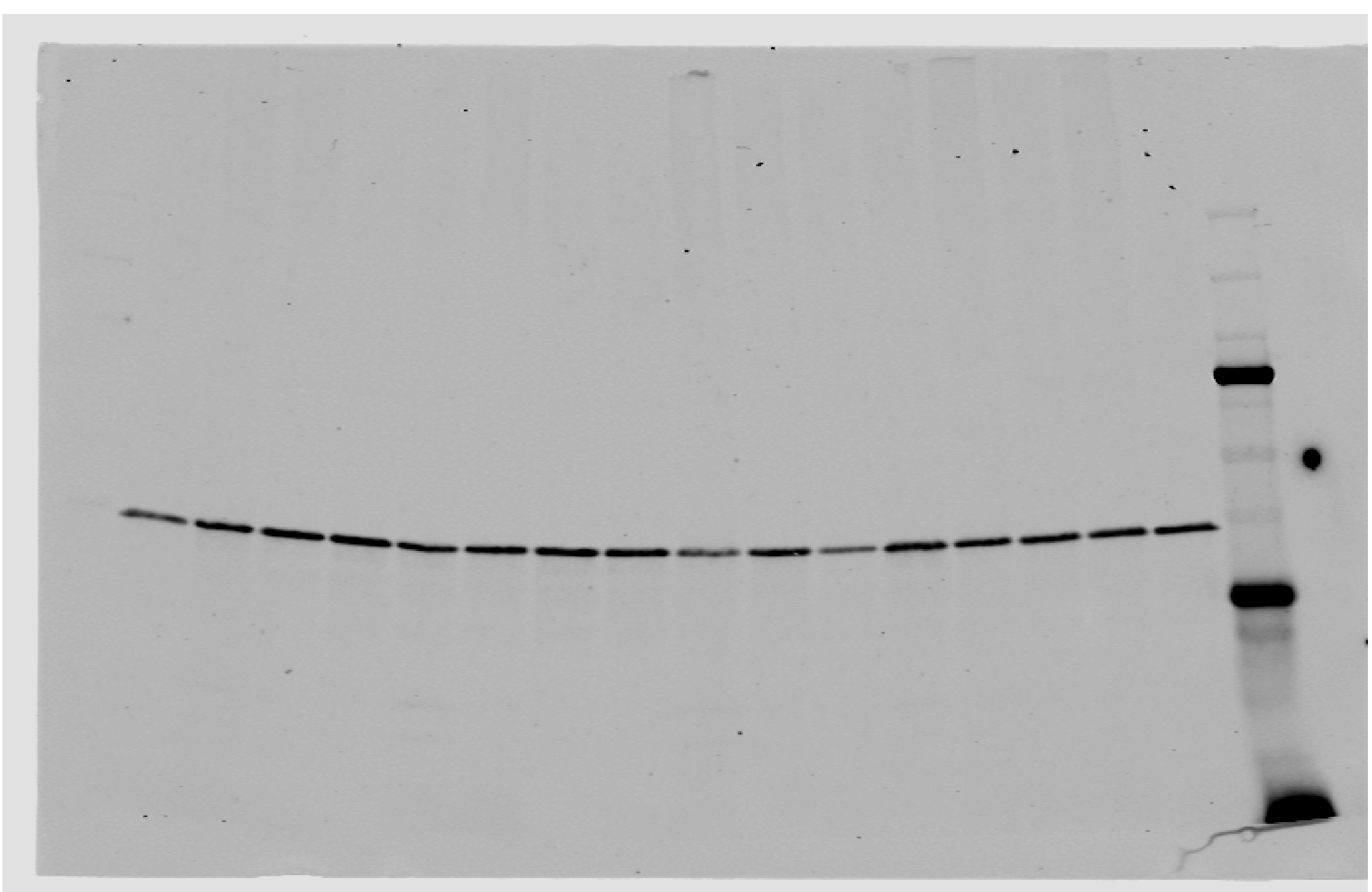

Fig. 2 ZO-1 (3DPI and 7DPI)

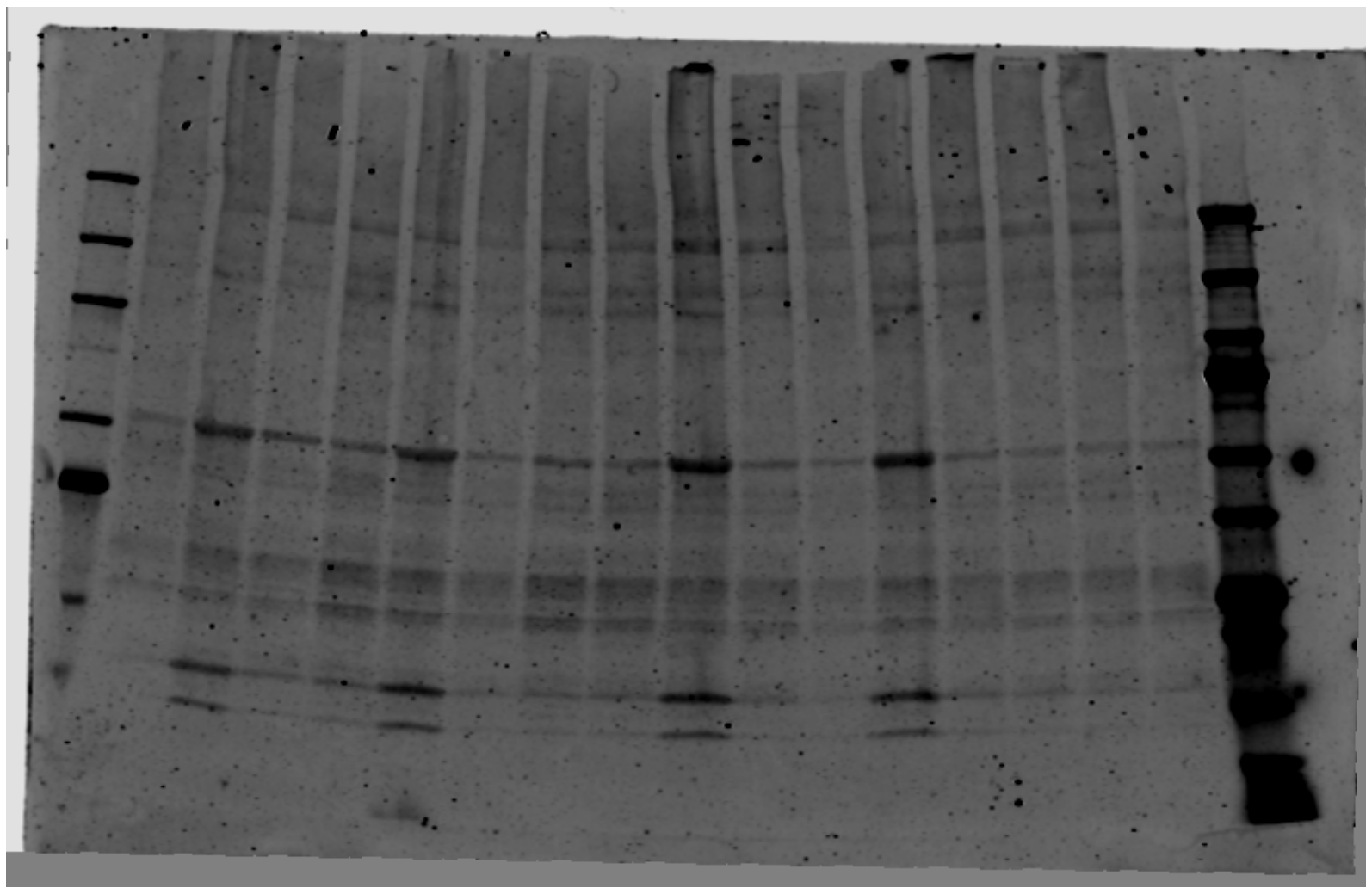

Fig. 2 Claudin-5 (3DPI and 7DPI)

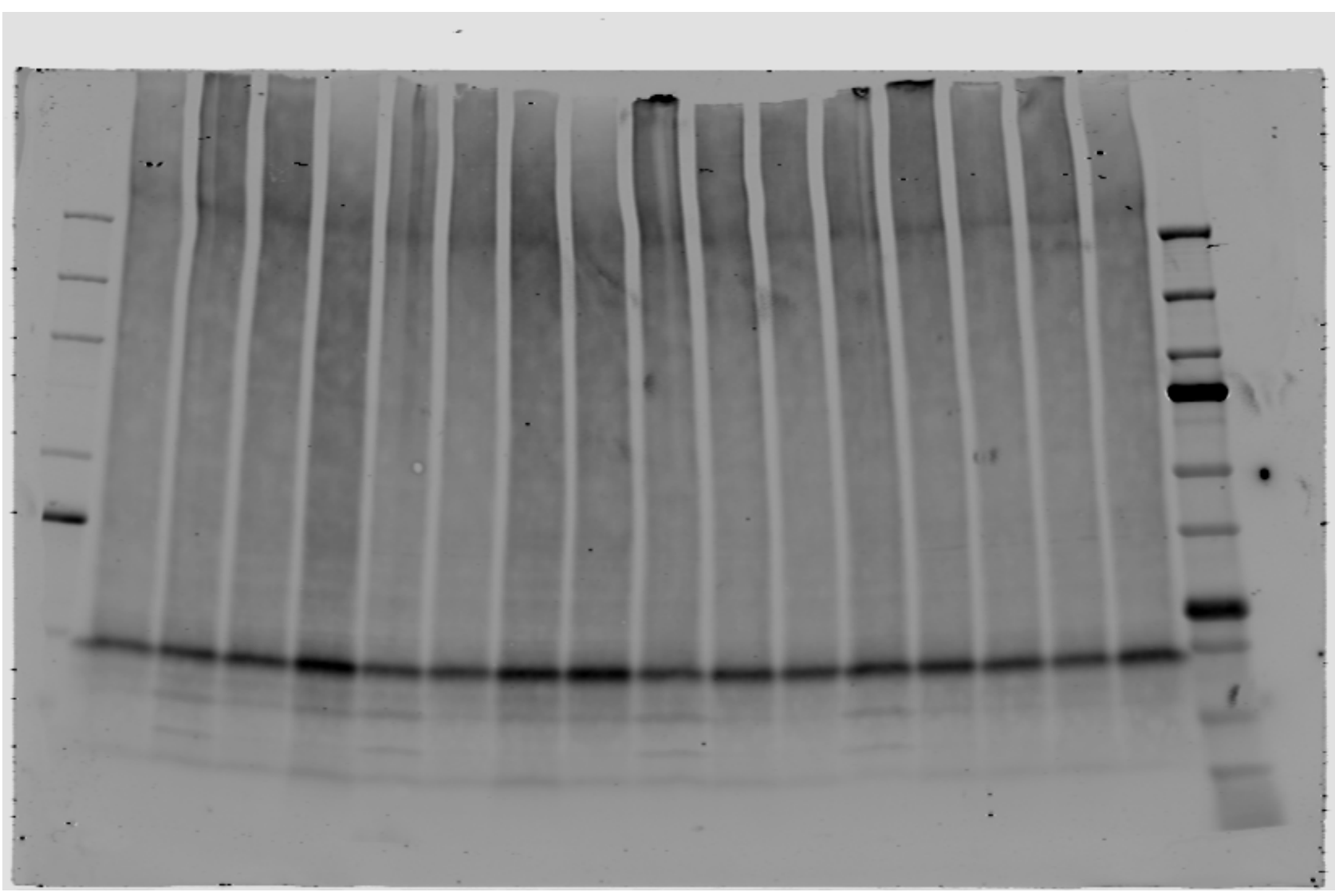

Fig. 2 GAPDH for ZO-1 and Claudin-5 (3DPI and 7DPI)

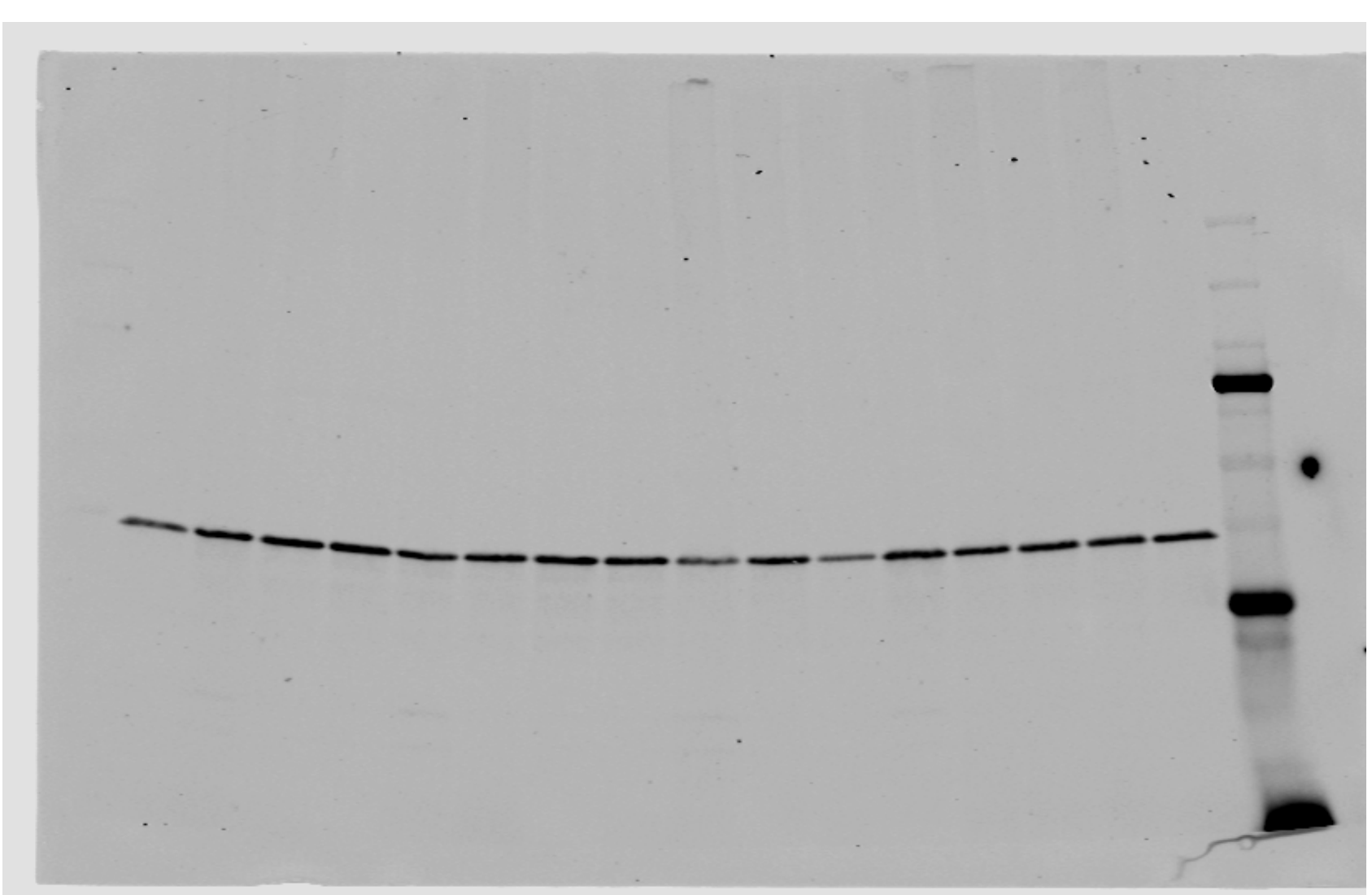

Fig. 3 Cx43 (3DPI and 7DPI)

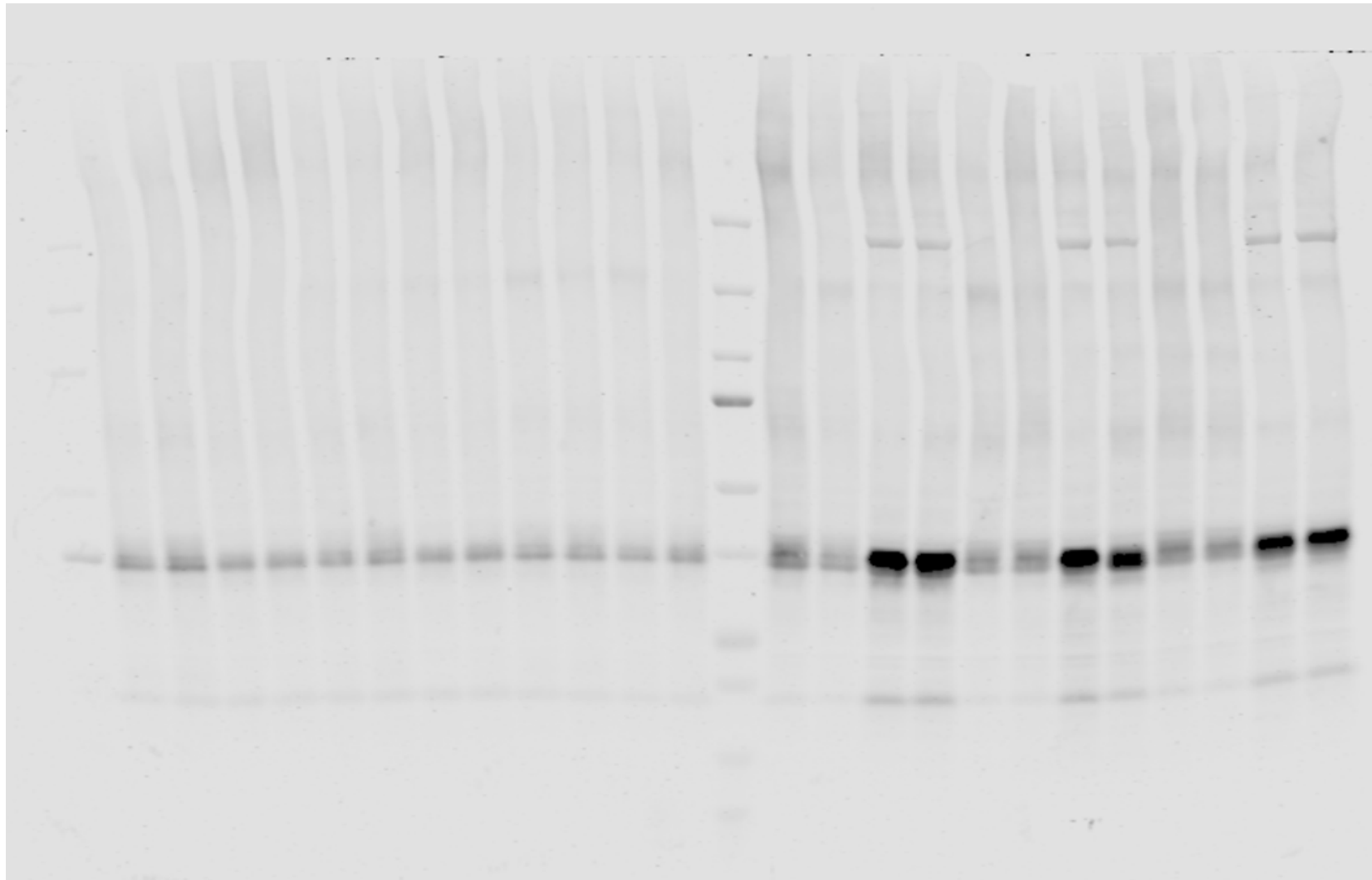

Fig. 3 p-Cx43 (3DPI and 7DPI)

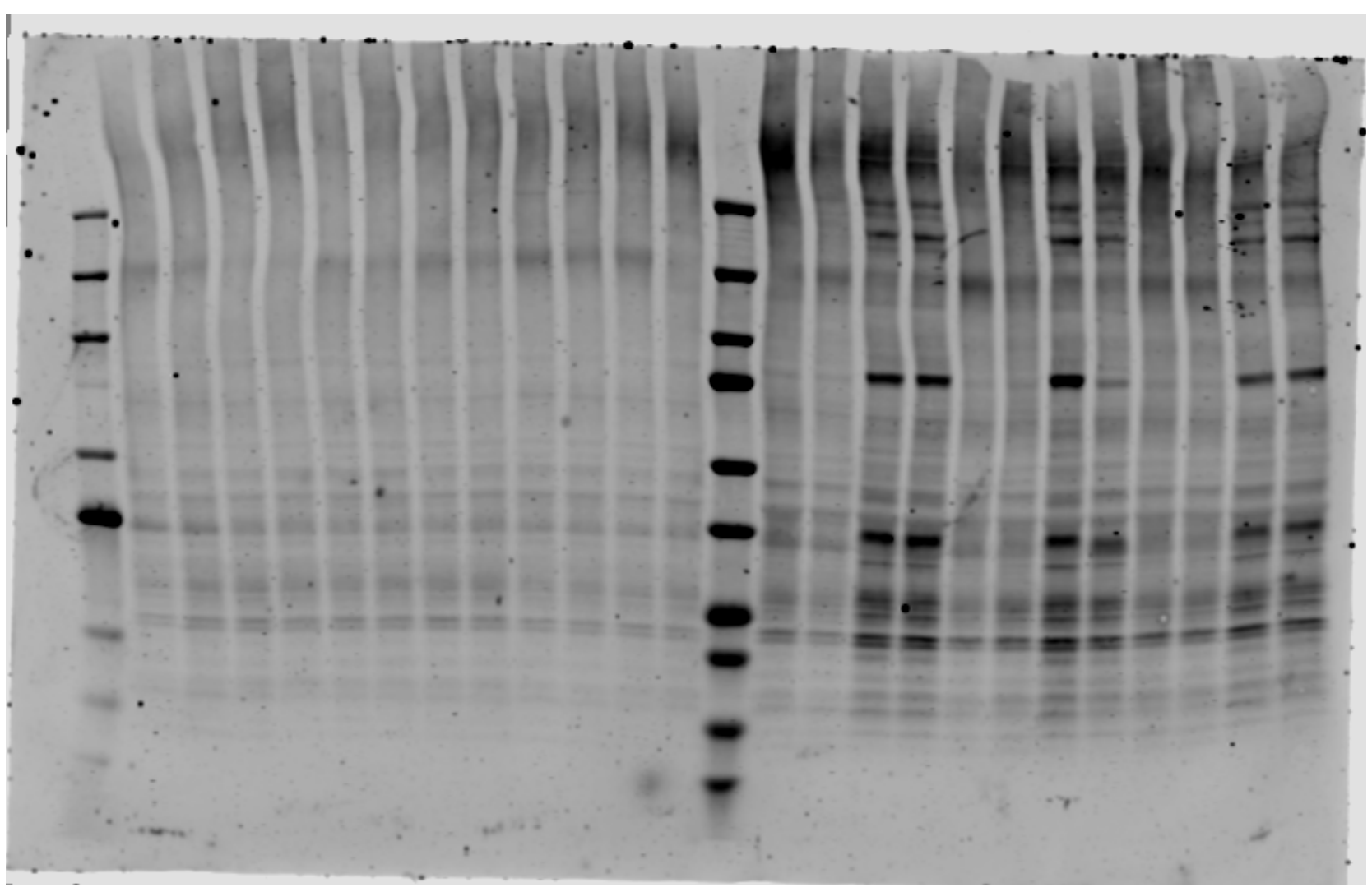

Fig. 3 GAPDH for Cx43 and p-Cx43 (3DPI and 7DPI)

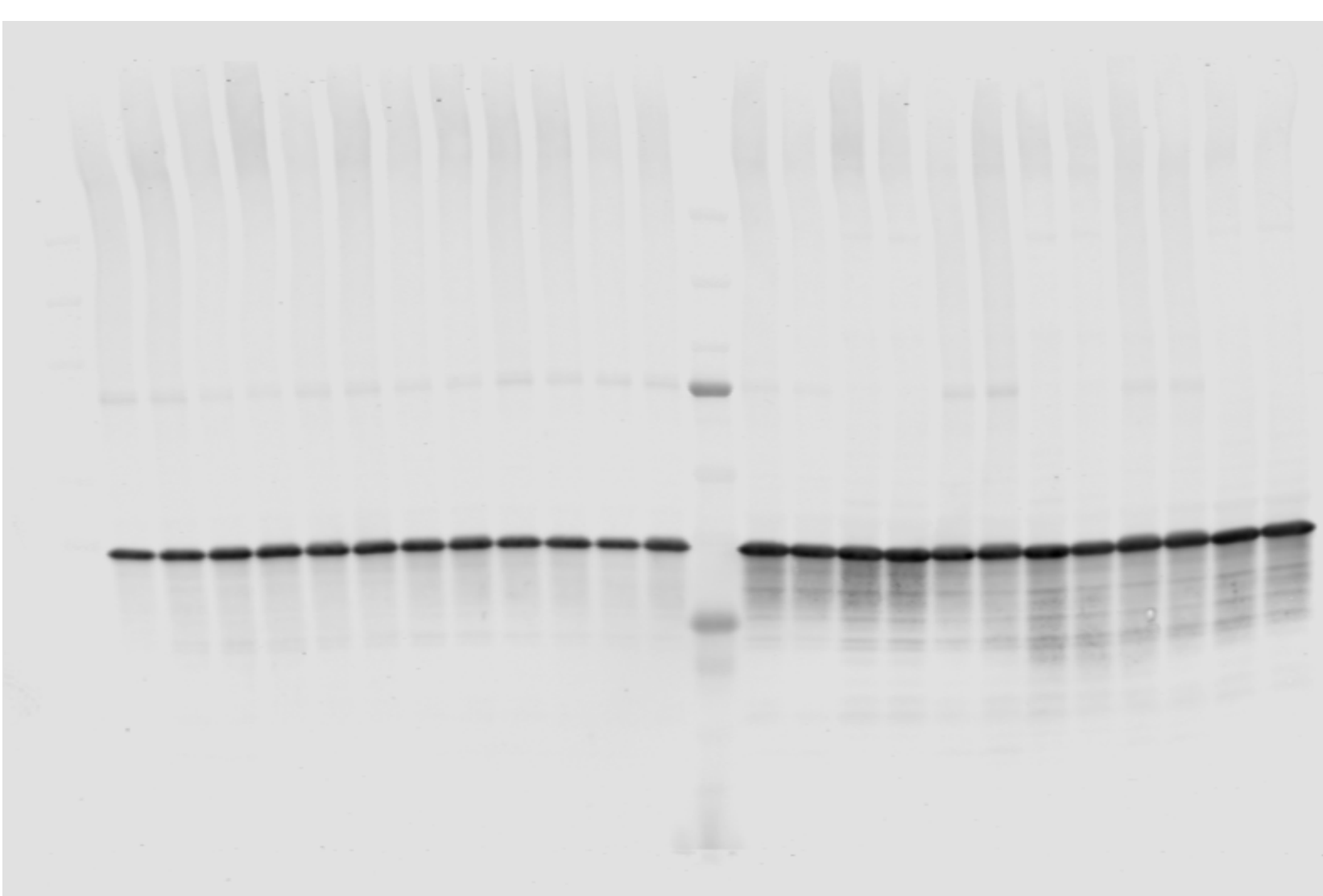

Fig. 3  PANX1 (3DPI)

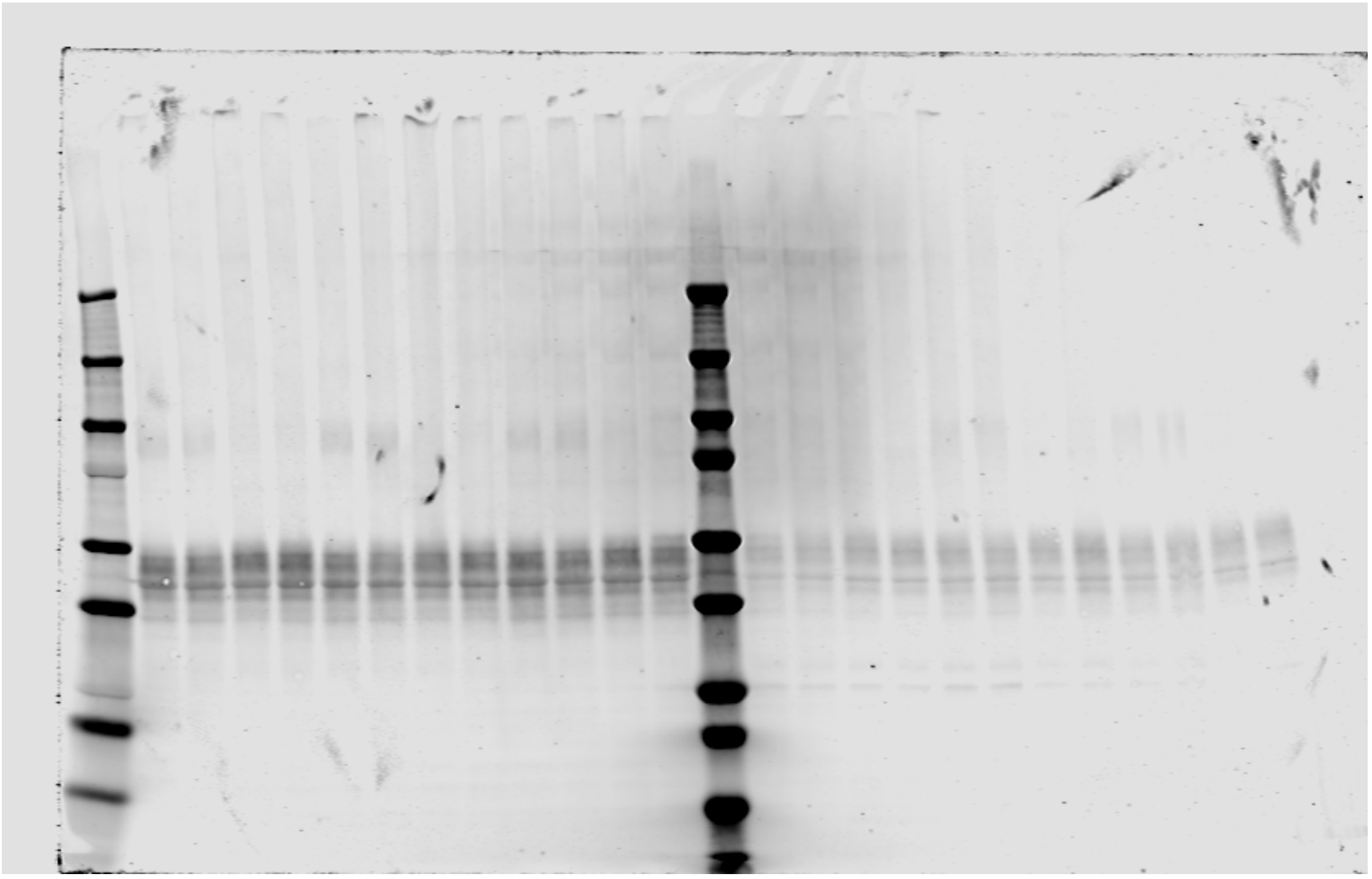

Fig. 3  GAPDH for PANX1 (3DPI)

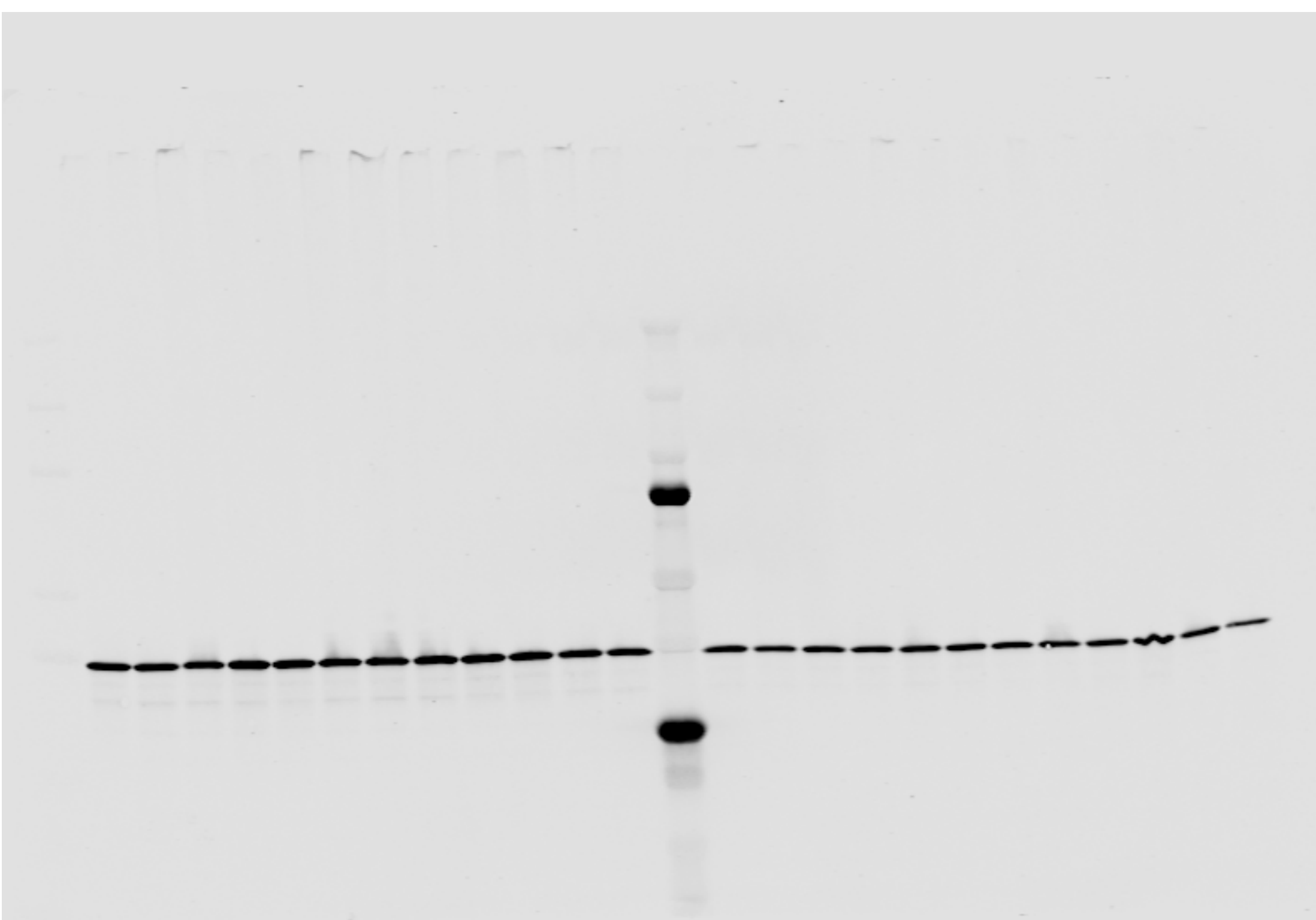

## Fig. 3  PANX1 (7DPI)

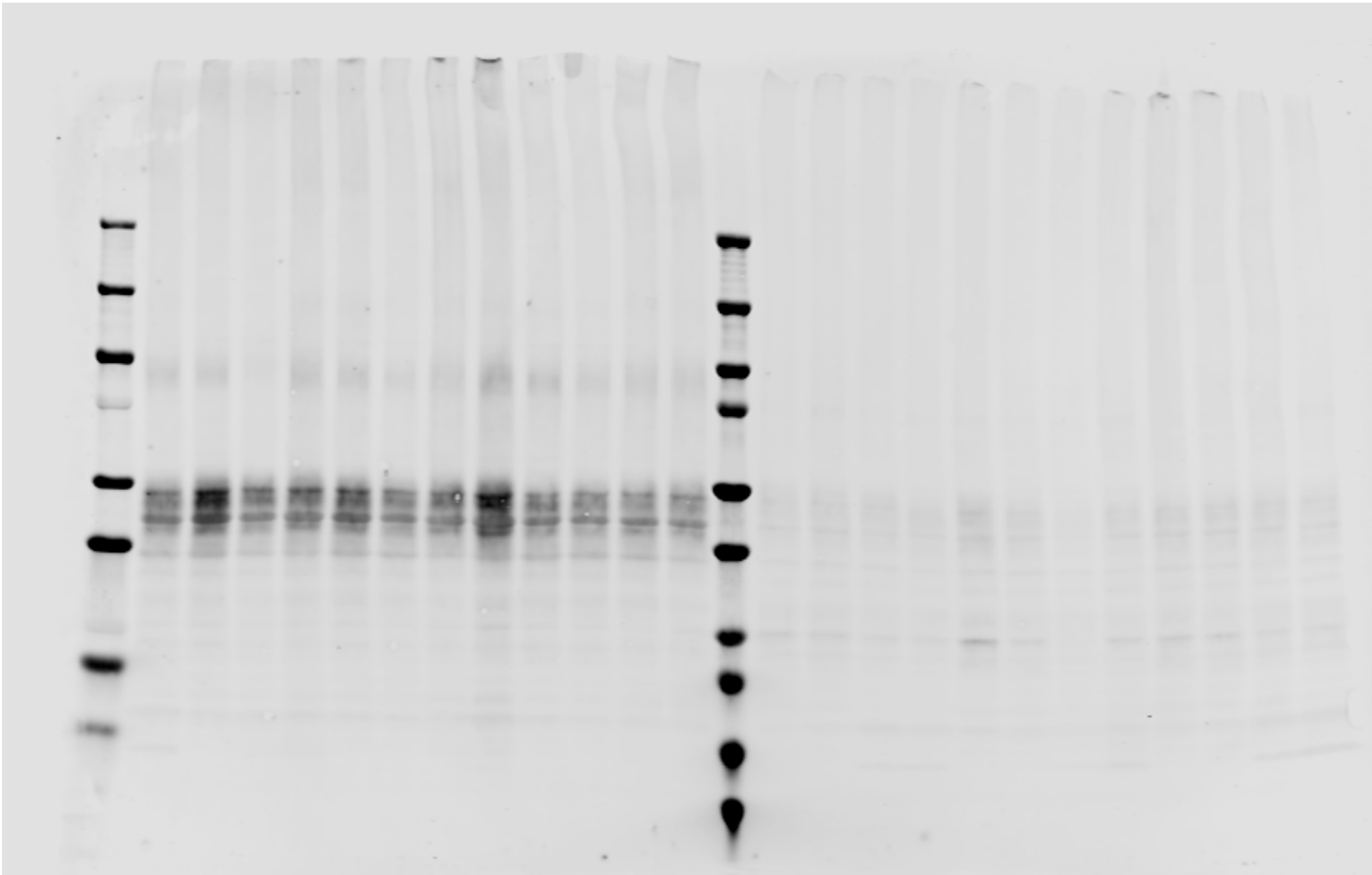

## Fig. 3  GAPDH for PANX1 (7DPI)

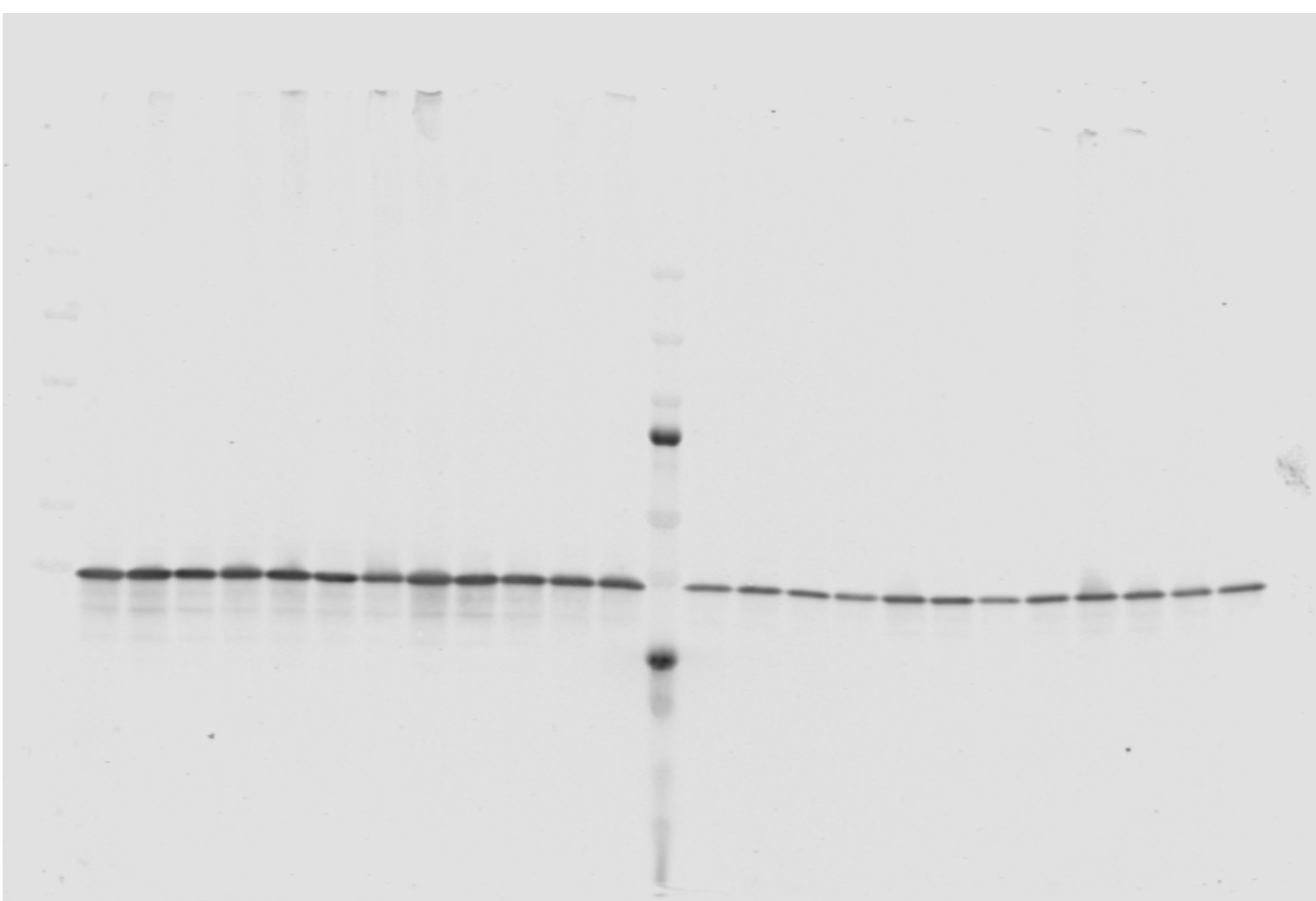

## Fig. 3 Cx37 (3DPI and 7DPI)

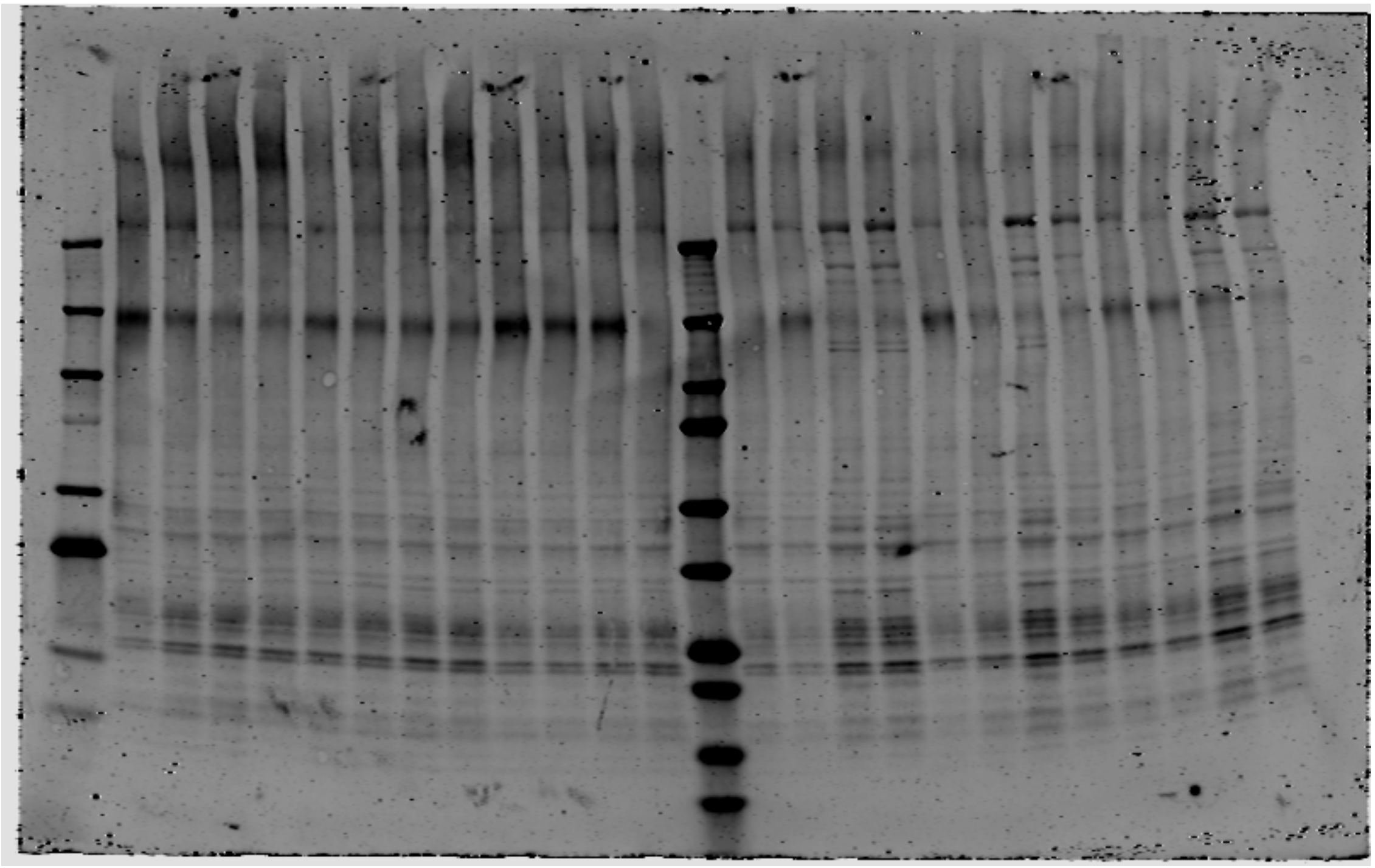

## Fig. 3 GAPDH for Cx37 (3DPI and 7DPI)

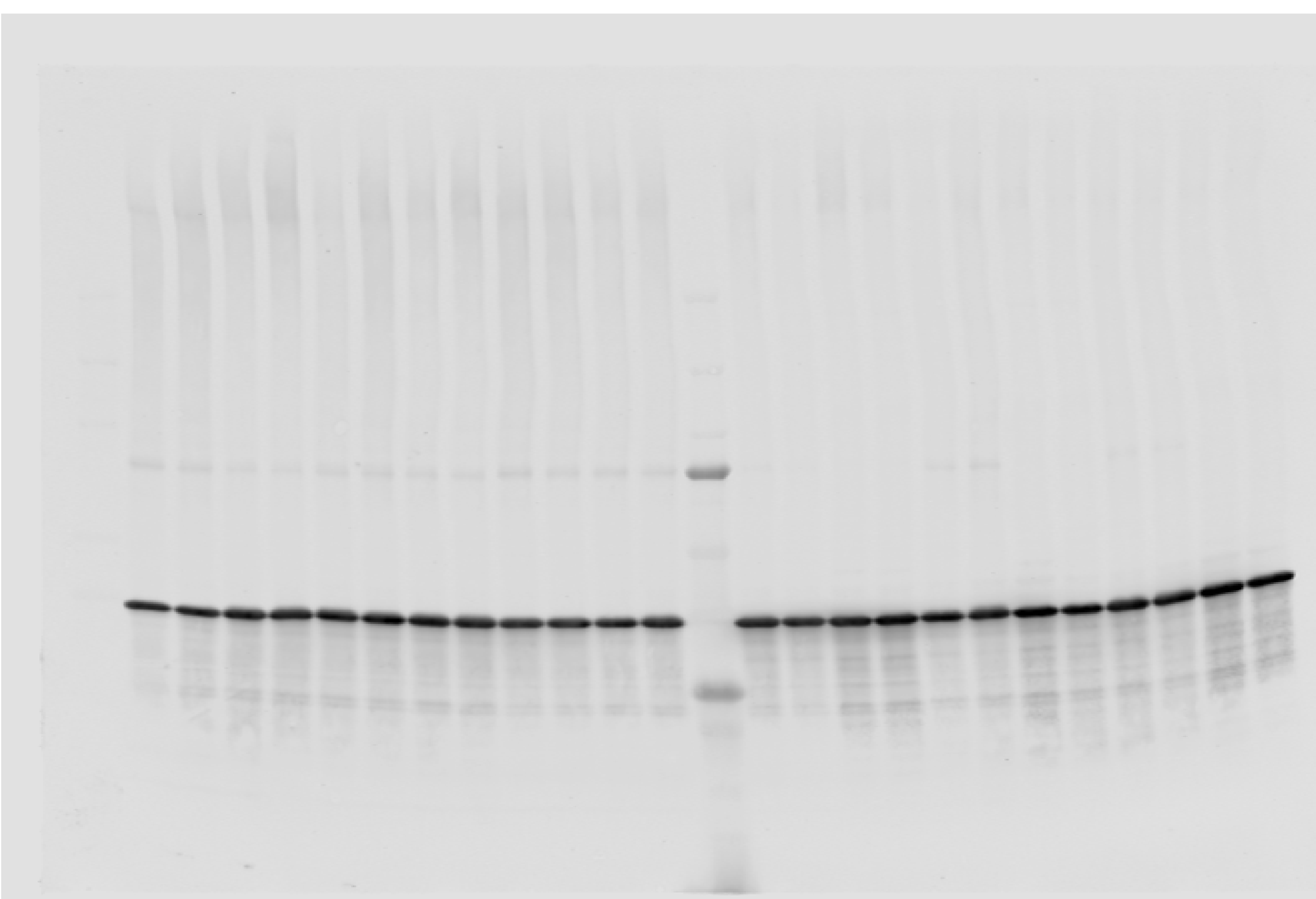

Fig. 3 Cx45 (3DPI)

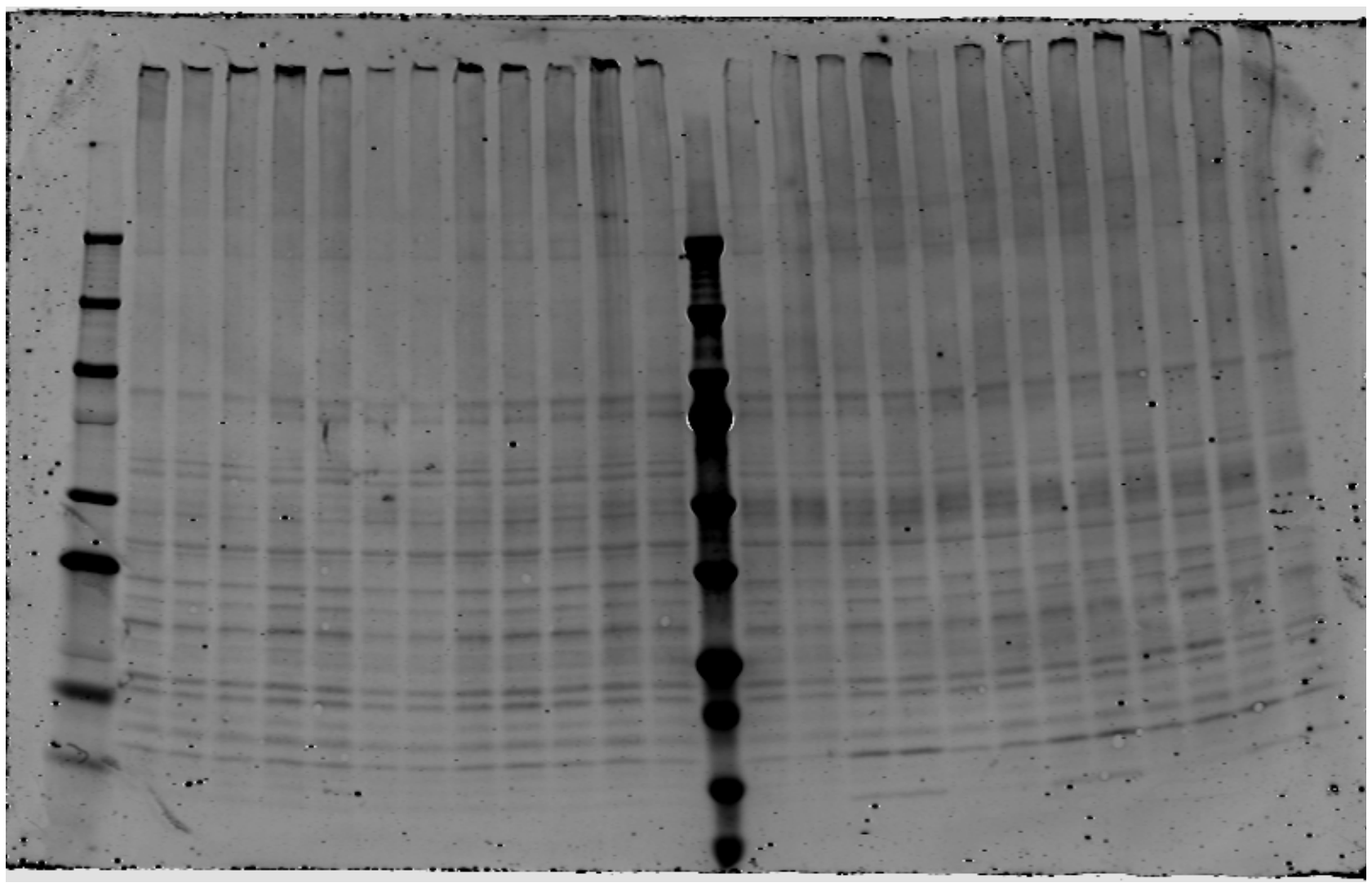

Fig. 3 Cx40 (3DPI)

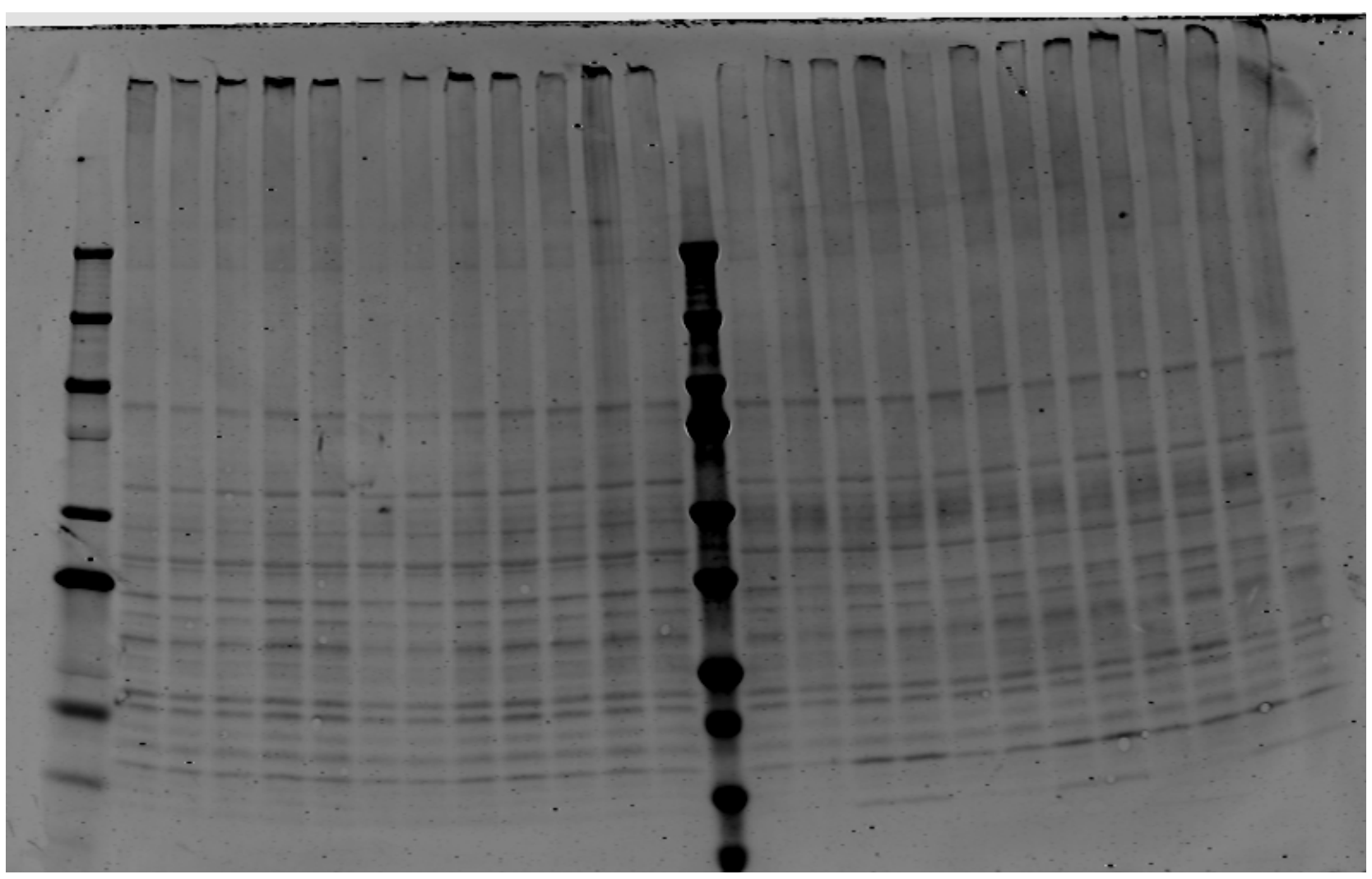

Fig. 3 GAPDH for Cx45 and Cx40 (3DPI)

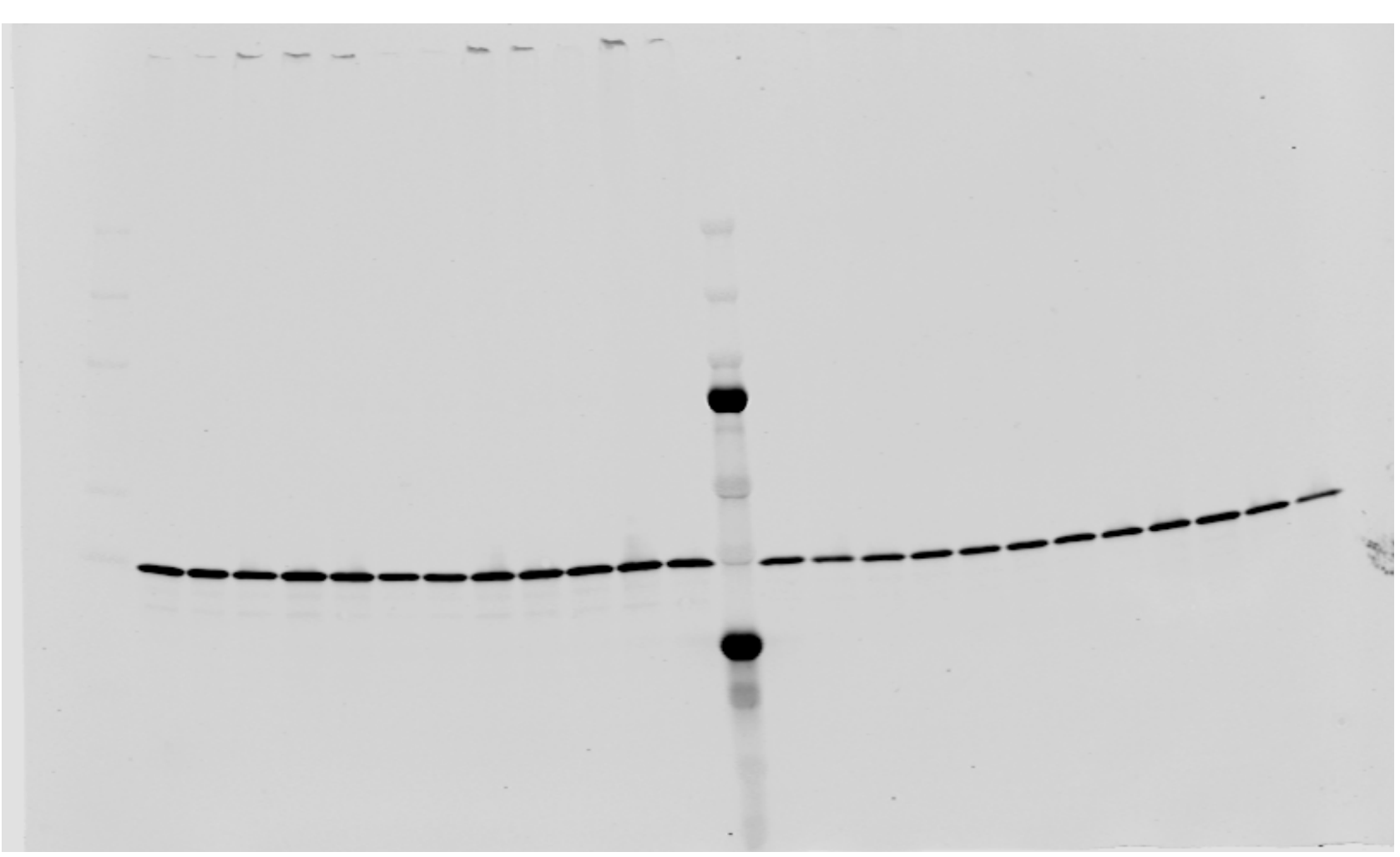

## Fig. 3 Cx45 (7DPI)

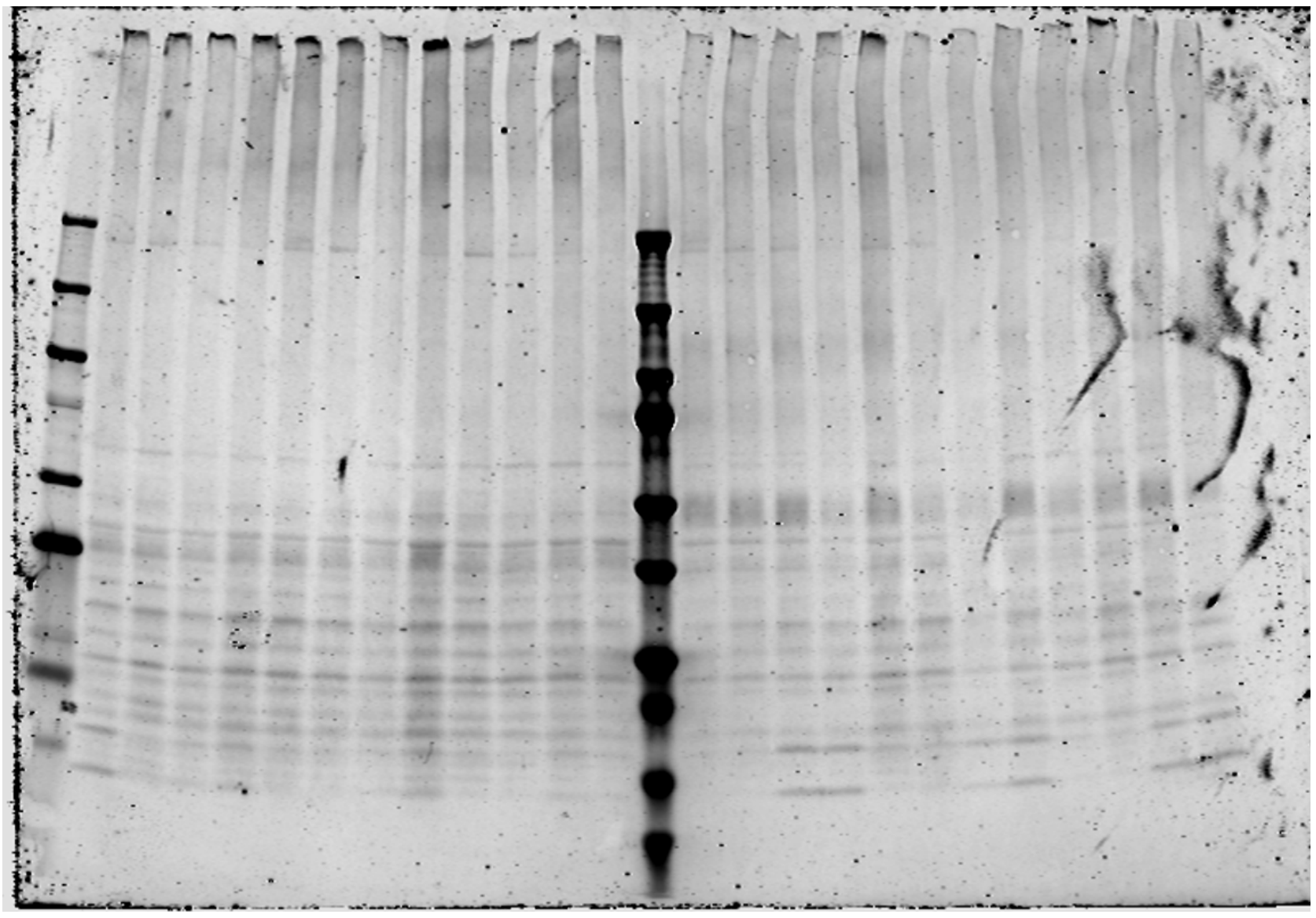

## Fig. 3 Cx40 (7DPI)

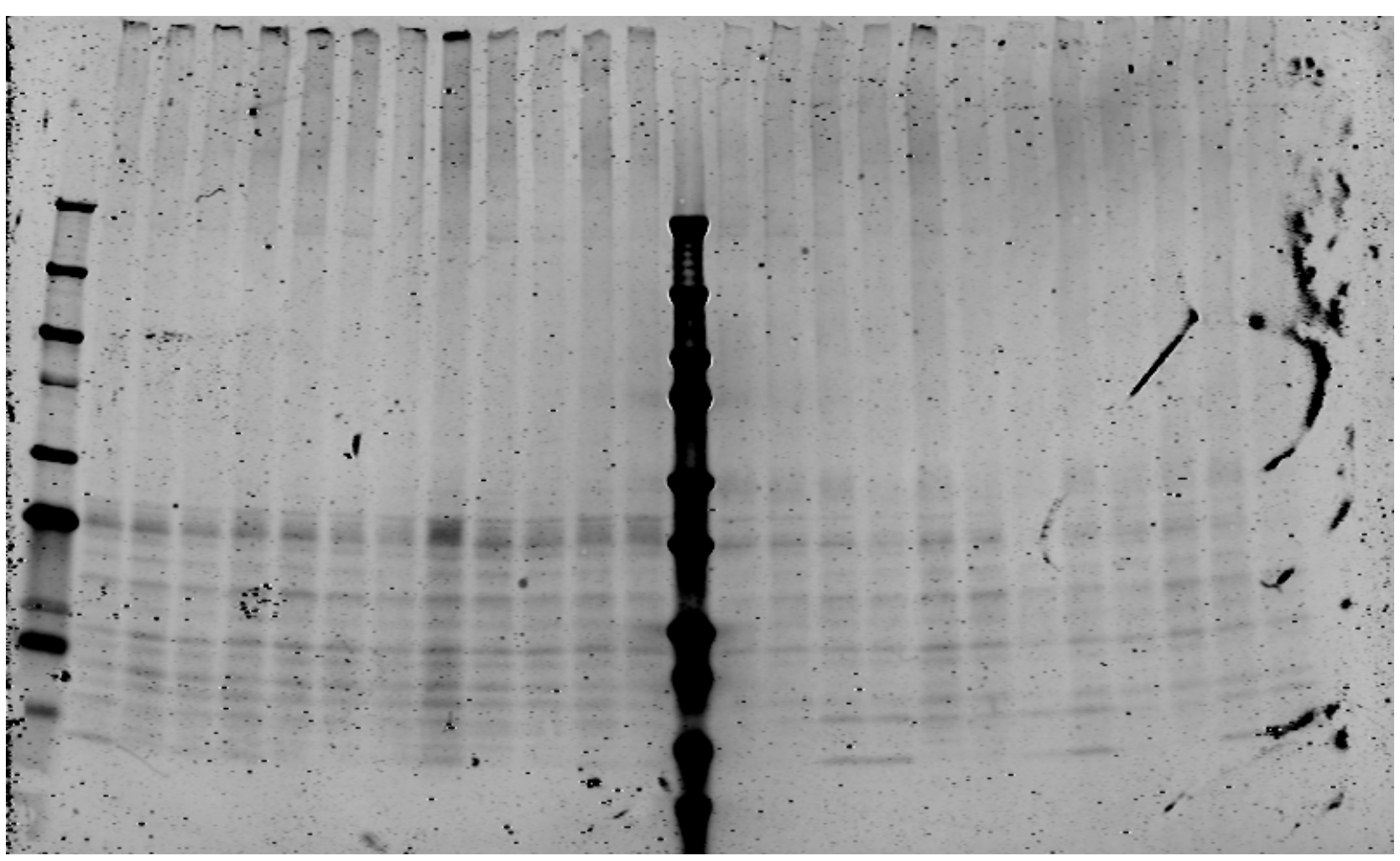

## Fig. 3 GAPDH for Cx45 (7DPI)

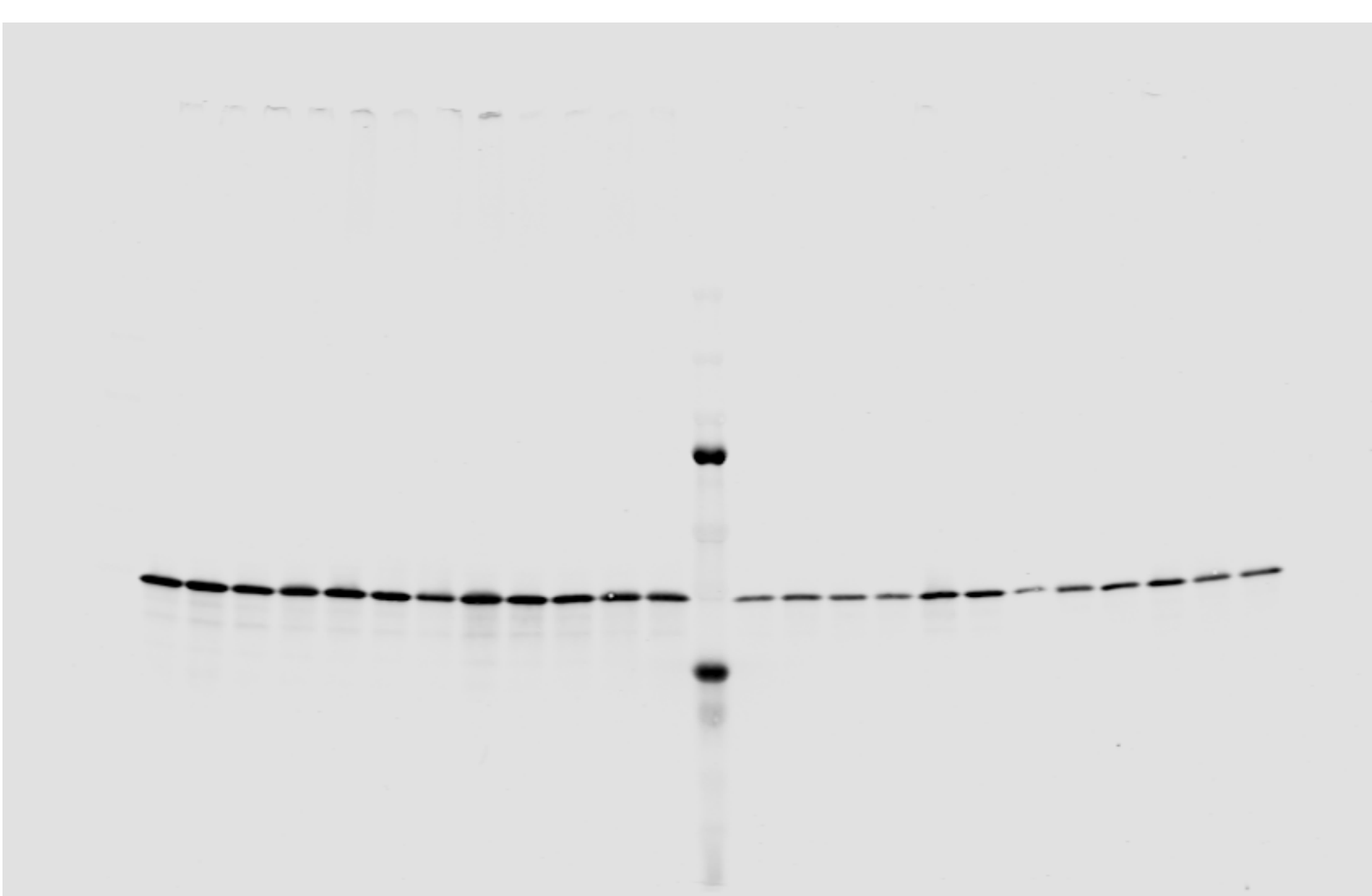

**Fig. 4 Cx43 (3DPI and 7DPI)**

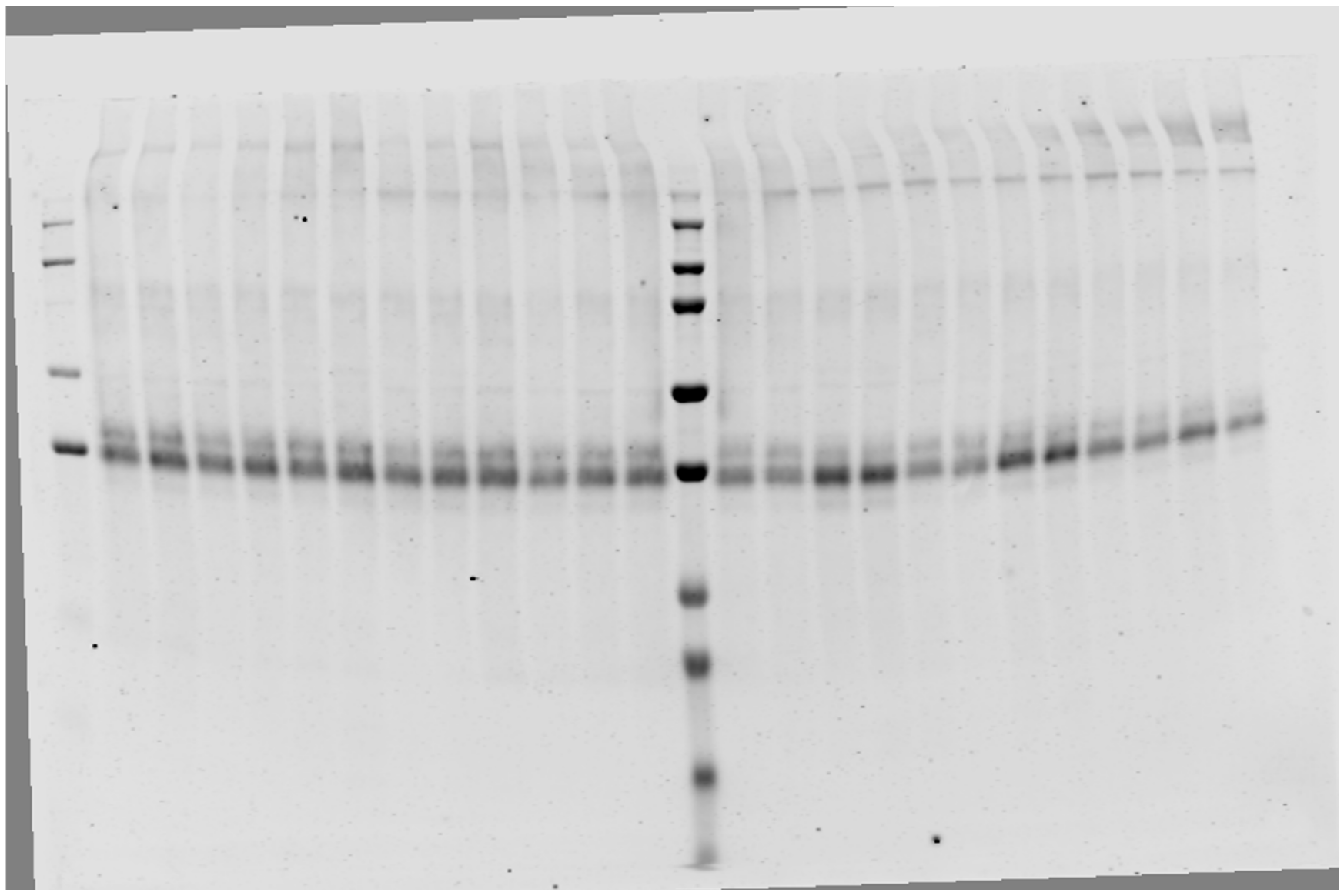

**Fig. 4 p-Cx43 (3DPI and 7DPI)**

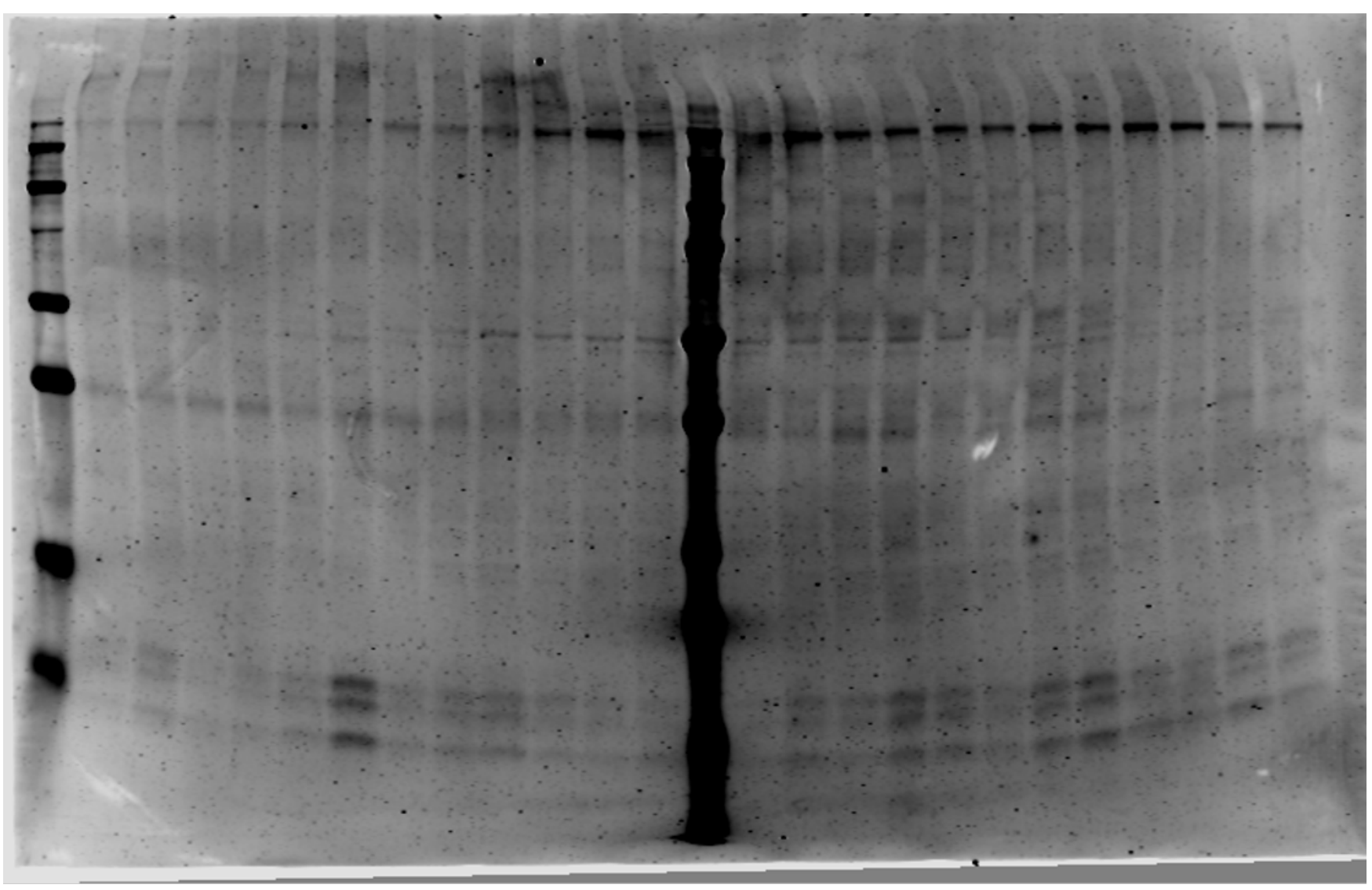

**Fig. 4 GAPDH for Cx43 and p-Cx43 (3DPI and 7DPI)**

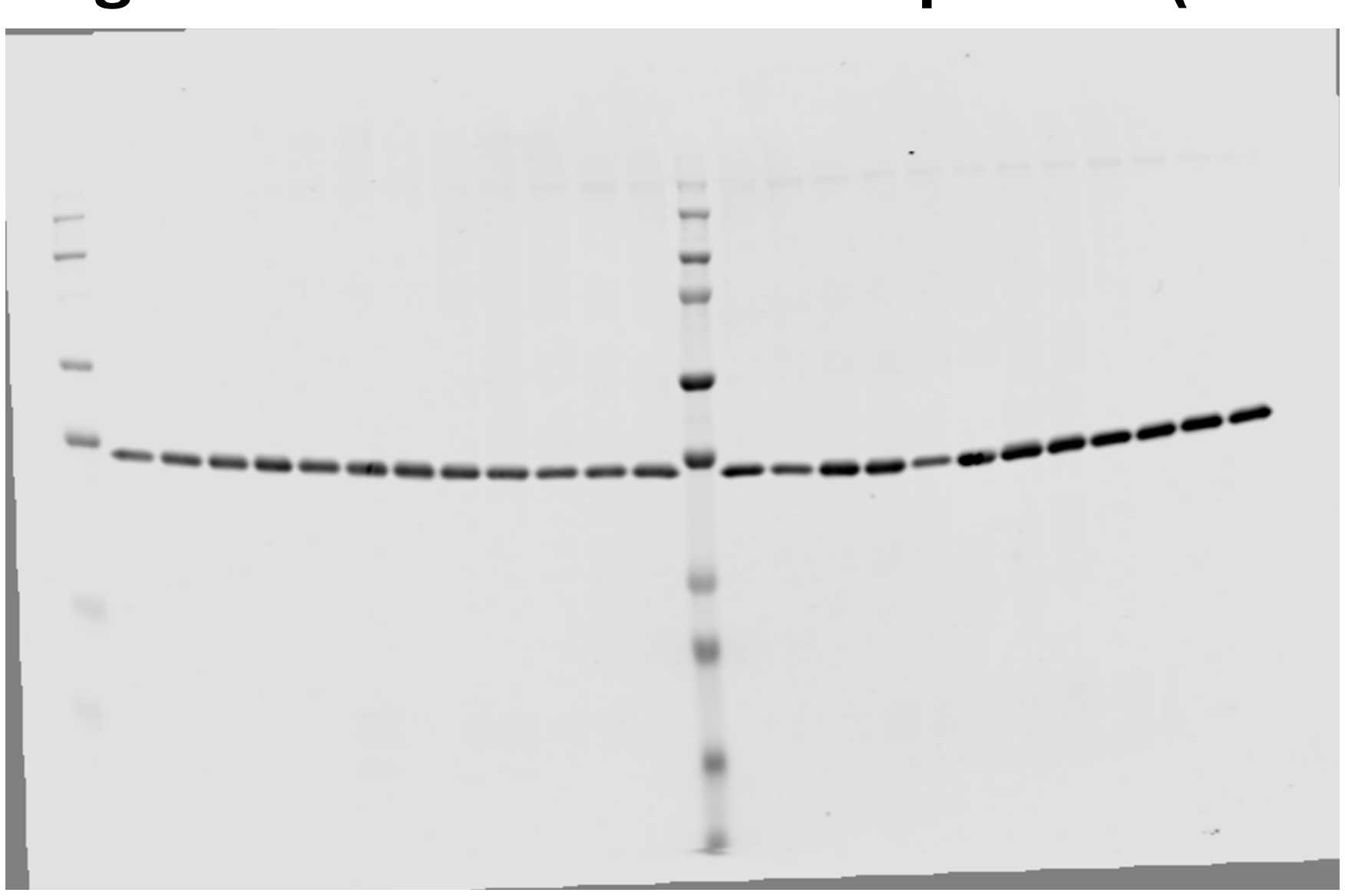

## Fig. 4  PANX1 (3DPI)

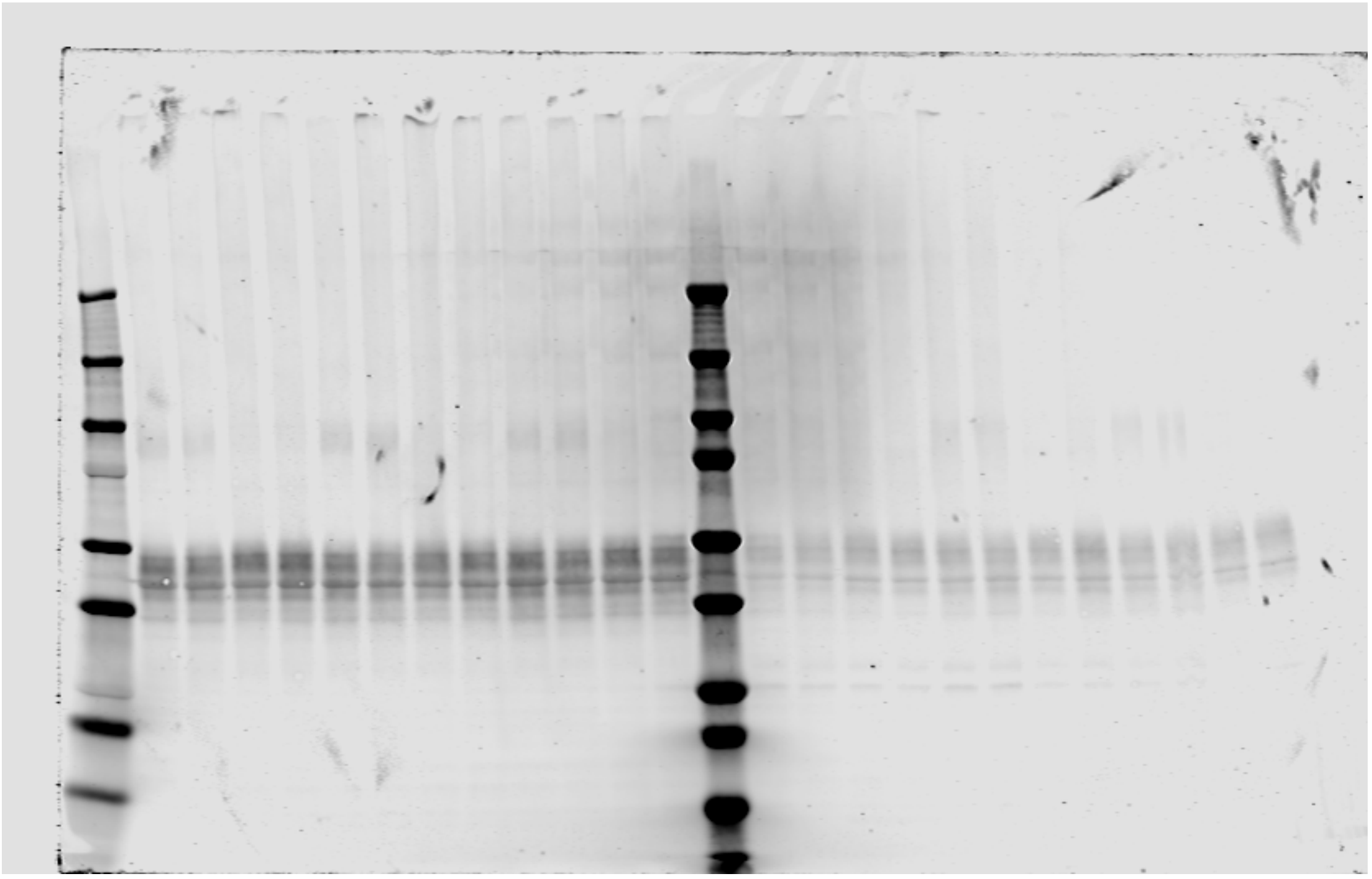

## Fig. 4 GAPDH for PANX1 (3DPI)

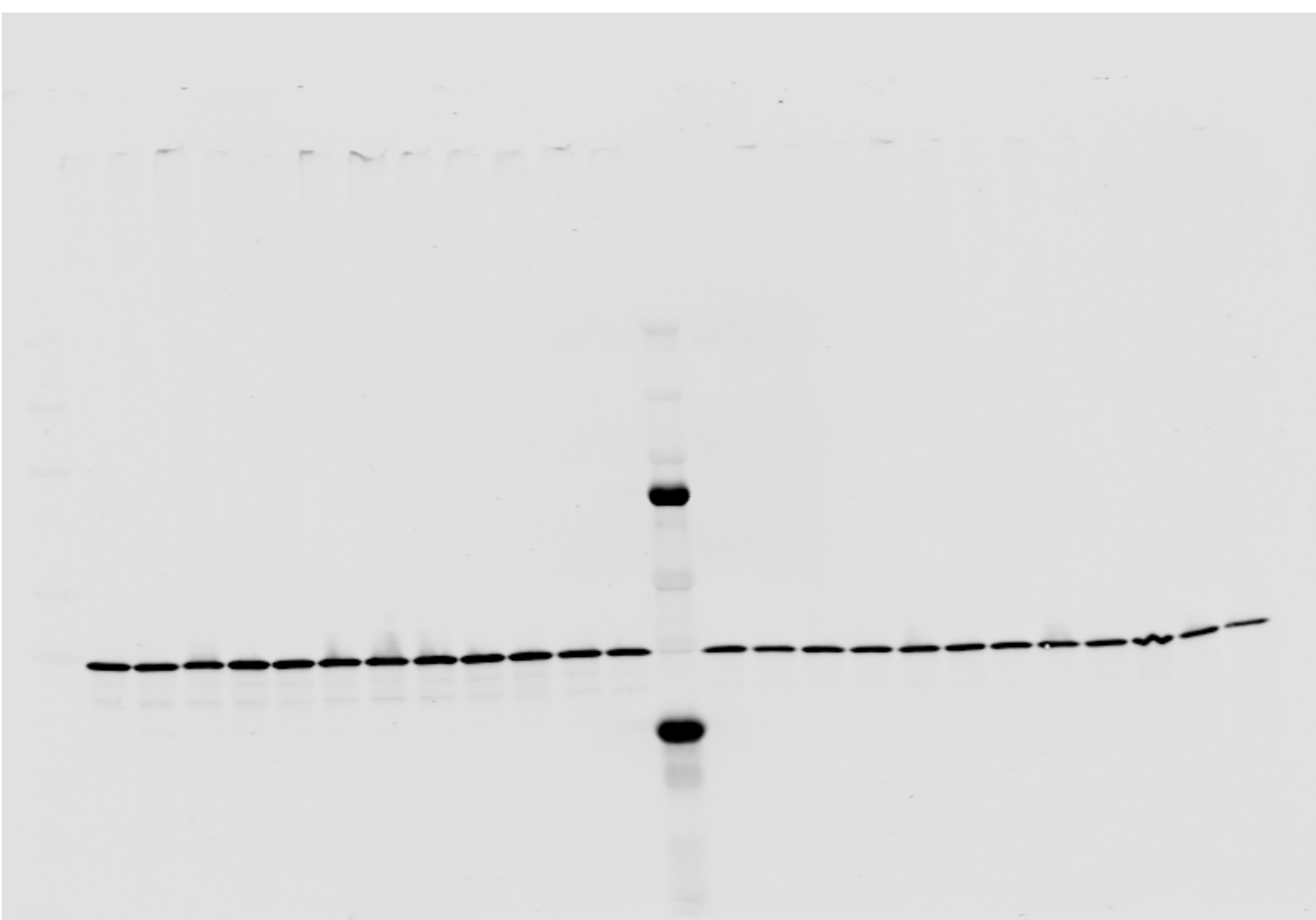

## Fig. 4 PANX1 (7DPI)

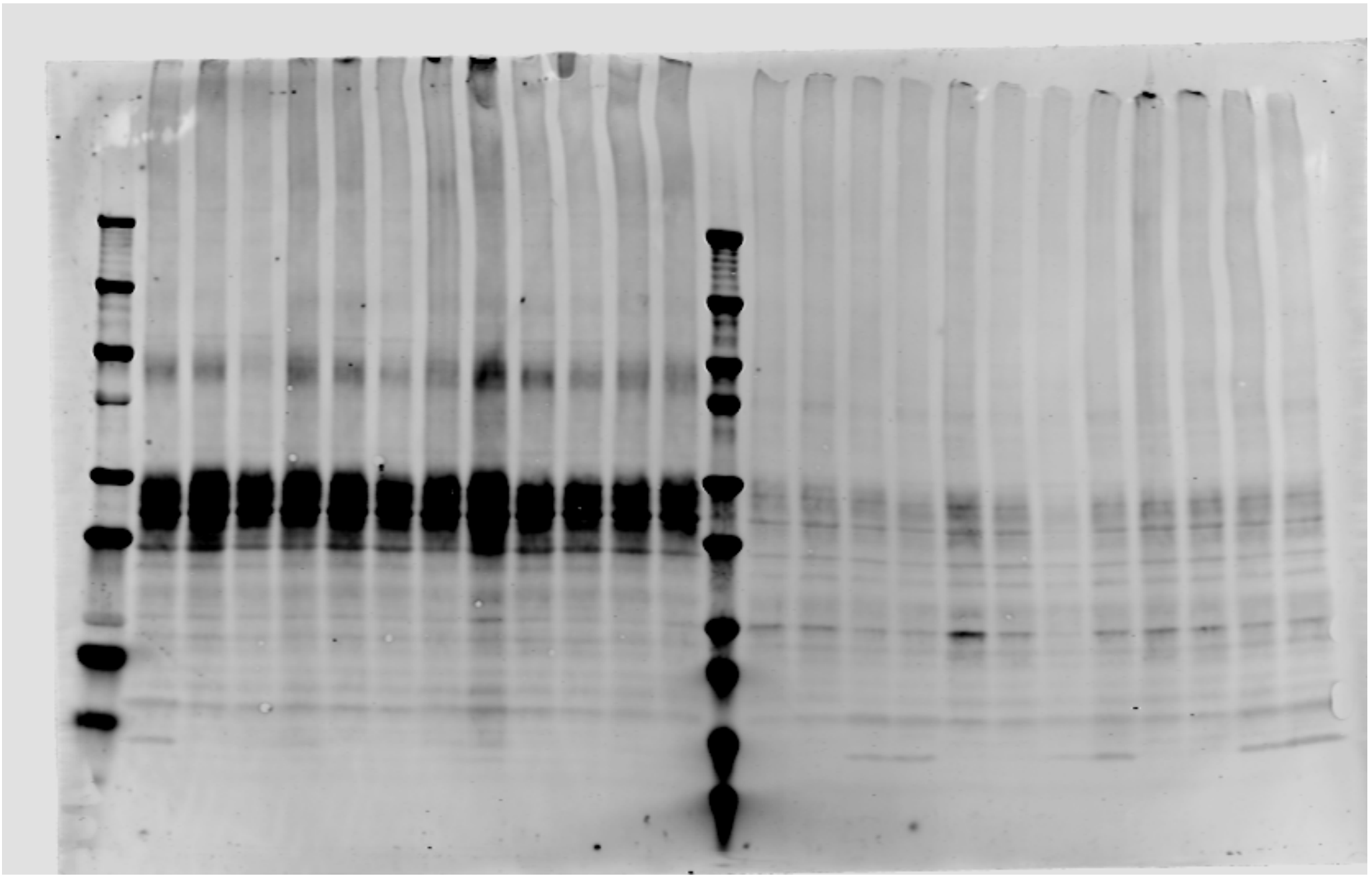

## Fig. 4 GAPDH for PANX1 (7DPI)

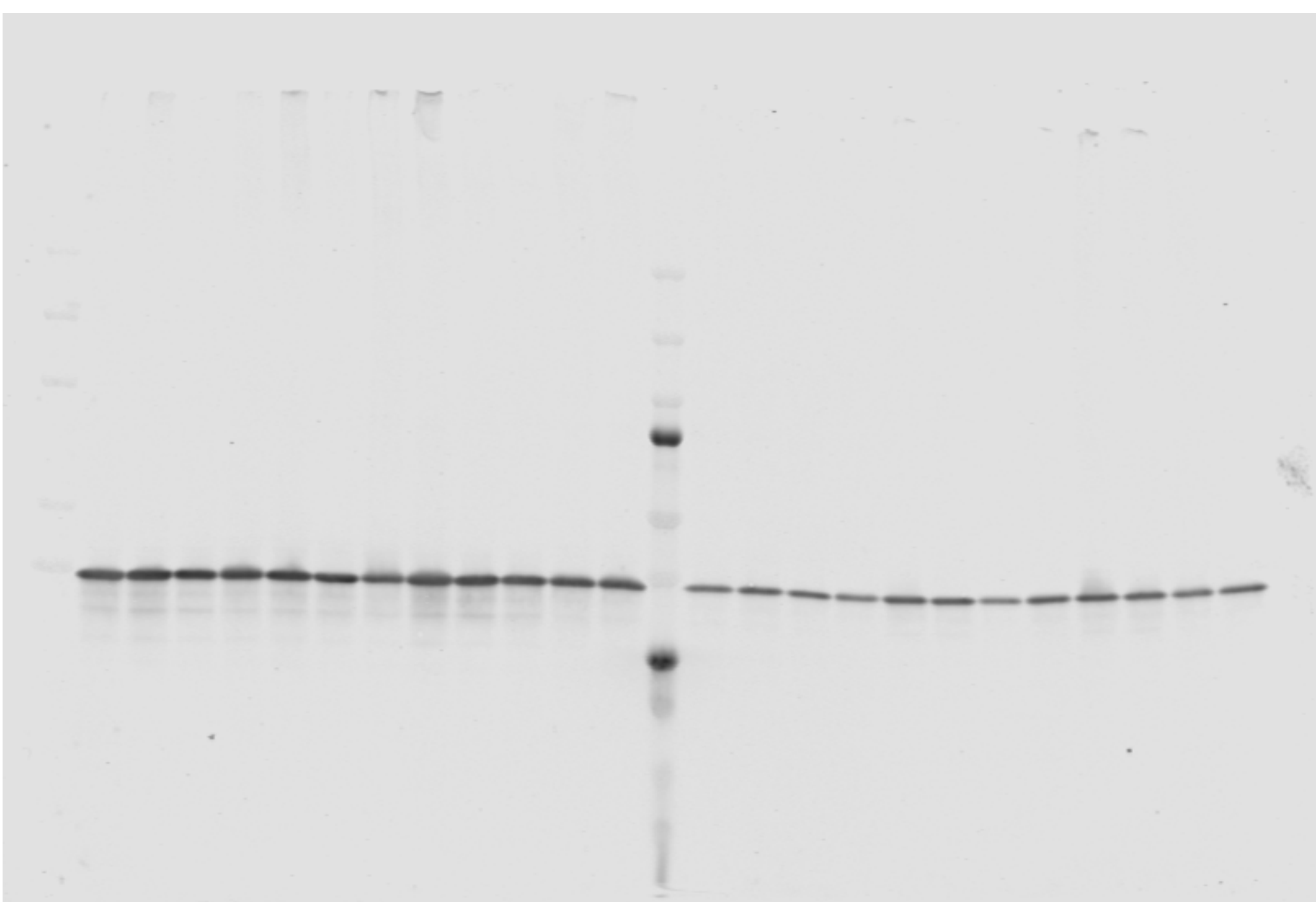

Fig. 4 Cx37 (3DPI)

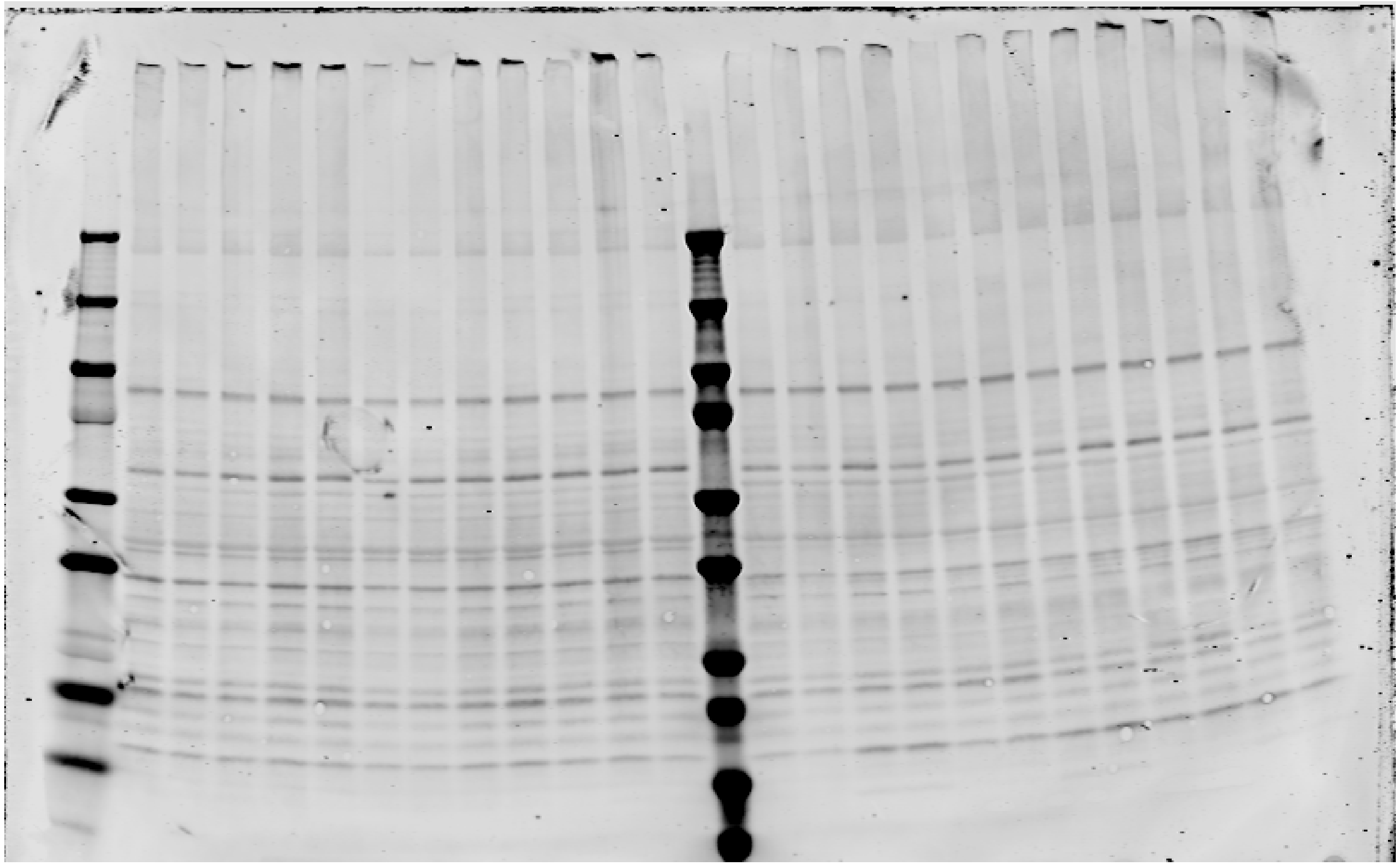

Fig. 4 Cx45 (3DPI)

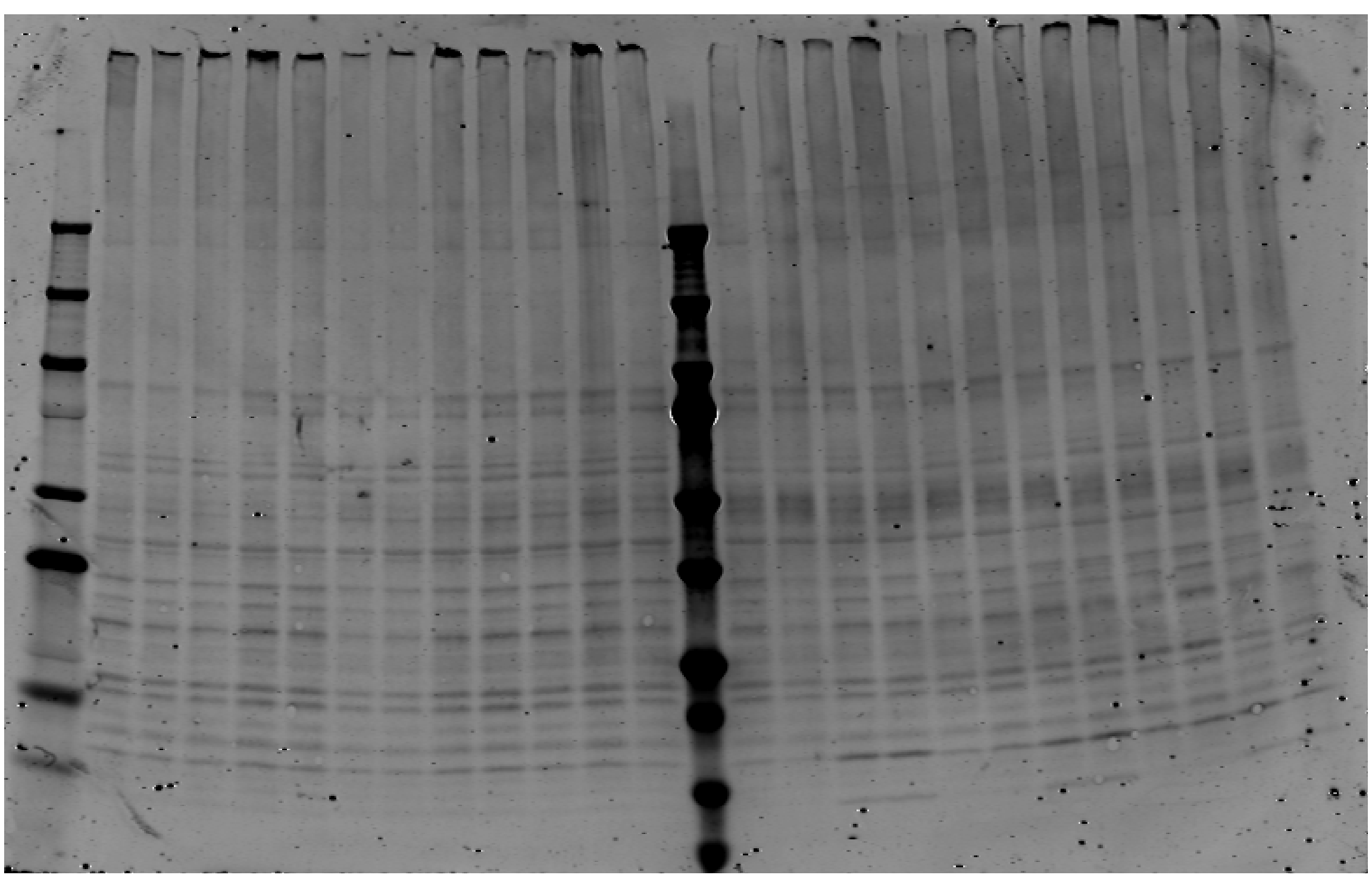

## Fig. 4 Cx40 (3DPI)

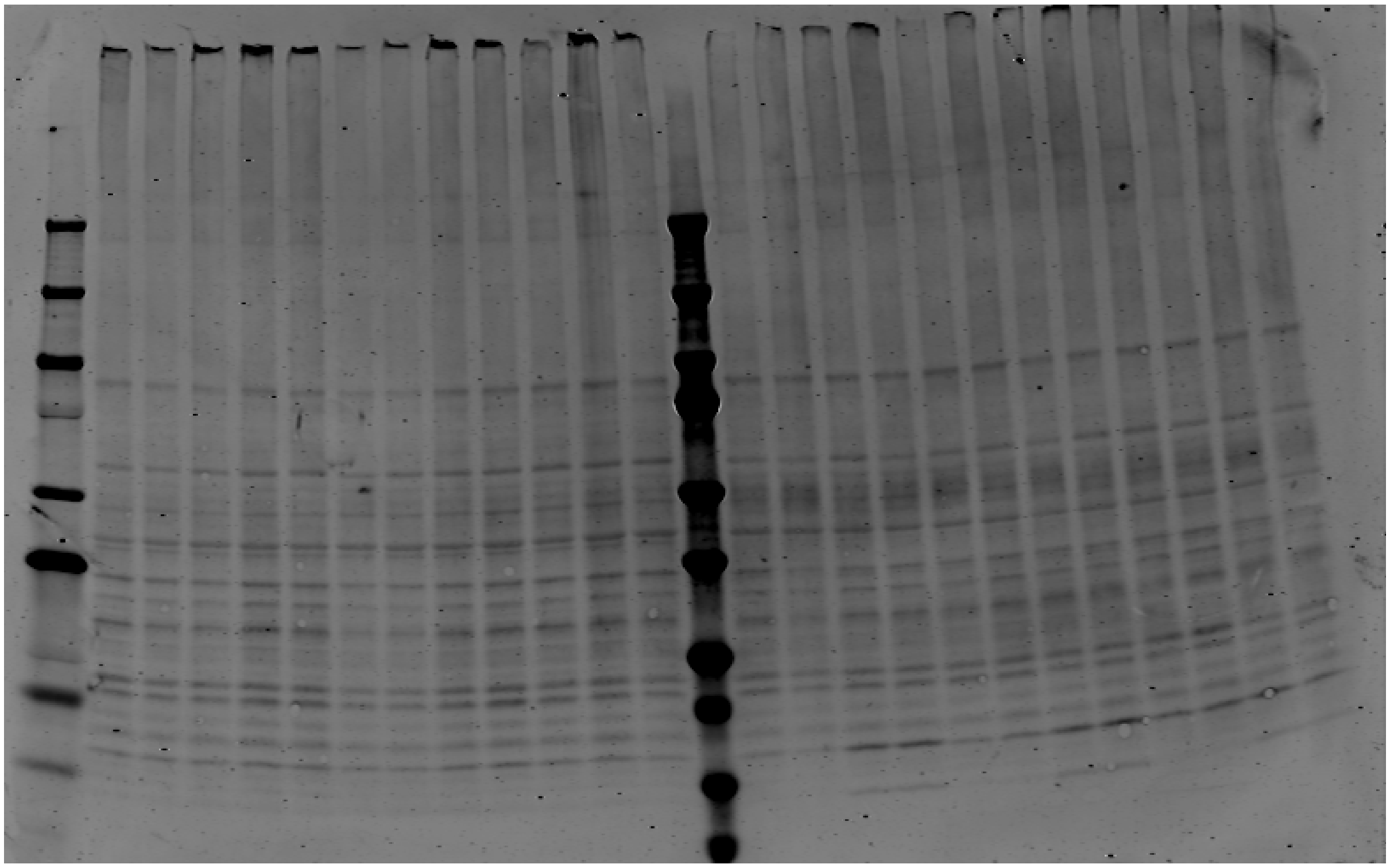

## Fig. 4 GAPDH for Cx37, Cx45 and Cx40 (3DPI)

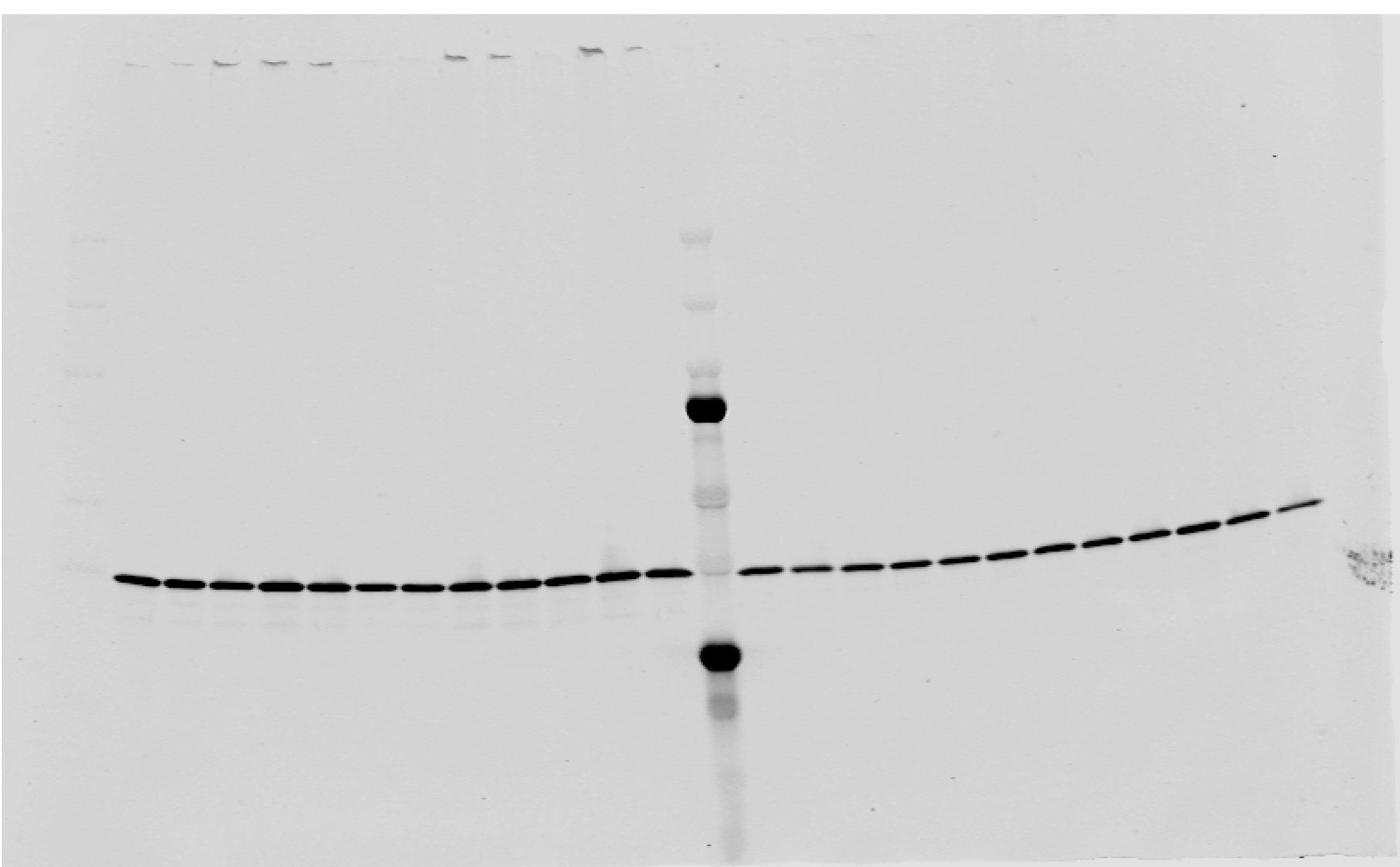

**Fig. 4 Cx37 (7DPI)**

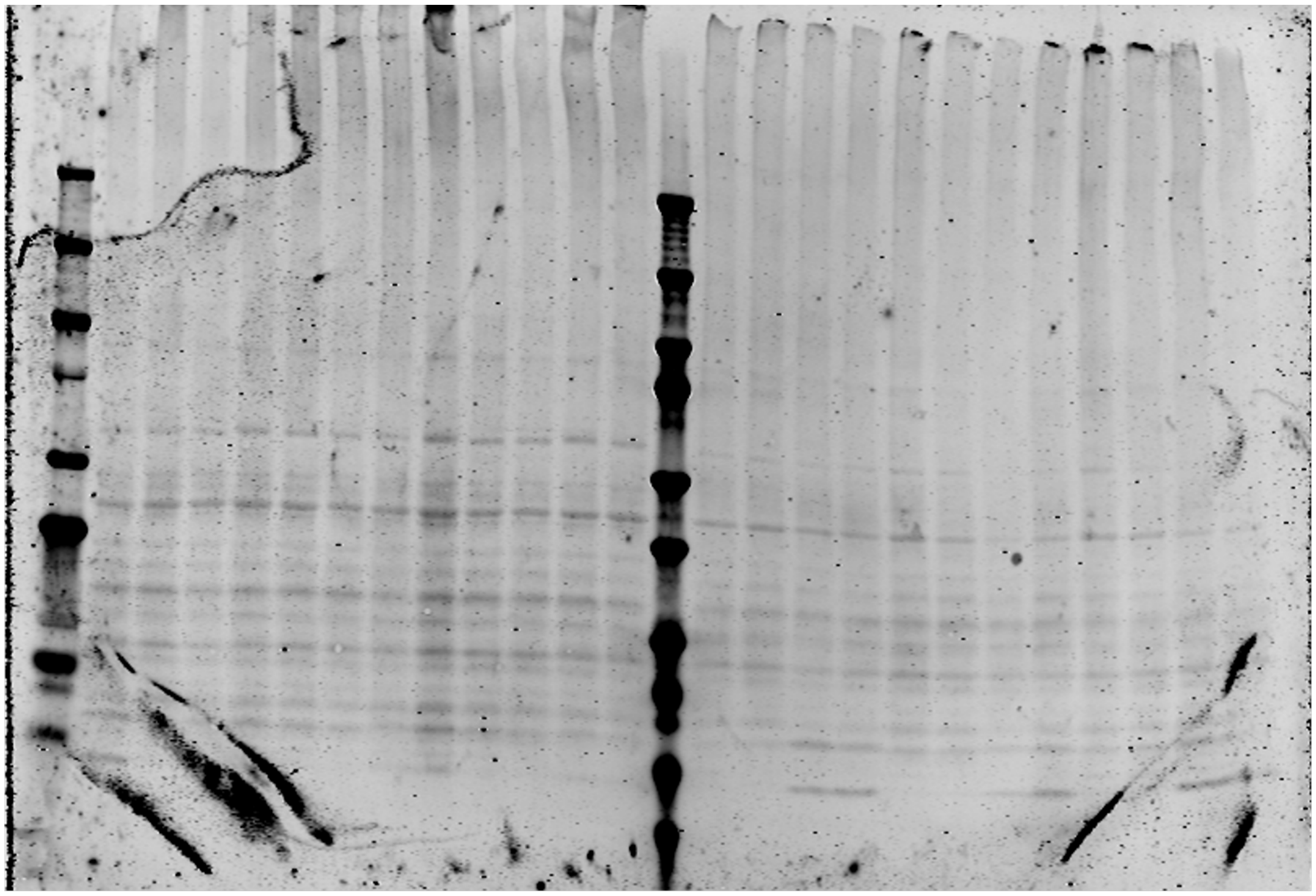

**Fig. 4 Cx45 (7DPI)**

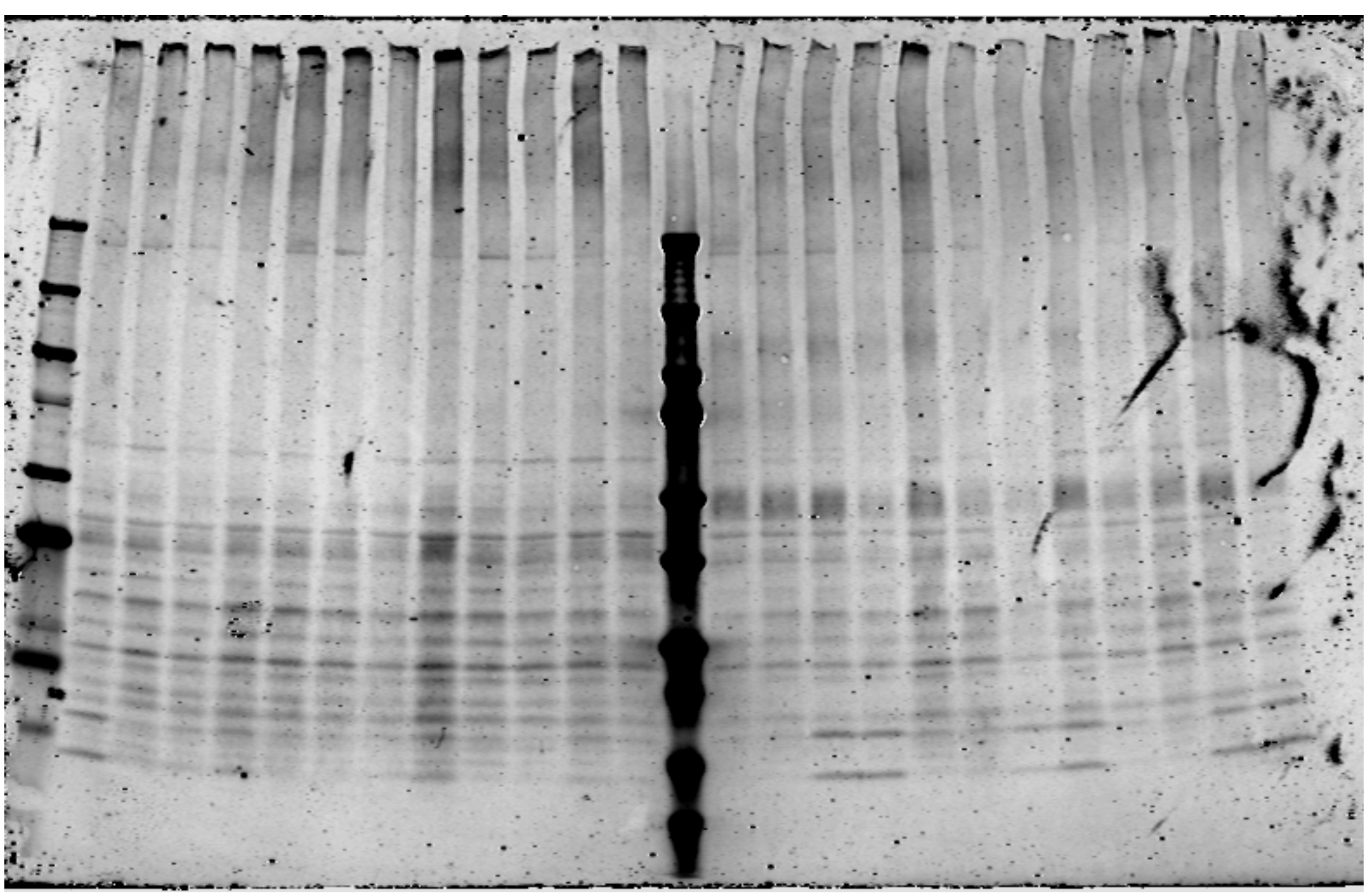

**Fig. 4 Cx40 (7DPI)**

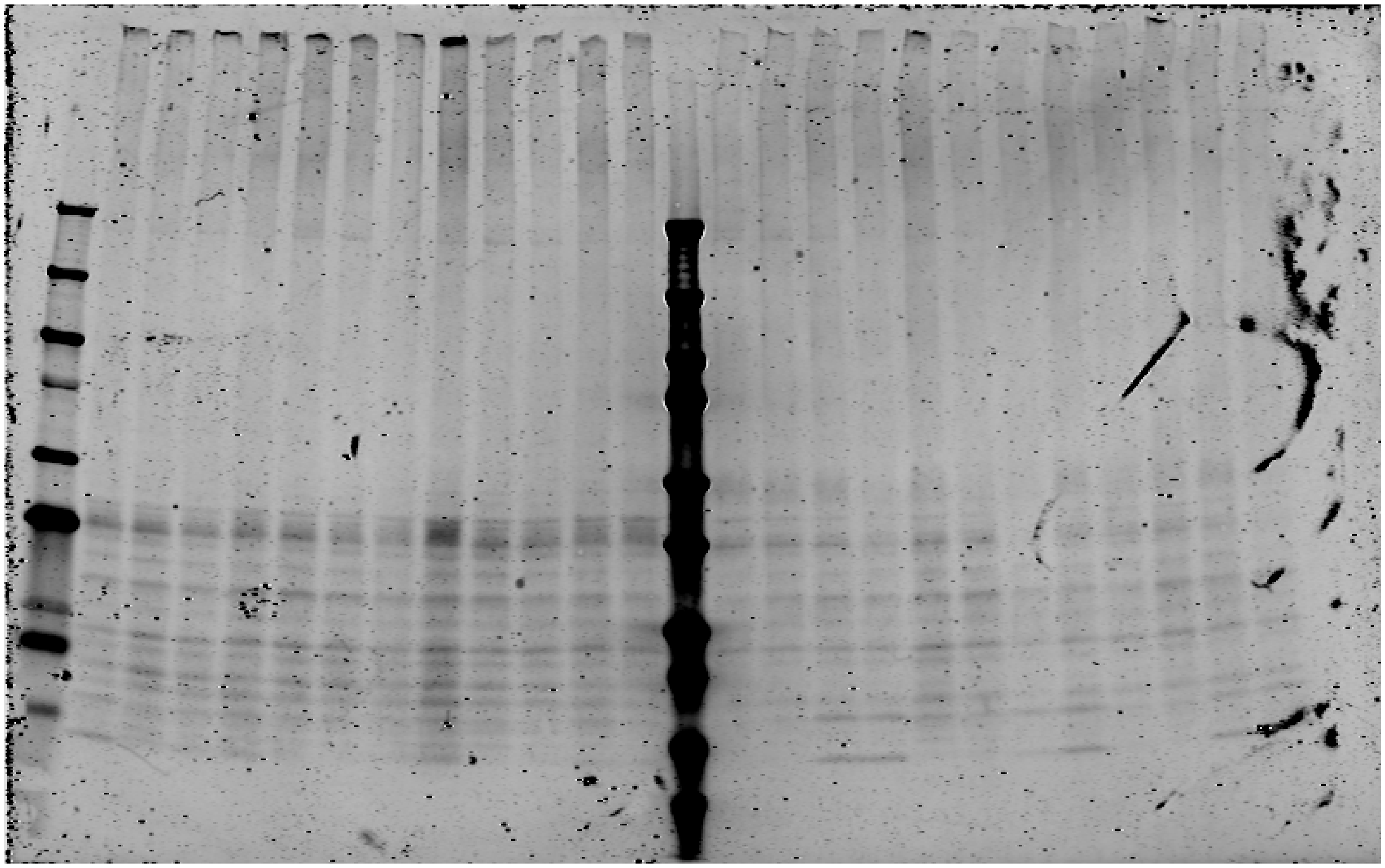

**Fig. 4 GAPDH for Cx37, Cx45 and Cx40 (7DPI)**

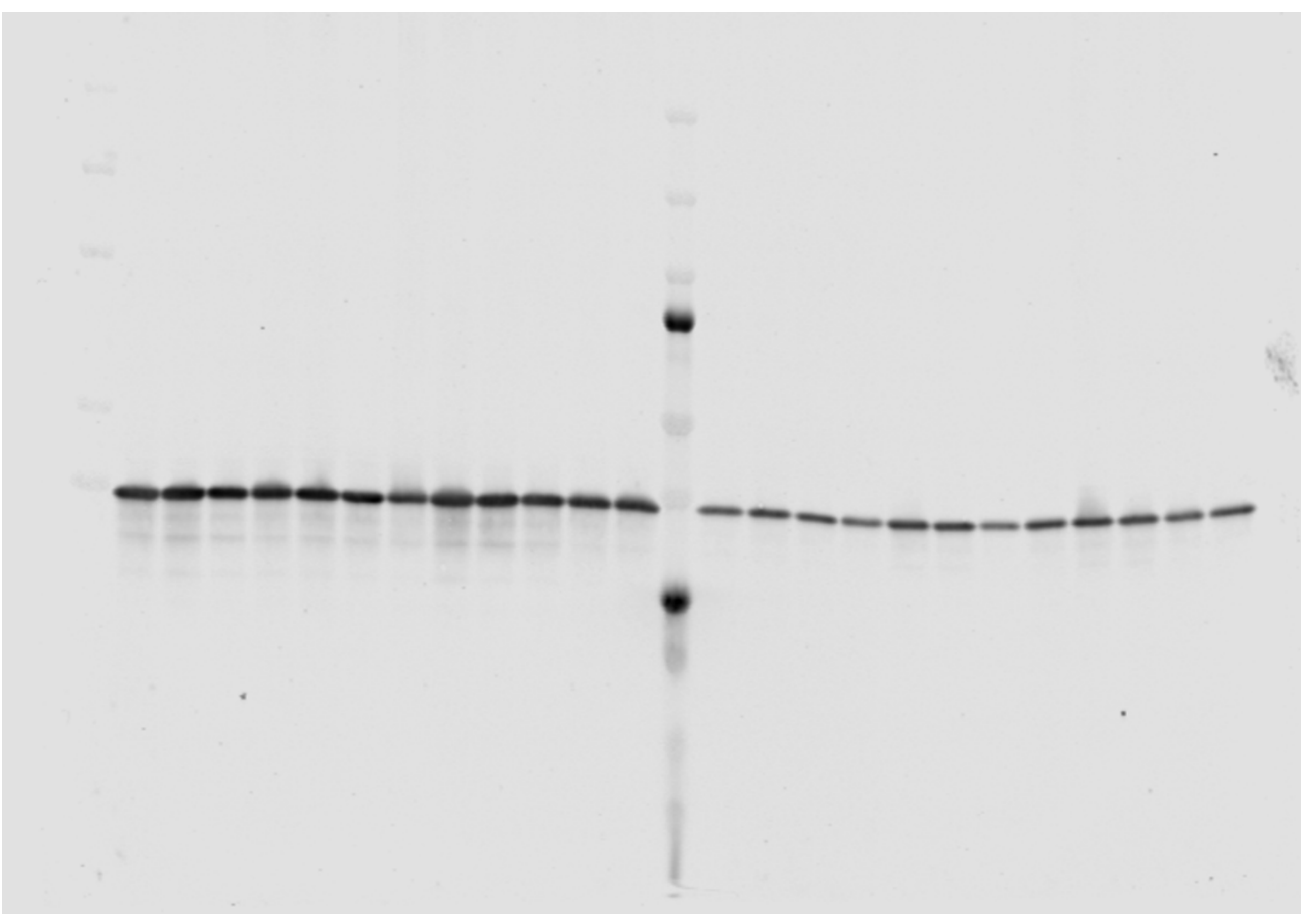

Supplement: Supplementary file 1 [file 12035_2026_6266_MOESM1_ESM.pdf]
