# Supplementary material for: Latent HIV Infection of BBB Pericytes Enhances Gap Junction-mediated Crosstalk with Endothelial Cells
Source: Mol Neurobiol. 2026 Oct 16;63(1):973. doi: 10.1007/s12035-026-06266-0 (PMC13675307; doi:10.1007/s12035-026-06266-0)
Supplement: Supplementary file 2 [file 12035_2026_6266_MOESM2_ESM.pdf]

Supplementary Material

Latent HIV Infection of BBB Pericytes Disrupts Gap Junction-Mediated Crosstalk with Endothelial Cells

Sarah Schmidlin [1], Oandy Naranjo [1], Silvia Torices [1], Nikolai Fattakhov [1], Monika Gilicinska [1], Masha Minevich [1], Kyra Schindler [2], Destiny Tiburcio [3], Olivia M. Osborne [1], Arpan Acharya [4], Minseon Park [1], Mario Stevenson [5], Siddappa N. Byrareddy [4], and Michal Toborek [1]

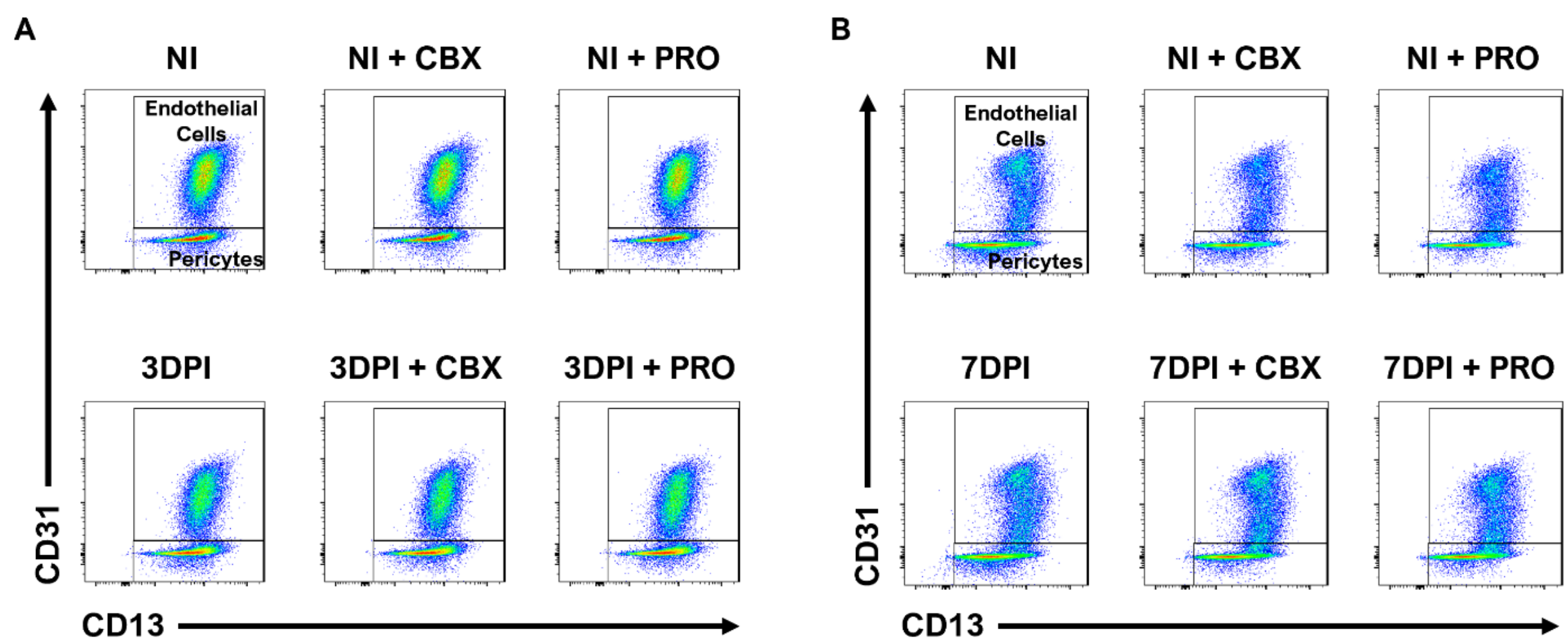

Supplementary Fig. 1 Gating strategy for flow cytometry experiments. CD31 and CD13 gating to differentiate between pericytes (CD13 positive) and endothelial cells (double positive) for (A) 3-days and (B) 7-days. One representative graph is shown for each experimental condition.
